# COVID-19 risk, course and outcome in people with mental disorders: a systematic review and meta-analyses

**DOI:** 10.1017/S2045796023000719

**Published:** 2023-10-20

**Authors:** Patricio Molero, Gabriel Reina, Jan Dirk Blom, Miguel Ángel Martínez-González, Aischa Reinken, E. Ronald de Kloet, Marc L. Molendijk

**Affiliations:** 1 Department of Psychiatry and Clinical Psychology, Clínica Universidad de Navarra, Pamplona, Spain; 2 Navarra Institute for Health Research (IdiSNA), Pamplona, Spain; 3 Department of Microbiology, Clínica Universidad de Navarra, Pamplona, Spain; 4 Institute of Psychology, Department of Clinical Psychology, Leiden University, Leiden, The Netherlands; 5 Outpatient Clinic for Uncommon Psychiatric Syndromes, Parnassia Psychiatric Institute, The Hague, The Netherlands; 6 Department of Psychiatry, University Medical Centre Groningen, University of Groningen, Groningen, The Netherlands; 7 Department of Preventive Medicine and Public Health, School of Medicine, University of Navarra, Pamplona, Spain; 8 CIBER-OBN, Instituto de Salud Carlos III, Madrid, Spain; 9 Department of Nutrition, Harvard TH Chan School of Public Health, Boston, MA, USA; 10 Division of Endocrinology, Department of Internal Medicine, Leiden University Medical Center, Leiden, The Netherlands; 11 Leiden Institute for Brain and Cognition, Leiden University Medical Center, Leiden, The Netherlands

**Keywords:** corona virus disease, COVID-19, disease course, infection risk, long COVID, mental disorders, substance use disorders

## Abstract

**Aims:**

It has been suggested that people with mental disorders have an elevated risk to acquire severe acute respiratory syndrome coronavirus 2 and to be disproportionally affected by coronavirus disease 19 (COVID-19) once infected. We aimed to analyse the COVID-19 infection rate, course and outcome, including mortality and long COVID, in people with anxiety, depressive, neurodevelopmental, schizophrenia spectrum and substance use disorders relative to control subjects without these disorders.

**Methods:**

This study constitutes a preregistered systematic review and random-effects frequentist and Bayesian meta-analyses. Major databases were searched up until 27 June 2023.

**Results:**

Eighty-one original articles were included reporting 304 cross-sectional and prospective effect size estimates (median *n* per effect-size = 114837) regarding associations of interest. Infection risk was not significantly increased for any mental disorder that we investigated relative to samples of people without these disorders. The course of COVID-19, however, is relatively severe, and long COVID and COVID-19-related hospitalization are more likely in all patient samples that we investigated. The odds of dying from COVID-19 were high in people with most types of mental disorders, except for those with anxiety and neurodevelopmental disorders relative to non-patient samples (pooled ORs range, 1.26–2.57). Bayesian analyses confirmed the findings from the frequentist approach and complemented them with estimates of the strength of evidence.

**Conclusions:**

Once infected, people with pre-existing mental disorders are at an elevated risk for a severe COVID-19 course and outcome, including long COVID and mortality, relative to people without pre-existing mental disorders, despite an infection risk not significantly increased.

## Introduction

Severe acute respiratory syndrome coronavirus 2 (SARS-CoV-2) is a highly transmissible respiratory pathogen (Li *et al.*, 2020) that causes coronavirus disease 19 (COVID-19). Although COVID-19, as of May 2023, is not a global emergency anymore (Solis *et al.*, 2020; World Health Organization, 2022), it remains a pandemic (World Health Organization, 2023) causing distress, morbidity and mortality. Furthermore, the condition of long COVID, referring to persistent symptoms such as cognitive impairment, fatigue and low mood, poses an emerging challenge (Suran, 2023). Little is known about the risk factors of long COVID, and the role of mental health in the modulation of that risk is unclear, given that although a pre-existing mental health diagnosis may be an independent risk factor (Lam *et al.*, 2023), it may also mitigate the psychological burden of long COVID (Rastogi *et al.*, 2023).

There are inter-individual differences when it comes to SARS-CoV-2 susceptibility, the course that COVID-19 takes and its outcome. Compromised immune functioning, poor health behaviour, sleep, somatic comorbidities and exposure to chronic stress, all related to infection risk and disease course, are often present in people with poor mental health (Chireh *et al.*, 2019; Chrousos, 2009). Therefore, they may be susceptible to SARS-CoV-2 and a relatively poor COVID-19 course (Simon *et al.*, 2021).

An early meta-analysis on the potential effects of (pre-existing) mood disorders on SARS-CoV-2 infection risk found no evidence for the existence of this association (Ceban *et al.*, 2021). However, the authors did find that having a pre-existing mood disorder was associated with increased chances of COVID-19-related hospitalization and death. This is in line with 3 other meta-analyses reporting a relatively poor COVID-19 course in people with (pre-existing) mental disorders (Fond *et al.*, 2021a; Toubasi *et al.*, 2021; Vai *et al.*, 2021). The meta-analyses unequivocally found high levels of between-study heterogeneity in outcome, which remained unexplained. Given that behavioural parameters related to susceptibility (e.g., poor social distancing) and health behaviour may be different in people with various kinds of mental disorders, we considered an updated meta-analysis stratified by type of mental disorder valuable at this stage. An additional reason for an update is that dozens of primary studies have become available since the publication of the earlier meta-analyses. Therefore, we here aim to optimize and specify the findings of earlier studies using a new and broader systematic review and meta-analysis. We will apply a frequentist (i.e., classical) and Bayesian approach to meta-analysis. The frequentist approach will be used to provide effect-size estimates and for significance testing of the null hypothesis. The Bayesian approach complements this with the strength of evidence estimates for the null and the alternative hypotheses (Heck *et al.*, 2022; Keysers *et al.*, 2020).

We hypothesize that SARS-CoV-2 infection risk, including breakthrough infection, and COVID-19 course, including long COVID, are worse and that mortality rates and the need for COVID-19-related care are high for people with mental disorders relative to people without these disorders. We will explore and review potential explanations, with an emphasis on variables that are open to change such as stress coping and dietary behaviour. Finally, we will discuss the implications for prevention and treatment.

## Methods

We followed the MOOSE (Stroup *et al.*, 2000) and PRISMA guidelines (Moher *et al.*, 2009), and drafted and preregistered a review protocol at the website of the Open Science Foundation (https://osf.io/35jhm/registrations).

### Identification and selection strategy

Web-based searches were performed in PubMed, EMBASE, Web of Science and Google Scholar, which is the optimal database combination for a systematic literature search according to Bramer *et al.* (2017). Database-specific search strings are presented in the online supplement. The final date for the systematic search was 27 June 2023. Reference lists of reviews and meta-analyses were hand-searched for eligible data. A grey literature search on the preprint servers PsyArXiv.org and MedArXiv.org was also performed. Decisions on eligibility were based on titles and abstracts of candidate papers, and ultimately on full-text assessment. At least two members of the review team made a final decision on the eligibility of these articles, based on the in- and exclusion criteria.

### Inclusion and exclusion criteria

Articles were included when they (1) reported SARS-CoV-2 infection risk and/or course of COVID-19 for people with pre-existing mental disorders versus controls without these disorders, and (2) were written in English, German, French, Spanish, Arabic or Dutch. Articles were excluded when (1) no relevant outcome data could be extracted, even after we had been in contact (or had made reasonable attempts hereto) with the corresponding author of the article, or when (2) no original data were reported (e.g., opinion pieces). When articles reported on overlapping data sets, we included the article that was most informative for our purposes (see supplementary material, Box S1 for the rules that we set for article selection and the efforts that were undertaken to avoid the multiple inclusion of a single dataset).

### Exposure and outcome variables

Exposure variables were pre-existing anxiety, depressive, neurodevelopmental, schizophrenia spectrum and substance use disorders (SUDs) assessed according to diagnostic systems such as Diagnostic and Statistical Manual of Mental Disorders, Fourth or Fifth Edition (DSM-IV or V) (American Psychiatric Association, 2013; World Health Organization, 1992) or International Statistical Classification of Disease and Related Health Problems, Tenth Revision (ICD-10) (WHO, 1992). Control conditions were composed of people without the mental disorders of our interest (e.g., the comparison of people with a SUD vs. people without a SUD) and not per se people without any disorder (i.e., the *healthy control*). Outcome variables included (1) infection risk – including break-through infection –, presented as a percentage of SARS-CoV-2 positive tests/self-reports in the populations under study and (2) COVID-19 course variables, further specified as (a) indicators of the severity of the disease (e.g., symptomatic vs. non-symptomatic, requiring respiratory assistance or not), (b) hospitalization rates, (c) intensive care unit (ICU) admission rates, (d) presence of symptoms of long-COVID/persistent COVID-19 symptoms of any kind (Byrne, 2022) and (e) COVID-19-related mortality rates. Please note that at the time of preregistration at the OSF, we considered the outcome variable long COVID as part of the course variables and not as a separate one.

### Data extraction

From included papers, we extracted demographic data (e.g., gender distribution), clinical data (e.g., diagnosis), suspected virus type (estimated from the time frame in which the data were collected), methodological data (e.g., study types) and outcome data (i.e., raw numbers or effect-size estimates and corresponding 95% confidence intervals (95% CIs) on outcome data). Data extraction was performed independently by two members (AR, TY (research assistant), MM) of the review team.

### Measures of effect

We extracted odds ratios (ORs) and corresponding 95% CIs as measures of effect. Where reported, we extracted data from analyses that controlled for the largest number of potential confounders or that came from propensity-matched samples. When results were reported as hazard ratios (HRs) or risk ratios (RRs) and raw data were not available, we interpreted these as an OR when the incidence of the reported outcome was <20%. HRs and RRs based on data reporting on an incidence of the outcome of ≥20% were transformed (Davies *et al.*, 1998; Grant, 2014; Zhang and Yu, 1998).

### Assessment of methodological quality

The methodological quality of input studies was scored by two members of the review team using the *Quality Assessment Tool for Observational Cohort and Cross-sectional Studies* recommended by the United States National Institutes of Health (2021). The items of this instrument are presented as supplement (Table S1).

### Statistical analysis

We performed analyses in Stata version 17 (StataCorp, 2021) and JASP (Jeffreys’s Amazing Statistics Program; JASP team, 2017) and created summary tables on the characteristics of the included studies. Random-effects meta-analyses were used to pool the data on SARS-CoV-2 infection rates, breakthrough infection rates, COVID-19 course, hospitalizations, ICU admissions, long COVID and mortality rates to pre-existing mental disorders. Statistical significance was set at *P* < 0.05. Heterogeneity among studies was quantified using the *I*^2^ measure and assessed for statistical significance using the *Q*^2^-statistic (Sterne *et al.*, 2001). When heterogeneity in outcome was present, subgroup and meta-regression analyses were performed to identify study characteristics that might explain the heterogeneity. Potential continuous variables that served as candidates were gender (% females), average age, methodological quality scores and sample size (*N*). Sensitivity analyses were used to assess the potential effects of controlling for confounders and the time frame of diagnostic assessment (e.g., lifetime vs. current) and virus variant (e.g., Alpha vs. Beta). Data on the latter variable were estimated based on the time frame in which the data were gathered and the geographic location where this was done. We followed the advice of the Cochrane handbook to interpret results from sensitivity and moderator only when there were at least 10 studies available per meta-regression analysis (Higgins and Thomas, 2021). Note that analyses on main outcomes (e.g., mortality) were all a priori registered. Most meta-regression analyses and subgroup analyses were not. Publication bias was assessed using visual inspection of funnel plots and the Eggers regression test (Sterne *et al.*, 2001). All frequentist meta-analyses were repeated by making use of a Bayesian random-effects approach to confirm the robustness of results and to present, using a Bayes Factor (BF_10_), the strength of evidence for the null or the alternative hypothesis. BFs were calculated for an effect-size estimate (logOR) of 0.00 with a standard deviation of 0.50. We used the thresholds suggested by Heck *et al.* (2022) for the interpretation of the BF_10_s.

## Results

**Figure 1.**
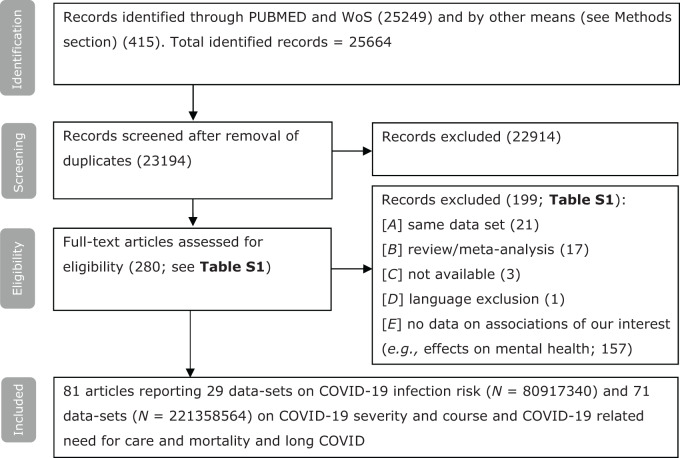
Flow chart on identification, screening and inclusion of eligible publications.

Table S2 (online supplement) lists all the articles that were included for full-text assessment as well as reasons for inclusion and exclusion. See Figure 1 for a flow chart and further information on the identification, screening and inclusion of studies. Eighty-one of the 23,194 candidate articles (0.4%) reported data that met the eligibility criteria. Fourteen of the articles reported data from a prospective design (17%) and 68 from a retrospective design (83%) (see Table S3). More than three-quarter of the data included here were not included in any of the four previous meta-analyses (Ceban *et al.*, 2021; Fond *et al.*, 2021a; Toubasi *et al.*, 2021; Vai *et al.*, 2021; see Table S4). The surplus of articles that we report on results from an accretion of primary articles and from differences in inclusion criteria. In Box S2, we describe some specific actions that were undertaken to avoid data overlap within the analysis.

Table 1 provides demographic and clinical information on the samples from the input studies that we included. Tables S3–S5 provide further information on the method and assessment of predictor variables, outcome variables (S5), control conditions (S3), virus variants (S4), COVID-19 diagnostics (S5) and covariates used in analyses (S3). All except three studies reported on the comparison of people with a specific disorder versus people without this disorder. COVID-19 data were serologically confirmed in all studies, with two exceptions in which the diagnosis was gathered by self-diagnosis or through self-report. Psychiatric diagnostic spectra were most often defined by ICD-10 criteria (World Health Organization, 1992) followed by DSM-IV and V criteria (American Psychiatric Association, 1994, 2013).

**Table 1.** Characteristics of the studies included and samples by outcome

	*N*	Age	% Female	Predictors	Country
*SARS-CoV-2 infection rate*					
Al-Aly *et al.*, 2022	33,940	71 ^AV.^	8	Several categories	USA
Allen *et al.*, 2020	188,653	50 ^MED.^	57	SUD	USA
Amin *et al.*, 2022	96	26 ^MED.^	38	Psychosis spectrum	Indonesia
Azar *et al.*, 2020	14,036	51 ^AV.^	61	Depression	USA
Bailey *et al.*, 2021	135,794	9 ^AV.^	47	Mental disorders	USA
Canal-Rivero *et al.*, 2021	558,274	48 ^AV.^	36	Several categories	Spain
Cohen *et al.*, 2022	64,409	12 ^AV.^	48	ADHD	Israel
Dai *et al.*, 2022	473,958	68 ^AV.^	55	Mental disorders	UK
De Vito *et al.*, 2021	382	81 ^AV.^	63	Mental disorders	Italy
Egede *et al.*, 2021	30,976	60 ^MED.^	53	Several categories	USA
Goldberger *et al.*, 2022	125,273	NK	NK	Psychosis spectrum	Israel
Haimovich *et al.*, 2020	2182	65 ^AV.^	48	Several categories	USA
Lebin *et al.*, 2020	5419	52 ^AV.^	16	AUD	USA
Lee *et al.*, 2021 (1)	48,449	60 ^AV.^	55	Several categories	South Korea
Merzon *et al.*, 2020 (1)	14,022	39 ^AV.^	48	Several categories	Israel
Nemani *et al.*, 2021a (1)	1958	47 ^MED.^	54	Psychosis spectrum	USA
Nishimi *et al.* 2022^a^	263,697	66 ^AV.^	9	Several categories	USA
Orlando *et al.*, 2021	20,855	60 ^MED.^	44	Several categories	Italy
Tang *et al.*, 2020	1970	73 ^AV.^	55	Depression	USA
Taquet *et al.*, 2021	1,729,837	50 ^MED.^	55	Several categories	USA
Teixeira *et al.*, 2020	2,535,098	44 ^AV.^	62	Several categories	USA
Tzur-Bitan *et al.*, 2021	51,078	51 ^AV.^	61	Psychosis spectrum	Israel
Varela Rodríguez *et al.*, 2021	188	60 ^AV.^	29	AUD	Spain
Wang *et al.*, 2021a	73,099,850	42 ^MED.^	54	SUD	USA
Wang *et al.*, 2021b	473,958	68 ^AV.^	55	Several categories	UK
Wang *et al.*, 2022a^a^	597,392	42 ^MED.^	54	SUD	USA
Yang *et al.*, 2021	421,014	68 ^AV.^	55	Several categories	UK
*COVID-19 severity or mortality*					
Al-Aly *et al.*, 2022^b^	33,940	71 ^AV.^	8	Several categories	USA
Allen *et al.*, 2020	11830	50 ^MED.^	57	SUD	USA
Azar *et al.*, 2020	1052	53 ^AV.^	51	Depression	USA
Baillargeon *et al.*, 2021	11,124	54 ^AV.^	53	SUD	USA
Barcella *et al.*, 2021	144,321	42 ^MED.^	55	Several categories	Denmark
Bayrak and Çadirci, 2021	122	73 ^AV.^	48	Depression	Turkey
Bellan *et al.*, 2022^b^	324	60 ^AV.^	59	Several categories	Italy
Bhopalwala *et al.*, 2022	1626	64 ^MED.^	50	SUD	USA
Canal-Rivero *et al.*, 2021	698	48 ^AV.^	36	Severe disorders	Spain
Castro *et al.*, 2021	2988	59 ^AV.^	47	Several categories	USA
Catalan *et al.*, 2023	157,246	44 ^AV^	53	Several categories	Spain
Cavallaro *et al.*, 2021	13,954	65 ^MED.^	41	Serious illness	UK
Chang *et al.*, 2020	710,980	74 ^MED.^	55	Depression	USA
Chen *et al.*, 2021a (1)	1331	47 ^AV.^	38	Severe illness	UK
Chen *et al.*, 2021b (2)	7445	79 ^AV.^	58	Severe illness	UK
Clift *et al.*, 2020	6,083,102	48 ^MED.^	51	Severe illness	UK
Clouston *et al.*, 2021	1375	60 ^AV.^	43	Depression	USA
Cummins *et al.*, 2021	1781	60 ^MED.^	45	Several categories	UK
Descamps *et al.*, 2022	97,452	57 ^MED.^	47	Several categories	France
de Miranda *et al.*, 2022^b^	646	50 ^AV.^	54	Depression	Brazil
De Vito *et al.*, 2021	382	81 ^AV.^	63	Mental disorders	Italy
Díaz-Simón *et al.*, 2021	15,034	42 ^MED.^	40	AUD	Spain
Diez-Quevedo *et al.*, 2021	2150	61 ^AV.^	43	Several categories	Spain
Durstenfeld *et al.*, 2023^b^	1480	53 ^AV.^	69	Several categories	USA
Egede *et al.*, 2021	2103	60 ^MED.^	53	Several categories	USA
Fond *et al.*, 2021b	50,750	72 ^MED.^	43	Psychosis spectrum	France
Francis *et al.*, 2021	900	52 ^AV.^	53	Mental health	UK
Gasnier *et al.*, 2022	177	57 ^AV.^	31	Mental disorders	France
Giannoglou *et al.*, 2020	512	60 ^AV.^	38	Mental disorders	Greece
Goldberger *et al.*, 2022	125,273	NK	NK	Mental disorders	Israel
Hashemi-Shahri *et al.*, 2022	413	45 ^AV.^	45	Mental disorders	Iran
Hedberg *et al.*, 2023^b^	204,805	NK	NK	Mental disorders	Sweden
Hirashima *et al.*, 2021	61	48 ^AV.^	34	Mental disorders	Japan
Izurieta *et al.*, 2021	25,333,329	72 ^MED.^	56	Depression	USA
Jeon *et al.*, 2020	230,565	55 ^AV.^	54	Several categories	South Korea
Jones *et al.*, 2021^b^	3151	52 ^AV.^	64	Mental disorders	UK
Kundi *et al.*, 2020	18,234	74 ^AV.^	53	Several categories	Turkey
Lee *et al.*, 2021 (2)	814	75 ^AV.^	51	Several categories	South Korea
Lega *et al.*, 2021	6607	78 ^AV.^	35	Several categories	Italy
Li *et al.*, 2022	205	58 ^AV.^	48	Mental disorders	China
Maripuu *et al.*, 2021	7,923,859^b^	50 ^MED.^	49	Severe disorders	Sweden
Merzon *et al.*, 2021 (2)	1870	29 ^MED.^	51	Several categories	Israel
Musheyev *et al.*, 2021	317	63 ^AV.^	47	Mental disorders	USA
Nemani *et al.*, 2021a (1)	7348	57 ^MED.^	54	Several categories	USA
Nemani *et al.*, 2021b (2)	464	57 ^AV.^	48	Psychosis spectrum	USA
Nilsson *et al.*, 2022	82,171	48 ^AV.^	47	Several categories	Denmark
Orlando *et al.*, 2021	3497	60 ^MED.^	44	Several categories	Italy
Pavarin *et al.*, 2022	676,082	NK	NK	SUD	Italy
Poblador-Plou *et al.*, 2020	4412	59 ^MED^	68	Several categories	Spain
Qeadan *et al.*, 2021	52,312	53 ^MED.^	51	SUD	USA
Rodríguez-Molinero *et al.*, 2020	418	65 ^AV.^	54	Several categories	Spain
Salvatore *et al.* 2021	2582	45 ^AV.^	56	Several categories	USA
Sisó-Almirall *et al.*, 2020	322	57 ^AV.^	50	Depression	Spain
Tang *et al.*, 2020	1970	75 ^AV.^	60	Depression	USA
Teixeira *et al.*, 2020	2,535,098	44 ^AV.^	62	Several categories	USA
Thompson *et al.*, 2022^b^	1,064,491	NK	NK	Mental disorders	UK
Tokuda *et al.*, 2023	67,348	54 ^AV.^	43	Mental disorders	Japan
Tzur-Bitan *et al.*, 2021	51,078	51 ^AV.^	61	Psychosis spectrum	Israel
Varela Rodríguez *et al.*, 2021	188	60 ^AV.^	29	AUD	Spain
Velásquez García *et al.*, 2021	56,874	36 ^AV.^	NK	Several categories	Canada
Vrotsou *et al.*, 2021	14,197	54 ^AV.^	61	Several categories	Spain
Wang *et al.*, 2021 (2)	61,783,950	NK	54	Several categories	USA
Wang *et al.*, 2021 (1)	73,099,850	42 ^MED.^	54	SUD	USA
Wang *et al.*, 2021 (3)	14,877	54 ^AV.^	45	Several categories	UK
Wang *et al.*, 2022b and c	54,781	54 ^AV.^	97	Several categories	USA
(1 and 2^b^)					
Welch, 2021	5711	74 ^MED.^	45	Mental health	Various
Yang *et al.*, 2020	421,014	68 ^AV.^	55	Several categories	UK
Yanover *et al.*, 2020	4353	34 ^MED.^	45	Depression	Israel

*Abbreviations*: AUD = alcohol use disorder; AV = average, MED = median, NK = not known, SUD = substance use disorder; Mental disorders; the study groups and reports on several mental disorders as 1 group; Several categories, the study reports separate associations on several categories of mental disorders.

a Includes data on breakthrough infection.

b Includes data on long COVID/persistent symptoms.

The median and mean ages of the included samples ranged between 9 and 81 years. The percentage of females per sample ranged from 16% to 86%. The median sample size per analysis was 114837 (range, 61–73,099,850). The methodological quality of most input studies was moderate to high (see Table S6 for scores per study).

### SARS-CoV-2 infection risk

SARS-CoV-2 infection risk was not significantly different in populations of people with a mental disorder versus those in populations without a mental disorder (see Table 2). Subcategories of neurodevelopmental disorders (i.e., autism spectrum disorder and attention-deficit/hyperactivity disorder (ADHD)) and mood disorders (i.e., depressive disorder and bipolar disorder) were tested with regard to outcome, but neither of these was associated with an increased risk. Bayesian meta-analyses yielded BFs suggesting either anecdotal or moderate evidence favouring the null hypothesis of *no effect of mental disorder on the risk to acquire SARS-CoV-2* (see Table 2).

**Table 2.** Results from frequentist and Bayesian meta-analyses

SARS-CoV-2 infection risk	*K*^a^	*N*	OR (95% CI)	BF_10_ for OR = 1.00^b^	*I^2^*	Egger’s *t*
Anxiety disorders	4	3,060,903	1.20 (0.99–1.46)	0.80 + H_0_	95.0***	8.54
Neurodevelopmental disorders	4	566,411	1.29 (0.87–1.90)	0.79 + H_0_	85.8***	0.72
Mood disorders	7	9,247,673	0.87 (0.68–1.11)	0.47 + H_0_	98.3***	12.5
Psychosis spectrum disorder	6	61,783,950	1.06 (0.80–1.41)	0.72 + H_0_	89.1***	4.85**
Substance use disorder	9	78,236,869	1.09 (0.75–1.59)	0.32 ++ H_0_	99.6***	1.06
Mix/other	11	7,354,951	1.09 (0.81–1.47)	0.37 + H_0_	99.2***	−1.62
*COVID-19 severity*/course	*k*	*N*	OR (95% CI)	BF_10_ for OR = 1.00^a^	*I^2^*	Egger’s *t*
Anxiety disorder	4	100,072	1.23 (1.12–1.36)***	1.67 + H_1_	24.0	−0.04
Neurodevelopmental disorders	1	1780	1.81 (1.29–2.25)**	*No data*	*No data*	*No data*
Mood disorders	7	250,230	1.65 (1.22–2.23)**	6.93 ++ H_1_	88.3***	−1.30
Psychosis spectrum disorder	5	244,758	1.76 (1.06–2.92)*	2.88 + H_1_	80.5***	−0.79
Substance use disorder	7	122,592	1.51 (1.24–1.83)***	52.31 ++++ H_1_	64.9**	2.42**
Mix/other	10	258,947	1.35 (1.11–1.64)**	7.16 ++ H_1_	56.9*	0.65
*COVID-19 hospitalization*	*k*	*N*	OR (95% CI)	BF_10_ for OR = 1.00^a^	*I^2^*	Egger’s *t*
Anxiety disorder	4	633,041	1.44 (1.17–1.78)**	3.99 ++ H_1_	76.7*	−4.63
Neurodevelopmental disorders	1	1780	1.93 (1.06–3.51)*	*No data*	*No data*	*No data*
Mood disorders	6	28983811	1.63 (1.34 to 1.97)***	169.93 ++++ H_1_	92.5***	2.21*
Psychosis spectrum disorder	10	36,522,290	1.86 (1.32–2.62)***	155.98 ++++ H_1_	98.5***	−0.72
Substance use disorder	7	914,933	1.54 (1.44–1.69)***	939.15 ++++ H_1_	69.4**	−0.69
Mix/other	5	9,319,916	1.55 (1.16–2.07)**	3.45 ++ H_1_	98.1***	−0.76
*COVID-19 ICU admission*	*k*	*N*	OR (95% CI)	BF_10_ for OR = 1.00^a^	*I^2^*	Egger’s *t*
Anxiety disorder	2	100,034	1.08 (0.76–1.54)	*No data*	*No data*	*No data*
Neurodevelopmental disorders	0					
Mood disorders	6	6,246,991	1.11 (0.84–1.46)	0.32 ++ H_0_	49.9	−0.60
Psychosis spectrum disorder	6	290,253	1.45 (0.98–2.15)	2.04 + H_0_	93.6**	0.90
Substance use disorder	4	194,035	1.59 (1.11–2.28)*	52.30 +++ H_1_	83.0***	0.02
Mix/other	9	6,384,393	1.34 (1.02–1.75)*	7.16 ++ H_1_	93.7***	−0.26
*COVID-19 mortality*	*k*	*N*	OR (95% CI)	BF_10_ for OR = 1.00^a^	*I^2^*	Egger’s *t*
Anxiety disorder	6	3,215,223	1.14 (0.72–1.80)	0.48 + H_0_	91.4***	0.07
Neurodevelopmental disorders	2	4412	1.26 (0.77–2.05)	0.65 + H_0_	0.0	*No data*
Mood disorders	14	34,395,611	1.50 (1.31–1.71)***	453.81 ++++ H_1_	79.2***	1.67
Psychosis spectrum disorder	13	57,137,783	2.15 (1.68–2.75)***	2923.57 ++++ H_1_	88.4***	−0.08
Substance use disorder	11	1,094,627	1.45 (1.12–1.87)***	13.49 +++ H_1_	86.0***	0.47
Mix/other	20	6,787,842	1.26 (1.08–1.47)***	4.58 ++ H_1_	81.4***	−0.83

* *P* < 0.05, **P < 0.01, ***P < 0.001.

a Estimates come from analyses including nationwide data, at the expense of local data. Estimates from analyses favouring local data (e.g., data from studies performed in North-East London, Manchester and Bristol) over data from the UK Biobank over nationwide data (e.g., the UK Biobank) in different combinations is presented in Tables S6 and S7. In all cases, pooled effect sizes fell within in the 95% CIs when the nationwide data was replaced by more local data.

b Evidence category for the results from Bayesian analyses: + anecdotal evidence for H**_0_** or H**_1_**; ++ moderate evidence for H**_0_** or H**_1_**; +++ strong evidence for H**_0_** or H**_1_**; ++++ very strong evidence for H**_0_** or H**_1_**

Between-study heterogeneity in outcome was evident in all analyses (see Table 2). Analysis by time frame of diagnostic assessment (e.g., lifetime vs. past year/current) and statistical control for confounders by input studies (categorical; yes vs. no) yielded similar results. The continuous variables that were tested as effect moderators (i.e., percentage of females, average age, methodological quality and sample size) were not associated with outcome (see Table S8). We found evidence for publication bias in the data on psychosis spectrum disorders and infection risk (see Table 2). Accounting for this using trim-and-fill methods did not result in a different estimate. We estimated per sample the dominant virus variant for each specific study. About 80% of studies investigated the Alpha variant and the remaining studies were either a mix of the Alpha and Beta variant or the Beta variant. There was no evidence that the observed associations were driven by a specific virus variant. Overall, results from analyses on local and nationwide data were similar (see Table S7 and dataset S1).

Two studies specifically compared breakthrough infection risk in people with a mental disorder versus controls without the mental disorders under investigation (Nishimi *et al.*, 2022; Wang *et al.*, 2022c). Both studies gathered nationwide data in the United States. To avoid pooling of dependent data, statistical analyses were not performed and a narrative review of the results will be presented. Nishimi *et al.* (2022) show, based on data from the department of veteran affairs (*N* = 263,697), a small significant increase in the risk of breakthrough infection in patients with several kinds of mental disorders (range adjusted relative RRs: 1.03–1.16). Wang *et al.* (2022b) report data on 579,372 individuals from the TriNetX network showing an overall increased breakthrough infection risk in patients with several types of SUDs relative in comparison to propensity-matched controls who were free of SUDs (range HRs: 1.06–2.06).

### COVID-19 severity

Consistent over all categories, we find that people with pre-existing mental disorders show higher odds to experience a severe COVID-19 course, once infected, relative to people in control conditions (pooled ORs range, 1.26–2.32; see Table 2). Bayesian meta-analysis aligned with these results and show that the evidence favouring the alternative hypothesis is in the range from anecdotal (for anxiety disorders) to very strong (for SUDs) (see Table 2).

### COVID-19-related hospitalization, ICU admission and mortality

Associations with COVID-19-related hospitalization, ICU admission and mortality were also largely consistent over predictor categories, with higher odds for people in the patient categories relative to people in the control condition who were free of the specific diagnosis that we related to outcome (pooled ORs range, 1.05–1.93). Results from Bayesian meta-analys in line with the results from those derived through the classical frequentist approach. The evidence for the hypotheses of the existence of an association between having mental disorders and COVID-19-related hospitalization and morbidity was notably strong in most cases. For the outcome of ICU admission, the level of evidence was only anecdotal or moderate (see Table 2).

Between-study heterogeneity in outcome was evident in most analyses (see Table 2). Results over subcategories (e.g., bipolar and unipolar depression for mood disorders) were largely similar. Moreover, results were not driven by a particular virus variant. The time frame of psychiatric assessment (e.g., lifetime vs. past year/current) and statistical control for confounders by input studies (categorical; yes vs. no) was not differentially associated with changes in outcome. There were two exceptions. One, the association between mood disorders and hospitalization was stronger for people with a current versus a lifetime disorder (*z* = 2.85, *P* < 0.01). Two, uncontrolled studies investigating the association between psychosis spectrum disorder and COVID-19-related mortality yielded higher effect-size estimates relative to studies that applied statistical control (e.g., matching, the use of co-variates) (*z* = 5.83, *P* < 0.001). Analyses of local and nationwide data yielded consistent results (see Table S9 and dataset S1). Associations were not moderated by geographic location, average age, percentage of females and methodological quality (see Table S10). In a few analyses, we found evidence of publication bias (Table 2). Controlling for this using trim-and-fill procedures did not result in substantially different estimates (data not shown).

### Long COVID

Eight studies reported on the association between pre-existing mental disorders and long COVID/persistent symptoms. [Al-Aly *et al.,* 2022; Bellan *et al.,* 2022; de Miranda *et al.,* 2022; Durstenfeld *et al.,* 2023; Hedberg *et al.,* 2023; Jones *et al.,* 2021; Thompson *et al.,* 2022; and Wang *et al.,* 2022a (total *N* = 6,350,939)]. Relative to the other outcomes, these studies on long COVID quite often applied a prospective design (50% vs. 11%). Both Jones *et al.* and Thompson *et al.* report on UK nationwide primary care data. To avoid overlapping data, we once ran a meta-analysis with Jones *et al.* included and Thompson *et al.* excluded and one with Jones *et al.* excluded and Thompson *et al.* included. Al-Aly *et al.* (2022) and Wang *et al.* (2022a) report on two large US-based cohorts. These however were not deemed to present overlapping data due to the populations under study, which were composed of veterans and healthcare professionals respectively. All the included data sets showed that pre-existing mental disorders are associated with the presence of long COVID, although between-study heterogeneity in outcome was present (*P*-values < 0.001). Pooled ORs ranged between 1.68 and 1.75, with corresponding *P*-values < 0.0001. Bayesian analyses showed that the evidence for the alternative hypothesis of a negative effect of pre-existing mental disorders on long COVID symptoms is extremely strong (BF_10_ range, 205–421). Due to a lack of data, we were not able to run analyses per type of mental disorder.

## Discussion

Our meta-analysis of 81 original studies and a total of 304 effect size estimates shows that people with pre-existing mental disorders are not more likely to acquire SARS-CoV-2 relative to control conditions of people without these disorders. However, people with pre-existing mental disorders have increased morbidity and mortality rates and need for care related to COVID-19 once infected, and long COVID symptoms relative to people without pre-existing mental disorders. Although effect-size estimates are small to medium, Bayesian analyses showed that in many cases the evidence favouring the alternative hypothesis is very strong (e.g., mortality and hospitalization rates for people with psychosis spectrum disorder and SUDs).

### Pre-existing mental disorders and susceptibility to SARS-CoV-2 infection

We expected increased SARS-CoV-2 infection risk in people with pre-existing mental disorders relative to people in control conditions without these disorders. Increased susceptibility however was not evident in the data. Results from Bayesian meta-analyses showed either anecdotal or moderate evidence favouring the null hypothesis of *no effect of mental disorder on the risk to acquire SARS-CoV-2* (see Table 2).

A reason for not finding the expected associations could be that infection is less likely in case of loneliness and social deprivation, which are relatively common in people with mental disorders and even more so during the pandemic (Pai and Vella, 2022). Future studies are needed to address the relevance and contributions of these factors and their potential interactions with risk factors for infection that are evident in people with mental disorders, amongst which are poor physical fitness/co-morbid somatic conditions (Barton *et al.*, 2020), malnutrition (Mahboub *et al.*, 2021), smoking (Yuan *et al.*, 2020), risk-taking behaviours and impulsivity (Kreek *et al.*, 2005) and socioeconomic and minority status (Butler, 2021; Mena *et al.*, 2021).

### Pre-existing mental disorders and morbidity and mortality rates for COVID-19 and long COVID

Another main finding of the current study is that people with most types of pre-existing mental disorders are disproportionally affected by COVID-19 once infected relative to people without pre-existing mental disorders. Our group found similar associations for pre-existing neurodegenerative diseases (Smadi *et al.*, 2023). Theoretically, this might be a direct result of relatively low socioeconomic status, especially when it comes to limitations on access to care. An in-depth analysis of COVID-19-related mortality in the city of Santiago, Chile, showed that people with low socioeconomic status had fewer testing opportunities, faced relatively long waiting lists and struggled with delayed test results. These circumstances correlate with increased morbidity and mortality rates (Mena *et al.*, 2021).

A complementary explanation (Wang *et al.*, 2021) may be that people with (especially severe) mental disorders often lack the motivation, energy and/or insight to go out for testing (e.g., due to disorganization, negative symptoms, cognitive dysfunction or delusional thinking). This may result in late detection and, with that, delayed medical care. Moreover, in this group, too, compromised immune functioning and poor physical fitness probably contribute (Beurel *et al.*, 2020; Chireh *et al.*, 2019; Wei *et al.*, 2020). Studies indicate that, on average, people with mood, schizophrenia spectrum and SUDs have increased inflammatory markers (Beurel *et al.*, 2020; Dowlati *et al.*, 2010; Fraguas *et al.*, 2019) relative to control conditions of people without these disorders and hence an increased vulnerability to SARS-CoV-2 infection.

Clinical data show that a dysregulated – pro-inflammatory – immune status predicts a poorer course and higher mortality rate in patients with COVID-19. Although the exact mechanisms that underlie this association are yet unknown, it has been suggested that a compensatory inflammatory response that dysregulates the adaptive immune response might play a role here (Henry *et al.*, 2019). Therefore, testing the cellular immune response and blood glucose levels has potential prognostic value in COVID-19 patients with pre-existing mental disorders and/or SUDs. In the chronic pro-neuroinflammatory environment that characterizes at least some subcategories of mental disorders, T cells show maladaptive characteristics in terms of a higher CD4/CD8 ratio, along with a decreased cellular immune response in depressive disorder (Toben and Baune, 2015). A lymphocyte-mediated mechanism with an altered CD4/CD8 ratio has been implicated in the pathogenesis of alcohol-related liver injury (Batey *et al.*, 2002), and an altered CD4/CD8 ratio has been observed in people with schizophrenia spectrum disorders (Al-Diwani *et al.*, 2017). Interestingly, an imbalance of the lymphocyte subpopulations, characterized by reduced counts of CD4+ and CD8+ T cells and an increase of natural-killer lymphocytes has been implicated as an early marker of mortality in inpatients with COVID-19 (Cantenys-Molina *et al.*, 2020).

### Allostatic overload

To contextualize the mechanisms referred to above, the concept of *allostatic overload* may be useful. *Allostatic overload* refers to the damage sustained by biological systems due to repeated and/or prolonged stress (McEwen, 2013). Chronic stress plays an important role in the aetiology and disease course of many types of mental disorders (Juster *et al*., 2010; Spitzer *et al*., 2009). Compromised immune functioning and poor physical fitness often go hand in hand with exposure to chronic stress through direct neuro-endocrine routes or indirect routes through stress effects on health behaviour and sleep (Glaser and Kiecolt-Glaser, 2015; McEwen, 2013). Prolonged stressors and excessive activity of stress mediators are associated with glucocorticoid-receptor resistance, which interferes with the appropriate regulation of inflammation, and this is an important aspect in the onset and progression of a wide range of diseases (Cohen *et al.*, 2012; De Kloet *et al.*, 2005), including COVID-19. Glucocorticoid-receptor resistance is linked to higher levels of stress and inflammatory mediators, and hence a sustained pro-inflammatory state (De Kloet *et al.*, 2018; Glaser and Kiecolt-Glaser, 2015; Keller *et al.*, 2017), including immune dysregulation of brain microglia (Wohleb *et al.*, 2016), a characteristic feature of, in particular, atypical or a hypothesized immune-metabolic form of depression (Lamers *et al.*, 2018).

In addition, high blood glucose levels (e.g., due to metabolic syndrome in people with schizophrenia spectrum disorders and major depressive disorders) may be important mediators of COVID-19 progression and severity (Logette *et al.*, 2021). Stress hormones are major and direct determinants of blood glucose levels and as such, stress can set the stage for insulin resistance. Hence, in a worst-case scenario, chronic stress can mediate a COVID-19-vulnerable phenotype (Pal and Bhadada, 2020). Another potentially relevant factor is major histocompatibility complex variation, which has been implicated in the development of schizophrenia spectrum disorders and bipolar disorder (The International Schizophrenia Consortium 2009). This variability may cause dysfunctional T-cell–mediated immune responses, which can contribute to COVID-19 progression, and hence to higher severity and mortality of the infection (Chen and John Wherry, 2020; Müller and Schwarz, 2010).

### Clinical and policy implications

The results of this study have several clinical implications. From a preventive perspective, patients with mental disorders should be considered at high risk for a poor COVID-19 prognosis. This should inform vaccination policies and educational campaigns, especially in areas with limited access to care for these population groups. Given that high levels of blood glucose and pro-inflammatory markers appear to be important mediators of COVID-19 progression and severity (Lamers *et al.*, 2018; Logette *et al.*, 2021), our results are also relevant to the pharmacotherapy of depressive disorders, schizophrenia spectrum disorders and SUDs.

Healthy blood glucose profiles depend at least partly on the type of antidepressant or antipsychotic prescribed. Regarding antidepressants, serotonin reuptake inhibitors exert a moderate beneficial effect on the glucose levels of patients with diabetes mellitus and depression (Baumeister *et al.*, 2012). Likewise, serotonin–noradrenaline reuptake inhibitors (McIntyre *et al.*, 2006), agomelatine and bupropion seem safe (Roopan and Larsen, 2017), whereas tricyclic antidepressants elevate the risk of type-2 diabetes mellitus (Wang *et al.*, 2021c). Since monoaminoxidase inhibitors may promote hypoglycaemia (McIntyre *et al.*, 2006), with these compounds there is a need for optimal control in patients with diabetes. As for antipsychotic drugs, healthier blood glucose profiles can be obtained by prescribing some classic rather than atypical antipsychotics (Libowitz and Nurmi, 2021; Zhang *et al.*, 2013), such as fluphenazine or haloperidol, except for aliphatic phenothiazines (chlorpromazine and levomepromazine (Haupt and Newcomer, 2001). In case atypical antipsychotics are indicated, healthy profiles can be promoted by prescribing aripiprazole (Baker *et al.*, 2009; van Winkel *et al.*, 2008), ziprasidone (Sacher *et al.*, 2008; Simpson *et al.*, 2004) or lurasidone (McEvoy *et al.*, 2013), especially in comparison with olanzapine (Koller and Doraiswamy, 2002), risperidone (Koller *et al.*, 2003), quetiapine (Koller *et al.*, 2001) and clozapine (De Hert *et al.*, 2007). In cases of antipsychotic-related diabetes, the use of standard antidiabetic medication may be helpful (Cernea *et al.*, 2020). This seems especially important in first psychotic episodes and for young patients (Saddichha *et al.*, 2008).

From a nutritional perspective, there is preliminary evidence that ketosis induced by a dietetic intervention may contribute to the mitigation of neuro-inflammation via the inhibition of glutamate activity in astrocytes (Morris *et al.*, 2020). Dietary interventions therefore should also be considered as a protection against COVID-19. Perez-Araluce *et al.* (2021) indicate the relevance of this in a study that reports relatively large risk reductions in people adhering to high-quality Mediterranean diets. A similar point can be made for stress-reduction techniques (Callus *et al.*, 2020).

Our study also underlines the importance of using independent data (Cheung, 2019). A re-analysis that we made of previous studies indicates that Toubasi *et al.* (2021) may have included UK Biobank data twice (Batty and Gale, 2021; Yang *et al.*, 2021). We suspect that Ceban *et al.* (2021) also double-counted UK Biobank data (Van der Meer *et al.*, 2020; Yang *et al.*, 2020), as well as US nationwide data (Taquet *et al.,* 2021; Wang *et al.,* 2021d), while Fond *et al.* (2021a) did so with nationwide data from Korea (Jeon *et al.,* 2020; Lee *et al.*, 2020) and Vai *et al.* (2021) in their analysis on mortality (An *et al.*, 2020; Lee *et al.*, 2020). This double-counting data, can give a false impression of precision (Cheung, 2019) and we put great effort in avoiding this (e.g., see Box S1 in the online supplement).

### Strengths and limitations

Our analyses on SARS-CoV-2 infection rate by mental disorder have precedence in the literature, and the findings that we report on COVID-19 course variables are roughly similar to those reported by previous meta-analyses (Ceban *et al.*, 2021; Fond *et al.*, 2021a; Toubasi *et al.*, 2021; Vai *et al.*, 2021). The dataset that we composed and our analytical approach, however, have some strengths over them. First, we present results stratified by mental disorder and do so for different outcomes. Second, we believe that our approach stands out in the extensive efforts that we took to avoid reporting on overlapping data sets.

Our results indicate the existence of associations between various pre-existing mental disorders and a relatively poor COVID-19 course but do not prove these associations, because the underlying data was purely observational and often was cross-sectional. Vaccination status is a key determinant for all outcomes that we report on. On the one hand, studies indicate that people with mental disorders are less likely to have themselves fully vaccinated (Hartonen *et al.*, 2023; Mazereel *et al.*, 2021). On the other hand, some countries, (e.g., the Netherlands) prioritized vaccination of people with mental disorders. Future studies are necessary to investigate the extent to which vaccination moderates the associations between pre-existing mental disorders and COVID-19 course.

The control conditions that were used in most of the input studies also constitute a limitation. All studies (with three exceptions) reported data on convenience samples in which people with a disorder were compared to people without the disorder (e.g., SUD vs. no SUD). The use of such control conditions comes with limitations. The concept of psychiatric comorbidity is ignored. Associations that we now assign to for instance a SUD, can easily reflect associations that are due to a SUD comorbid with depression or psychosis. Data exist that indeed suggests that patterns of comorbidity can be the driving force underlying the relatively poor COVID-19 course observed in people with mental disorders (Schieber *et al.*, 2023).

The publication time frame of the input studies makes that by far most studies that we included investigated the original SARS-CoV-2 Wuhan Alpha virus. This is an important advantage regarding homogeneity, but also a disadvantage because new variants (i.e., Gamma, Delta, Omicron) could not be considered. The predictor categories in our study were more fine-grained than those in earlier meta-analyses (e.g., Toubasi *et al.*, 2021), but still represent heterogeneous categories (Feczko *et al.*, 2019). Possibly, this resulted in at least some of the between-study differences in outcome. Large samples were included, and our meta-analyses were well-powered, but for moderator and subgroup analyses this may have not been the case. The Cochrane handbook advises interpreting the results derived from meta-regression only when there are ≥10 studies available per analysis (Higgins and Thomas, 2021). Sometimes, we reported results based on fewer studies. Furthermore, we show that covariate-adjusted and matched analyses yield similar results relative to uncontrolled analyses. However, results from controlled and matched analyses still can be confounded in case the full set of covariates is not considered. A good example here is medication status. The use of many types of psychotropic medication could be related to outcome (see above). This however is not specifically addressed in most records for us to exclude this as an alternative explanation. It should be noted here that while the analyses on main outcomes (e.g., mortality) were all *a priori* registered, most meta-regression and subgroup analyses were not. Finally, we included studies that were written in English, German, French, Spanish, Arabic or Dutch and may have missed relevant data because they are written and published only in, e.g., Mandarin.

### Summary and conclusion

The infection risk for SARS-CoV-2 infection is rather similar in people with mental disorders relative to control conditions. Yet, once infected, a more severe COVID-19 course was observed for all mental disorders that we studied. Hospitalization rates were relatively high in people with mental disorders; moreover, they are more likely to die from COVID-19 relative to controls. We conclude that patients with pre-existing mental disorders are (behaviourally and biologically) disadvantaged when it comes to coping with the disease. This conclusion underscores an eminent element in current definitions of *poor mental health*: “not *having the (full) ability to cope with events and challenges*” (Galderisi *et al.*, 2015). The documented increase in the prevalence of mental disorders in the wake of the COVID-19 pandemic (the COVID-19 Mental Disorders Collaborators, 2021; Simon *et al.*, 2021; Taquet *et al.*, 2021) is disturbing, but especially so in the light of our findings. Together, they suggest a dangerous interaction loop. Based on our data, we predict that the stress exerted by the COVID-19 pandemic (including interpersonal stress due to lockdowns, constraints imposed by school or work, and loss) (Acuff *et al.*, 2020) will make this loop even more dangerous because of its effects on disease moderators, e.g., allostatic overload. All in all, our findings underline the importance of vaccine priority and health surveillance in people with mental disorders, in the current and possibly a next pandemic or if vaccine escape mutants arise.

## Supporting information

Molero et al. supplementary material 1 Molero et al. supplementary material

Molero et al. supplementary material 2 Molero et al. supplementary material

## Data Availability

The data presented in this manuscript will be made freely available at the OSF webpage dedicated to this project (https://osf.io/35jhm/) upon acceptance.

## Supplementary material

The supplementary material for this article can be found at https://doi.org/10.1017/S2045796023000719.
