# Supplementary material for: COVID-19 risk, course and outcome in people with mental disorders: a systematic review and meta-analyses
Source: Epidemiol Psychiatr Sci. 2023 Oct 20;32:e61. doi: 10.1017/S2045796023000719 (PMC10594644; doi:10.1017/S2045796023000719)
Supplement: Molero et al. supplementary material 1 — Molero et al. supplementary material [file S2045796023000719sup001.docx]

**Searches:** Systematic searches in PubMed, Web of Science and the preprint server Biorxiv.org. This will be supplemented with a non-systematic search in Google Scholar. Papers written in English, Dutch, Spanish, German, or French will be included. Final search date is August 28 2022. The search string is uploaded below.

**Search terms PubMed:** (“COVID 19” [Title/Abstract] OR COVID-19 [Title/Abstract] OR COVID19[Title/Abstract] OR “SARS CoV-2” [Title/Abstract] OR “Severe Acute Respiratory Syndrome Coronavirus 2”[Title/Abstract] OR coronavirus [Title/Abstract] OR SARS-CoV [Title/Abstract] OR SARS-CoV-2[Title/Abstract]) AND (psychiatry [Title/Abstract] OR mental [Title/Abstract] OR “clinical psychology” [Title/Abstract] OR substance use [Title/Abstract] OR alcohol [Title/Abstract] OR “illegal drugs” [Title/Abstract] OR addiction OR dependence [Title/Abstract] OR depress* [Title/Abstract] OR mood [Title/Abstract] OR “adjustment disorder” [Title/Abstract] OR Bipolar [Title/Abstract] OR mania [Title/Abstract] OR schizophrenia [Title/Abstract] OR psychosis [Title/Abstract] OR psychotic [Title/Abstract] OR anxi* [Title/Abstract] OR PTSD [Title/Abstract] OR “post-traumatic stress disorder” [Title/Abstract] OR “adjustment disorder” [Title/Abstract] OR “somatic symptom disorder” [Title/Abstract] OR “eating disorders” [Title/Abstract] OR “Binge eating” [Title/Abstract] OR anorexia [Title/Abstract] OR ADHD [Title/Abstract] OR “attention deficit hyperactivity disorder” [Title/Abstract] OR “conduct disorder” [Title/Abstract])

**Search terms Web of Science and EMBASE:** TS = (“COVID 19” OR COVID-19 OR COVID19 OR “SARS CoV-2” OR “Severe Acute Respiratory Syndrome Coronavirus 2” OR coronavirus OR SARS-CoV OR SARS-CoV-2) AND TS = (psychiatry OR mental OR “clinical psychology” OR substance use OR alcohol OR “illegal drugs” OR addiction OR dependence OR depress* OR mood OR “adjustment disorder” OR Bipolar OR mania OR schizophrenia OR psychosis OR psychotic OR anxi* OR PTSD OR “post-traumatic stress disorder” OR “adjustment disorder” OR “somatic symptom disorder” OR “eating disorders” OR “Binge eating” OR anorexia OR ADHD OR “attention deficit hyperactivity disorder” OR “conduct disorder”)

| **BOX 1.** Article selection and overlapping data sets: inclusion rules |
| --- |
| Most of the data on these topics comes from (often open access) electronic data bases and articles probably report on overlapping samples. In order to avoid conclusions based on overlapping data - Inclusion of independent data is a crucial assumption in meta-analysis (Cheung 2019) - we checked the geographic region (*e.g.,* city or state) where the data was gathered in case multiple studies reported data that was gathered in a particular country. When we suspected overlap we contacted study authors to ask for overlap in data sets. In case of a confirmatory answer (and in case of no response) we a priori applied the following rules in the selection of articles in order to avoid results that are based on dependent data. Note that some decisions about overlap (notably about nation wide more local data) were made at the time of data-analysis (see the supplemental excel file). The first decision was made based on the level of matching/statistical control of psychiatric- and control sample. We preferred (propensity score) matching based over adjusting for covariates and the latter was preferred over no control. In case it was necessary we made a next decision based on the validity and the specificity of the predictor assessment. Here we preferred a specific diagnostic assessment over self-reported data, which in turn was preferred over categorization based on for example prescription drugs yes *vs.* no. In case no decision could be made based on this, the decision on inclusion was based on sample size, with a preference for the larger sample size. Below we describe our decisions for countries for which potential overlap was an issue.  When multiple studies were performed in a single country, we checked whether there was ground to suspect overlap with regard to specific associations, and in case there was (*e.g.,* 2 studies reporting infection risk fordepressedpatients from electronic data gathered in New York City hospitals), we included the one that was most informative with regard to our purposes. Third, when nationwide data was available for analysis alongside data gathered more locally (*e.g.,* nationwide data from Spain and data from the autonomous Basque region) we ran analyses once with the nationwide data included and the local data excluded and once with the local data included and the nationwide data excluded. Main meta-analysis outcomes are the ones based on the largest pooled samplesize. Similarity of results among these analyses was reported. For moderator and meta-regression analyses, we alsochose the pooled data-set that contained the largest number of participants.  References  Cheung MW. A Guide to Conducting a Meta-Analysis with Non-Independent Effect Sizes. Neuropsychol Rev. 2019 Dec;29(4):387-396. doi: 10.1007/s11065-019-09415-6. Epub 2019 Aug 24. |

*Assessment of methodological quality*

The methodological quality of input studies was scored by 2 members of the author team (PM, AR, and/or MM) using the *quality assessment tool for cross-sectional studies* that is recommended by the United States National Institutes of Health (US NIH 2021; <https://www.nhlbi.nih.gov/health-topics/study-quality-assessment-tools>). The items of this tool are provided below, in **Table S4**.

**Table S1**. Items of the quality assessment tool for cross-sectional and prospective observational studies

| Item |  |
| --- | --- |
| 1 | Was the research question or objective in this paper clearly stated? |
| 2 | Was the study population clearly specified and defined? |
| 3 | Was the participation rate of eligible persons at least 50%? |
| 4 a | Were all the subjects selected or recruited from the same or similar populations (including the same time period)? |
| 4 b | Were inclusion and exclusion criteria for being in the study prespecified and applied uniformly to all participants? |
| 5 | Was a sample size justification, power description, or variance and effect estimates provided? |
| 6 | For the analyses in this paper, were the exposure(s) of interest measured prior to the outcome(s) being measured? |
| 7 | Was the timeframe sufficient so that one could reasonably expect to see an association between exposure and outcome if it existed? |
| 8 | For exposures that can vary in amount or level, did the study examine different levels of the exposure as related to the outcome (e.g., categories of exposure, or exposure measured as continuous variable)? |
| 9 | Were the exposure measures (independent variables) clearly defined, valid, reliable, and implemented consistently across all study participants? |
| 10 | Was the exposure(s) assessed more than once over time? |
| 11 | Were the outcome measures (dependent variables) clearly defined, valid, reliable, and implemented consistently across all study participants? |
| 12 | Were the outcome assessors blinded to the exposure status of participants? |
| 13 | Was loss to follow-up after baseline 20% or less? |
| 14 | Were key potential confounding variables measured and adjusted statistically for their impact on the relationship between exposure(s) and outcome(s)? |

**Table S2**. Articles submitted to full-text assessment for in- vs exclusion. In- and excluded articles are indicated in green and red respectively.

|  | **Study** | **Reason** |
| --- | --- | --- |
|  | Abbasi-Oshaghi *et al.* 2022 | Opinion paper (1 page review) |
| Abbasi-Oshaghi E, Mirzaei F, Khodadadi I. Alcohol Misuse May Increase the Severity of COVID-19 Infections. Disaster medicine and public health preparedness. 2022;16(3):847–8. | | |
|  | Abdalbary *et al.* 2022 | No mental health 🡪 risk/outcome data |
| Abdalbary M, Kakani E, Ahmed Y, Shea M, Neyra JA, El-Husseini A. Characteristics and outcomes of prisoners hospitalized due to COVID-19 disease. Clinical nephrology. 2022;97(4):232–41. | | |
|  | Adamuz *et al.* 2021 | No mental health 🡪 risk/outcome data; poster |
| Adamuz, J., Gonzalez-Samartino, M., Jimenez-Martinez, E., Tapia-Perez, M., Lopez-Jimenez, M.  M., Rodriguez-Fernandez, H., . . . Juve-Udina, M. E. (2021). Risk of acute deterioration and care  complexity individual factors associated with health outcomes in hospitalised patients with COVID  19: a multicentre cohort study. *BMJ Open, 11*(2). doi:10.1136/bmjopen-2020-041726 | | |
|  | Ahmadi *et al.* 2020 | No mental health 🡪 risk/outcome data; poster |
| Ahmadi, N., Roach, J., Pynoos, R. S., Cardenas, J., & Kopelowicz, A. (2020). Clinical outcome of covid-19 patients with and without diagnosed mental health disorder treated at the los angeles county department of health science. *J Am Acad Child Adolesc Psychiatry, 59*(10), S251-S252. doi:10.1016/j.jaac.2020.08.413 | | |
|  | Ahmadi *et al.* 2021 | No mental health 🡪 risk/outcome data |
| Ahmadi MN, Huang BH, Inan-Eroglu E, Hamer M, Stamatakis E. Lifestyle risk factors and infectious disease mortality, including COVID-19, among middle aged and older adults: Evidence from a community-based cohort study in the United Kingdom. Brain Behav Immun. 2021 Apr 30:S0889-1591(21)00180-X. doi: 10.1016/j.bbi.2021.04.022. Epub ahead of print. PMID: 33940153. | | |
|  | Al-Aly *et al.* 2022 | Mental health 🡪 risk/outcome |
| Al-Aly Z, Bowe B, Xie Y. Long COVID after breakthrough SARS-CoV-2 infection. Nature medicine. 2022;28(7):1461–7. | | |
|  | Alizadehsani *et al.* 2020 | No mental health 🡪 risk/outcome data |
| Alizadehsani R, Alizadeh Sani Z, Behjati M, Roshanzamir Z, Hussain S, Abedini N, Hasanzadeh F, Khosravi A, Shoeibi A, Roshanzamir M, Moradnejad P, Nahavandi S, Khozeimeh F, Zare A, Panahiazar M, Acharya UR, Islam SMS. Risk factors prediction, clinical outcomes, and mortality in COVID-19 patients. J Med Virol. 2021 Apr;93(4):2307-2320. doi: 10.1002/jmv.26699. Epub 2020 Dec 17. PMID: 33247599; PMCID: PMC7753243. | | |
|  | Alizadehsani *et al.* 2021 | No mental health 🡪 risk/outcome data |
| Alizadehsani R, Alizadeh Sani Z, Behjati M, Roshanzamir Z, Hussain S, Abedini N, Hasanzadeh F, Khosravi A, Shoeibi A, Roshanzamir M, Moradnejad P, Nahavandi S, Khozeimeh F, Zare A, Panahiazar M, Acharya UR, Islam SMS. Risk factors prediction, clinical outcomes, and mortality in COVID-19 patients. J Med Virol. 2021 Apr;93(4):2307-2320. doi: 10.1002/jmv.26699. Epub 2020 Dec 17. PMID: 33247599; PMCID: PMC7753243. | | |
|  | Allen *et al.* 2020 | Mental health 🡪 risk and course |
| Allen B, El Shahawy O, Rogers ES, Hochman S, Khan MR, Krawczyk N. Association of substance use disorders and drug overdose with adverse COVID-19 outcomes in New York City: January-October 2020. J Public Health (Oxf). 2020 Dec 26:fdaa241. doi: 10.1093/pubmed/fdaa241. Epub ahead of print. PMID: 33367823; PMCID: PMC7799011. | | |
|  | Amin  *et al.* 2022 | Mental health 🡪 risk |
| Amin MM, Futrawan R, Husada MS. Correlation Between Schizophrenia and Coronavirus Disease in North Sumatera, Indonesia: A Correlative Analytical Study. Frontiers in psychiatry. 2022;13:896623–896623. | | |
|  | An *et al.* 2020 | Exclude, overlapping data |
| An, C., Lim, H., Kim, D.-W., Chang, J.H., Choi, Y.J., Kim, S.W., 2020. Machine learning prediction for mortality of patients diagnosed with COVID-19: a nationwide Korean cohort study. Scientific Reports 10 (1), 18716. | | |
|  | Ao *et al.* 2022 | Mental health 🡪 risk/outcome, mortality |
| Ao G, Li A, Wang Y, Li J, Tran C, Chen M, et al. Opioid usage and COVID-19 prognosis: A systematic review and meta-analysis. The American journal of emergency medicine. 2022;56:51–6. | | |
|  | Arbel *et al.* 2020 | No mental health 🡪 risk/outcome data |
| Arbel Y, Fialkoff C, Kerner A, Kerner M. (2020). Can Increased Recovery Rates from Coronavirus be explained  by Prevalence of ADHD? An Analysis at the US Statewide Level. *Journal of Attention* Disorders.  doi:10.1177/1087054720959707 | | |
|  | Arbel *et al.* 2021 | No mental health 🡪 risk and outcome |
| Arbel R, Hammerman A, Sergienko R, Friger M, Peretz A, Netzer D, Yaron S. BNT162b2 Vaccine Booster and  Mortality Due to Covid-19. N Engl J Med. 2021 Dec 23;385(26):2413-2420. doi: 10.1056/NEJMoa2115624.  Epub 2021 Dec 8. PMID: 34879190; PMCID: PMC8728797. | | |
|  | Arbello *et al.* 2020 | No mental health 🡪 risk/outcome data |
| Arbelo N, López-Pelayo H, Sagué M, Madero S, Pinzón-Espinosa J, Gomes-da-Costa S, Pintor L. (2021).  Psychiatric Clinical Profiles and Pharmacological Interactions in COVID-19 Inpatients Referred to a  Consultation Liaison Psychiatry Unit: a Cross-Sectional Study. *Psychiatric Quarterly*, 1-13.  doi:10.1007/s11126-020-09868-6 | | |
|  | Aswathy *et al.* 2022 | No mental health 🡪 risk and outcome |
| Raj SVA, Jacob A, Ambu V, Wilson T, Renuka R. Post COVID-19 clinical manifestations and its risk factors among patients in a Northern District in Kerala, India. J Family Med Prim Care. 2022 Sep;11(9):5312-5319. doi:10.4103/jfmpc.jfmpc_131_22. Epub 2022 Oct 14. PMID: 36505604; PMCID: PMC9731023. | | |
|  | Atkins *et al.* 2020 | Exclude, overlapping data |
| Atkins JL, Masoli JAH, Delgado J, Pilling LC, Kuo CL, Kuchel GA, Melzer, D. (2020). Preexisting Comorbidities  Predicting COVID-19 and Mortality in the UK Biobank Community Cohort. *Journals of Gerontology Series a-*  *Biological Sciences and Medical Sciences, 75*(11), 2224-2230. doi:10.1093/gerona/glaa183 | | |
|  | Attalla *et al.* 2021 | No mental health 🡪 risk/outcome data |
| Atalla E, Zhang RN, Shehadeh F, Mylona EK, Tsikala-Vafea M, Kalagara S, . . . Mylonakis E. (2021). Clinical  Presentation, Course, and Risk Factors Associated with Mortality in a Severe Outbreak of COVID-19 in Rhode  Island, USA, April-June 2020. *Pathogens, 10*(1). doi:10.3390/pathogens10010008 | | |
|  | Ausserhofer *et al.* 2023 | No mental health 🡪 risk/outcome data |
| Ausserhofer D, Mahlknecht A, Engl A, Piccoliori G, Pfitscher G, Silbernagl P, Giacomoni F, Pycha R, Lombardo S, Gärtner T, Mian M, Meier H, Wiedermann CJ, Keim R. Relationship between depression, anxiety, stress, and SARS-CoV-2 infection: a longitudinal study. Front Psychol. 2023 Apr 27;14:1116566. doi:  10.3389/fpsyg.2023.1116566. PMID: 37213392; PMCID: PMC10197902. | | |
|  | Ayana *et al.* 2021 | No mental health 🡪 risk/outcome data |
| Ayana GM, Merga BT, Birhanu A, Alemu A, Negash B, Dessie Y. Predictors of Mortality Among Hospitalized COVID-19 Patients at a Tertiary Care Hospital in Ethiopia. Infection and drug resistance. 2021;14:5363–73 | | |
|  | Azar *al.* 2020 | Mental health 🡪 risk and course |
| Azar KMJ, Shen Z, Romanelli RJ, Lockhart SH, Smits K, Robinson S, Brown S, Pressman AR.  Disparities In Outcomes Among COVID-19 Patients In A Large Health Care System In California.  Health Aff (Millwood). 2020 Jul;39(7):1253-1262. doi: 10.1377/hlthaff.2020.00598. Epub 2020  May 21. PMID: 32437224. | | |
|  | Bailey *al.* 2021 | Mental health 🡪 risk and course |
| Bailey, L. C., Razzaghi, H., Burrows, E. K., Bunnell, H. T., Camacho, P. E. F., Christakis, D. A., . . .  Forrest, C. B. (2021). Assessment of 135 794 Pediatric Patients Tested for Severe AcuteRespiratory  Syndrome Coronavirus 2 Across the United States. *JAMA Pediatr, 175*(2), 176-184.  doi:10.1001/jamapediatrics.2020.5052 | | |
|  | Baillargeon *et al.* 2021 | Mental health 🡪 course |
| Baillargeon, J., Polychronopoulou, E., Kuo, Y. F., & Raji, M. A. (2021). The Impact of Substance Use Disorder  on COVID-19 Outcomes. *Psychiatr Serv, 72*(5), 578-581. doi:10.1176/appi.ps.202000534 | | |
|  | Bain *et al.* 2021 | No mental health 🡪 risk/outcome data |
| Bain W, Yang H, Shah FA, Suber T, Drohan C, Al-Yousif N, DeSensi RS, Bensen N, Schaefer C, Rosborough BR, Somasundaram A, Workman CJ, Lampenfeld C, Cillo AR, Cardello C, Shan F, Bruno TC, Vignali DA, Ray P, Ray A, Zhang Y, Lee JS, Methé B, McVerry BJ, Morris A, Kitsios GD. COVID-19 versus Non-COVID ARDS: Comparison of Demographics, Physiologic Parameters, Inflammatory Biomarkers and Clinical Outcomes. Ann Am Thorac Soc. 2021 Feb 5. doi: 10.1513/AnnalsATS.202008-1026OC. Epub ahead of print. PMID: 33544045. | | |
|  | Bajaj *et al.* 2020 | No mental health 🡪 risk/outcome data |
| Bajaj JS, Solanki SL. Study of risk factors and psychological impact in physicians diagnosed with COVID-19: An online, postexposure, cross-sectional survey. J Anaesthesiol Clin Pharmacol. 2020 Jul-Sep;36(3):345-349. doi: 10.4103/joacp.JOACP_417_20. Epub 2020 Oct 16. PMID: 33487901; PMCID: PMC7812967. | | |
|  | Banoei *et al.* 2021 | No mental health 🡪 risk/outcome data |
| Banoei MM, Dinparastisaleh R, Zadeh AV, Mirsaeidi M. Machine-learning-based COVID-19 mortality prediction model and identification of patients at low and high risk of dying. Critical care (London, England). 2021;25(1):328–328. | | |
|  | Baranova *et al.* 2023 | Exclude because of overlap with controlled analyses [although strong because of Mendelian randomization [not included in the pre-registration] |
| Baranova A, Zhao Y, Cao H, Zhang F. Causal associations between major depressive disorder and COVID-19. Gen Psychiatr. 2023 Apr 5;36(2):e101006. doi:10.1136/gpsych-2022-101006. PMID: 37066117; PMCID: PMC10083530. | | |
|  | Barcella *et al.* 2020 | Mental health 🡪 risk and course |
| Barcella CA, Polcwiartek C, Mohr GH, Hodges G, Søndergaard K, Bang C, Andersen MP, Fosbøl E, Køber L, Schou M, Torp-Pedersen C, Kessing LV, Gislason G, Kragholm K. Severe mental illness is associated with increased mortality and severe course of COVID-19. Acta Psychiatr Scand. 2021 Apr 24. doi: 10.1111/acps.13309. Epub ahead of print. PMID: 33894064. | | |
|  | Barh *et al.* 2021 | No mental health 🡪 risk/outcome data |
| Barh, D., Tiwari, S., Andrade, B. S., Weener, M. E., Góes-Neto, A., Azevedo, V., . . . Ganguly, N. K. (2021). A  novel multi-omics-based highly accurate prediction of symptoms, comorbid conditions, and possible long-  term complications of COVID-19. *Mol Omics, 17*(2), 317-337. doi:10.1039/d0mo00189a | | |
|  | Basrak *et al.* 2021 | No comparison group for course |
| Basrak, N., Mulcrone, N., Sharifuddin, S., Ghumman, Z., Bechan, N., Mohamed, E., . . . Davoren, M. (2021).  Risk of adverse outcome of COVID-19 among patients in secure psychiatric services: observational cohort  study. *BJPsych Open, 7*(1), e31. doi:10.1192/bjo.2020.169 | | |
|  | Batty *et al.* 2020 | Exclude; double data |
| Batty, G. D., Deary, I. J., Luciano, M., Altschul, D. M., Kivimaki, M., & Gale, C. R. (2020). Psychosocial factors and hospitalisations for COVID-19: Prospective cohort study based on a community sample. *Brain Behavior and Immunity, 89*, 569-578. doi:10.1016/j.bbi.2020.06.021 | | |
|  | Batty *et al.* 2021 | Exclude; double data |
| Batty GD, Gale CR. Pre-pandemic mental illness and risk of mortality from COVID-19. Lancet Psychiatry. 2021 Mar;8(3):182-183. doi: 10.1016/S2215-0366(21)00002-X. PMID: 33610222; PMCID: PMC7906744 | | |
|  | Bayrak and Çadirci, 2021 | Mental health 🡪 course |
| Bayrak, M., & Çadirci, K. (2021). The associations of life quality, depression, and cognitive  impairment with mortality in older adults with COVID-19: A prospective, observational study. *Acta*  *Clinica Belgica*, 1-8. doi:10.1080/17843286.2021.1916687 | | |
|  | Becker *et al.* 2021 | No diagnosed mental health 🡪 risk/outcome data |
| Becker C, Beck K, Zumbrunn S, Memma V, Herzog N, Bissmann B, et al. Long COVID 1 year after hospitalization for COVID-19: a prospective bicentric cohort study. Swiss Medical Weekly. 2021;151:w30091–w30091. | | |
|  | Beckwith *et al.* 2022 | No mental health 🡪 risk/outcome data |
| Beckwith N, Probert J, Rosenbaum BL, Bains A, Angelucci VC, Morfin Rodriguez AE, et al. Demographic Features, Physical Examination Findings, and Medication Use in Hospitalized, Delirious Patients With and Without COVID-19 Infection: A Retrospective Study. Journal of the Academy of Consultation-Liaison Psychiatry. 2022. | | |
|  | Bellan *et al.* 2022 | Mental heath 🡪 Long covid |
| Bellan M, Apostolo D, Albè A, Crevola M, Errica N, Ratano G, Tonello S, Minisini R, D'Onghia D, Baricich A, Patrucco F, Zeppegno P, Gramaglia C, Balbo PE, Cappellano G, Casella S, Chiocchetti A, Clivati E, Giordano M, Manfredi M, Patti G, Pinato DJ, Puricelli C, Raineri D, Rolla R, Sainaghi PP, Pirisi M; No- More COVID study group. Determinants of long COVID among adults hospitalized for SARS-CoV-2 infection: A prospective cohort study. Front Immunol. 2022 Dec 19;13:1038227. doi: 10.3389/fimmu.2022.1038227. PMID: 36601115; PMCID:PMC9807078. | | |
|  | Benzano *et al.* 2021 | No mental health 🡪 risk/outcome data |
| Benzano D, Ornell F, Schuch JB, Pechansky F, Sordi AO, von Diemen L, Kessler FH. (2021). Clinical  vulnerability for severity and mortality by COVID-19 among users of alcohol and other substances. *Psychiatry*  *Res, 300*, 113915. doi:10.1016/j.psychres.2021.113915 | | |
|  | Bhargava *et al.* 2021 | No mental health 🡪 risk/outcome data |
| Bhargava A, Sharma M, Riederer K, Fukushima EA, Szpunar SM, Saravolatz L. Risk Factors for In-hospital Mortality from Coronavirus Disease 2019 Infection Among Black Patients—An Urban Center Experience. Clinical infectious diseases. 2021;73(11):e4005–e4011. | | |
|  | Bhopalwala *et al.* 2022 | Mental health 🡪 outcome |
| Bhopalwala H, Dewaswala N, Kolagatla S, Wisnieski L, Piercy J, Bhopalwala A, et al. Predictors of Mortality for Patients with COVID-19 in the Rural Appalachian Region. International journal of general medicine. 2022;15:2207–14. | | |
|  | Boland *et al.* 2020 | No mental health 🡪 risk/outcome data |
| Boland X, Dratcu L. Clozapine in the Time of COVID-19. Clin Psychopharmacol Neurosci. 2020 Aug 31;18(3):450-453. doi: 10.9758/cpn.2020.18.3.450. PMID: 32702224; PMCID: PMC7383003. | | |
|  | Breslau *et al.* 2021 | No mental health 🡪 risk/outcome data |
| Breslau J, Finucane ML, Locker AR, Baird MD, Roth EA, Collins RL. A longitudinal study of psychological distress in the United States before and during the COVID-19 pandemic. Prev Med. 2021 Feb;143:106362. doi: 10.1016/j.ypmed.2020.106362. Epub 2020 Dec 31. PMID: 33388325. | | |
|  | Brieghel *et al.* 2021 | Exclude; double data |
| Brieghel C, Ellekvist P, Lund ML, Soborg C, Walsted ES, Thomsen JJ, . . . Ravn P. (2021). Prognostic factors  of 90-day mortality in patients hospitalised with COVID-19. *Dan Med J, 68*(3). | | |
|  | Brinkman *et al.* 2022 | No mental health 🡪 risk/outcome data |
| Brinkman S, Termorshuizen F, Dongelmans DA, Bakhshi-Raiez F, Arbous MS, de Lange DW, et al. Comparison of outcome and characteristics between 6343 COVID-19 patients and 2256 other community-acquired viral pneumonia patients admitted to Dutch ICUs. Journal of critical care. 2022;68:76–82. | | |
|  | Bruggmann *et al.* 2022 | No Mental health 🡪 risk/outcome |
| Bruggmann P, Senn O, Frei A, Puhan MA, Fehr J, Falcato L. High SARS-CoV-2 seroprevalence but no severe course of COVID-19 disease among people on opioid agonist treatment in Zurich: a cross-sectional study. Swiss Medical Weekly. 2022;152:w30122–w30122. | | |
|  | Bucholc *et al.* 2022 |  |
| Bucholc M, Bradley D, Bennett D, Patterson L, Spiers R, Gibson D, Van Woerden H, Bjourson AJ. Identifying pre-existing conditions and multimorbidity patterns associated with in-hospital mortality in patients with COVID-19. Sci Rep. 2022 Oct 15;12(1):17313. doi: 10.1038/s41598-022-20176-w. PMID: 36243878; PMCID:PMC9568958. | | |
|  | Buonsenso *et al.* 2022 | No mental health 🡪 risk/outcome data |
| Buonsenso D, Pujol FE, Munblit D, Pata D, McFarland S, Simpson FK. Clinical characteristics, activity levels and mental health problems in children with long coronavirus disease: a survey of 510 children. Future microbiology. 2022;17(8):577–88. | | |
|  | Burgaña *et al.* 2021 | No mental health 🡪 risk/outcome data |
| Burgaña Agoües A, Serra Gallego M, Hernández Resa R, Joven Llorente B, Lloret Arabi M, Ortiz Rodriguez J, et al. Risk Factors for COVID-19 Morbidity and Mortality in Institutionalised Elderly People. International journal of environmental research and public health. 2021;18(19):10221. | | |
|  | Bushman *et al.* 2021 | Mental health 🡪 mortality |
| Bushman D, Davidson A, Pathela P, Greene SK, Weiss D, Reddy V, et al. Risk Factors for Mortality Among Hospitalized Patients Aged 21–64 Years Diagnosed with COVID-19—New York City, March 13–April 9, 2020. Journal of racial and ethnic health disparities. 2021;9(4):1584–99. | | |
|  | Buttiron *et al.* 2021 | No mental health 🡪 risk/outcome data |
| Buttiron Webber T, Provinciali N, Briata IM, Boitano M, Defferrari C, Magnani M, et al. Predictors of poor seroconversion and adverse events to SARS-CoV-2 mRNA BNT162b2 vaccine in cancer patients on active treatment. Role of the Research Nurse. In: Professioni infermieristiche. Italy; 2021. p. 261–261. | | |
|  | Canal-Rivero *et al.* 2021 | Mental health 🡪 risk and course |
| Canal-Rivero M, Catalán-Barragán R, Rubio-García A, Garrido-Torres N, Crespo- Facorro B, Ruiz-Veguilla M;  IBIS Translational Psychiatry Group. Lower risk of SARS-CoV2 infection in individuals with severe mental  disorders on antipsychotic treatment: A retrospective epidemiological study in a representative Spanish  population. Schizophr Res. 2021 Mar;229:53-54. doi: 10.1016/j.schres.2021.02.002. Epub 2021 Feb 19.  PMID: 33631466; PMCID: PMC7894093. | | |
|  | Canal-Rivero *et al.* 2023 | No usable data |
| Canal-Rivero M, Vázquez-Hernández J, León-Gómez M, Maraver-Ayala S, Fernández-Portes L, Sánhez-Benítez S, Garrido-Torres N, Ruiz-Veguilla M, Crespo-Facorro B. Epidemiology of infection, transmission and COVID-19 outcomes among mental health users and workers in a comprehensive network of long-term mental health facilities: Retrospective observational population-base study. Schizophr Res. 2023 Apr;254:1-7. doi: 10.1016/j.schres.2023.01.020. Epub 2023 Jan 20. PMID: 36736100; PMCID: PMC9852313. | | |
|  | Cao *et al.* 2020 *BMJ Open* | No mental health 🡪 risk/outcome data |
| Cao Y, Hiyoshi A, Montgomery S. COVID-19 case-fatality rate and demographic and socioeconomic influencers: worldwide spatial regression analysis based on country-level data. BMJ Open. 2020 Nov 3;10(11):e043560. doi: 10.1136/bmjopen-2020-043560. PMID: 33148769; PMCID: PMC7640588 | | |
|  | Cao *et al.* 2020 *PLoS ONE* | No mental health 🡪 risk/outcome data |
| Cao Z, Li T, Liang L, Wang H, Wei F, Meng S, Cai M, Zhang Y, Xu H, Zhang J, Jin R. Clinical characteristics of Coronavirus Disease 2019 patients in Beijing, China. PLoS One. 2020 Jun 17;15(6):e0234764. doi: 10.1371/journal.pone.0234764. PMID: 32555674; PMCID: PMC7299347. | | |
|  | Carey *et al.* 2021 | No mental health 🡪 risk/outcome data |
| Carey IM, Cook DG, Harris T, DeWilde S, Chaudhry UAR, Strachan DP. Risk factors for excess all-cause mortality during the first wave of the COVID-19 pandemic in England: A retrospective cohort study of primary care data. PloS one. 2021;16(12):e0260381–e0260381. | | |
|  | Carrat *et al.* 2021 | No mental health 🡪 risk/outcome data |
| Carrat F, Touvier M, Severi G, Meyer L, Jusot F, Lapidus N, Rahib D, Lydié N, Charles MA, Ancel PY, Rouquette A, de Lamballerie X, Zins M, Bajos N; SAPRIS study group. Incidence and risk factors of COVID-19-like symptoms in the French general population during the lockdown period: a multi-cohort study. BMC Infect Dis. 2021 Feb 10;21(1):169. doi: 10.1186/s12879-021-05864-8. PMID: 33568097; PMCID: PMC7875161. | | |
|  | Caspersen *et al.* 2022 | No mental health 🡪 risk/outcome data |
| Caspersen IH, Magnus P, Trogstad L. Excess risk and clusters of symptoms after COVID-19 in a large Norwegian cohort. European journal of epidemiology. 2022;37(5):539–48. | | |
|  | Castro *et al.* 2021 | Mental health 🡪 course |
| Castro VM, Gunning FM, McCoy TH, Perlis RH. Mood Disorders and Outcomes of COVID-19 Hospitalizations. Am J Psychiatry. 2021 Apr 6:appiajp202020060842. doi: 10.1176/appi.ajp.2020.20060842. Epub ahead of print. PMID: 33820425. | | |
|  | Catalan *et al.* 2022 | Mental health 🡪 risk |
| Catalan A, Aymerich C, Bilbao A, Pedruzo B, Pérez JL, Aranguren N, Salazar de Pablo G, Hedges E, Gil P, Segarra R, González-Pinto A, Fernández-Rivas A, Inchausti L, McGuire P, Fusar-Poli P, González-Torres MÁ. Psychosis and substance abuse increase the COVID-19 mortality risk. Psychol Med. 2022 Apr12;53(9): 1-9. doi: 10.1017/S0033291722000976. Epub ahead of print. PMID: 35410632; PMCID: PMC9114752. | | |
|  | Cavallaro *et al.* 2021 | Mental health 🡪 risk and course |
| Cavallaro M, Moiz H, Keeling MJ, McCarthy ND. Contrasting factors associated with COVID-19-related ICU  admission and mortality outcomes in hospitalised patients by means of Shapley values. PLoS Comput Biol. 2021  Jun 23;17(6):e1009121. doi: 10.1371/journal.pcbi.1009121. PMID: 34161326; PMCID: PMC8259985. | | |
|  | Ceban *et al.* 2021 | Meta-analysis |
| Ceban F, Nogo D, Carvalho IP, Lee Y, Nasri F, Xiong J, et al. Association Between Mood Disorders and Risk of COVID-19 Infection, Hospitalization, and Mortality: A Systematic Review and Meta-analysis. Archives of general psychiatry. 2021;78(10):1079–91. | | |
|  | Chang *et al.* 2021 | Mental health 🡪 course |
| Chang MH, Moonesinghe R, Truman BI. COVID-19 Hospitalization by Race and Ethnicity: Association with  Chronic Conditions Among Medicare Beneficiaries. J Racial Ethn Health Disparities. 2021 Jan 8:1–10. doi:  10.1007/s40615-020-00960-y. Epub ahead of print. PMID: 33420609; PMCID: PMC7793388. | | |
|  | Chaudhary *et al.* 2022 | No mental health 🡪 risk/outcome data |
| Chaudhary A, Singh UN, Paudel P, Thapa N, Khadka K, Sah PK, et al. Characteristics and outcomes of hospitalized adults with COVID-19 in Nepal: a multicenter, prospective cohort study. Journal of infection in developing countries. 2022;16(3):469–77. | | |
|  | Chen *et al.* 2021a | Mental health 🡪 mortality |
| Chen S, Fernandez-Egea E, Jones PB, Lewis JR, Cardinal RN. Longer-term mortality following SARS-CoV-2 infection in people with severe mental illness: retrospective case-matched study. BJPsych open. 2021;7(6):e201–e201. | | |
|  | Chen *et al.* 2021b | Mental health 🡪 mortality |
| Chen S, Jones PB, Underwood BR, Fernandez‐Egea E, Qin P, Lewis JR, et al. Risk factors for excess mortalitys during lockdown among older users of secondary care mental health services without confirmed COVID‐19: A retrospective cohort study. International journal of geriatric psychiatry. 2021;36(12):1899–907. | | |
|  | Cho *et al.* 2021 | No mental health 🡪 risk/outcome data |
| Cho SY, Park SS, Song MK, Bae YY, Lee DG, Kim D. W. (2021). Prognosis Score System to Predict Survival for COVID-19 Cases: a Korean Nationwide Cohort Study. *J Med Internet Res, 23*(2). doi:10.2196/26257 | | |
|  | Chung *et al.* 2021 | No mental health 🡪 risk/outcome data |
| Chung F, Waseem R, Pham C, Penzel T, Han F, Bjorvatn B, ... Partinen M. (2021). The association between  high risk of sleep apnea, comorbidities, and risk of COVID-19: a population-based international harmonized  study. *Sleep and Breathing*, 1-12. doi:10.1007/s11325-021-02373-5 | | |
|  | Ch’en *et al.* 2023 | No mental health 🡪 risk/outcome data |
| Ch'en PY, Gold LS, Lu Q, Ye T, Andrews JS, Patel P. Exploring risk factors for persistent neurocognitive sequelae after hospitalization for COVID-19. Ann Clin Transl Neurol. 2023 Jun 2. doi: 10.1002/acn3.51801. Epub ahead of print. PMID: 37265172. | | |
|  | Clift *et al.* 2020 | Mental health 🡪 course; suspected double data and no inclusion for the course analyses (see study overlap in the online supplement) |
| Clift AK, Coupland CAC, Keogh RH, et al. Living risk prediction algorithm (QCOVID) for risk of hospital admission and mortality from coronavirus 19 in adults: national derivation and validation cohort study. *BMJ* 2020; 371: m3731. | | |
|  | Clouston *et al.* 2021 | Mental health 🡪 mortality |
| Clouston SAP, Luft BJ, Sun E. Clinical risk factors for mortality in an analysis of 1375 patients admitted for COVID treatment. Scientific reports. 2021;11(1):23414–23414. | | |
|  | Cohen *et al.* 2022 | Mental health 🡪 risk/outcome |
| Cohen HA, Gerstein M, Yaniv N, Richenberg Y, Jacobson E, Marton S, et al. Attention-Deficit/Hyperactivity Disorder as a Risk Factor for COVID-19 Infection. Journal of attention disorders. 2022;26(7):985–90. | | |
|  | Cosco *et al.* 2021 | No mental health 🡪 risk/outcome data |
| Cosco TD, Fortuna K, Wister A, Riadi I, Wagner K, Sixsmith A. COVID-19, Social Isolation, and Mental Health Among Older Adults: A Digital Catch-22. J Med Internet Res. 2021 May 6;23(5):e21864. doi: 10.2196/21864. PMID: 33891557; PMCID: PMC8104002. | | |
|  | Crook et al. 2021 | Review |
| Crook H, Raza S, Nowell J, Young M, Edison P. Long covid—mechanisms, risk factors, and management. BMJ (Online). 2021;374:n1648–n1648. | | |
|  | Cummins *et al.* 2021 | Mental health 🡪 risk and course |
| Cummins L, Ebyarimpa I, Cheetham N, Tzortziou Brown V, Brennan K, Panovska‐Griffiths J. (2021). Factors associated with COVID‐19 related hospitalisation, critical care admission and mortality using linked primary and secondary care data. *Influenza and Other Respiratory Viruses*. | | |
|  | Dai *et al.* 2021 | No mental health 🡪 risk/outcome data |
| Dai M, Tao L, Chen Z, Tian Z, Guo X, Allen-Gipson DS, Tan R, Li R, Chai L, Ai F, Liu M. Influence of Cigarettes and Alcohol on the Severity and Mortality of COVID-19: A Multicenter Retrospective Study in Wuhan, China. Front Physiol. 2020 Dec 9;11:588553. doi: 10.3389/fphys.2020.588553. PMID: 33362576; PMCID: PMC7756110. | | |
|  | Dai *et al.* 2022 | Mental health 🡪 risk/outcome |
| Dai XJ, Shao Y, Ren L, Tao W and Wang Y (2022). Risk factors of COVID-19 in subjects with and without mental disorders. Journal of Affective Disorders. 2022 Jan 15;297:102-111. doi: 10.1016/j.jad.2021.10.024 | | |
|  | Daines *et al.* 2023 | No mental health 🡪 risk/outcome |
| Daines L, Zheng B, Elneima O, Harrison E, Lone NI, Hurst JR, Brown JS, Sapey E, Chalmers JD, Quint JK, Pfeffer P, Siddiqui S, Walker S, Poinasamy K, McAuley H, Sereno M, Shikotra A, Singapuri A, Docherty AB, Marks M, Toshner M, Howard LS, Horsley A, Jenkins G, Porter JC, Ho LP, Raman B, Wain LV, Brightling CE,  Evans RA, Heaney LG, De Soyza A, Sheikh A. Characteristics and risk factors for post-COVID-19 breathlessness after hospitalisation for COVID-19. ERJ Open Res. 2023 Feb 20;9(1):00274-2022. doi: 10.1183/23120541.00274-2022. PMID: 36820079; PMCID: PMC9790090. | | |
|  | Damerdji *et al.* 2022 | No mental health 🡪 risk/outcome data |
| Damerdji F, Rotsaert M, Wacquier B, Hein M, Loas G. Prevalence and Relationships between Alexithymia, Anhedonia, Depression and Anxiety during the Belgian COVID-19 Pandemic Lockdown. Int J Environ Res Public Health. 2022 Nov18;19(22):15264. doi: 10.3390/ijerph192215264. PMID: 36430003; PMCID:  PMC9691107. | | |
|  | Dang *et al.* 2022 | No mental health 🡪 risk/outcome data |
| Dang LT, Luong TC, Nguyen DH, Hoang TA, Nguyen HT, Nguyen HC, et al. The Associations of Suspected COVID-19 Symptoms with Anxiety and Depression as Modified by Hemodialysis Dietary Knowledge: A Multi-Dialysis Center Study. Nutrients. 2022;14(12):2364. | | |
|  | Das *et al.* 2021 | No mental health 🡪 risk/outcome data |
| Das A, Halder A, Patil RS, Harshe DG (2021). Susceptibility of clinically depressed patients to OVID-19: Is  there a link? *Indian J Psychiatry, 63*(1), 112-113. doi: 10.4103/psychiatry. IndianJPsychiatry_850_20 | | |
|  | De Girolama *et al.* 2021 | No mental health 🡪 risk/outcome data |
| de Girolamo G, Bellelli G, Bianchetti A, Starace F, Zanetti O, Zarbo C, Micciolo R. Older People Living in Long-Term Care Facilities and Mortality Rates During the COVID-19 Pandemic in Italy: Preliminary Epidemiological Data and Lessons to Learn. Front Psychiatry. 2020 Oct 14;11:586524. doi: 10.3389/fpsyt.2020.586524. PMID: 33173526; PMCID: PMC7591767. | | |
|  | de Leon *et al.* 2020 | Opinion paper |
| de Leon J, Ruan CJ, Verdoux H, Wang C. Clozapine is strongly associated with the risk of pneumonia and inflammation. Gen Psychiatr. 2020 Apr 16;33(2):e100183. doi: 10.1136/gpsych-2019-100183. PMID: 32420521; PMCID: PMC7199914. | | |
|  | Descamps *et al.* 2022 | Mental health 🡪 outcome |
| Descamps A, Frenkiel J, Zarca K, Laidi C, Godin O, Launay O, Leboyer M, Durand-Zaleski I. Association between mental disorders and COVID-19 outcomes among inpatients in France: A retrospective nationwide population-based study. J Psychiatr Res. 2022 Nov;155:194-201. doi: 10.1016/j.jpsychires.2022.08.019. Epub 2022 Aug 20. PMID: 36063611; PMCID: PMC9392549. | | |
|  | Delora *et al.* 2022 | Exposure variable unknown |
| Delora A, Mills A, Jacobson D, Cornett B, Peacock WF, Datta A, Jenks SP. Socioeconomic and Comorbid Factors Affecting Mortality and Length of Stay in COVID-19 Patients. Cureus. 2022 Oct 12;14(10):e30224. doi: 10.7759/cureus.30224. PMID: 36381875; PMCID: PMC9651930. | | |
|  | de Miranda *et al.* 2022 | Mental health 🡪 long covid |
| de Miranda DAP, Gomes SVC, Filgueiras PS, Corsini CA, Almeida NBF, Silva RA, Medeiros MIVARC, Vilela RVR, Fernandes GR and Grenfell RFQ (2022). Long COVID-19 syndrome: a 14-months longitudinal study during the two first epidemic peaks in Southeast Brazil. Transactions of the Royal Society of Tropical Medicine and Hygiene. 2022 Nov 1;116(11):1007-1014. doi: 10.1093/trstmh/trac030. | | |
|  | de Picker *et al.* 2021 | No mental health 🡪 risk/outcome data |
| De Picker LJ, Dias MC, Benros ME, Vai B, Branchi I, Benedetti F, Borsini A, Leza JC, Kärkkäinen H, Männikkö M, Pariante CM, Güngör ES, Szczegielniak A, Tamouza R, van der Markt A, Fusar-Poli P, Beezhold J, Leboyer M. Severe mental illness and European COVID-19 vaccination strategies. Lancet Psychiatry. 2021 May;8(5):356-359. doi: 10.1016/S2215-0366(21)00046-8. Epub 2021 Feb 17. | | |
|  | de Vito *et al.* 2021 [MEDRXIV] | Mental health 🡪 risk and course |
| De Vito A, Fiore V, Princic E, Geremia N, Napodano CMP, Muredda AA, . . . Babudieri S. (2021). Predictors of  infection, symptoms development, and mortality in people with SARS-CoV-2 living in retirement nursing  homes. *PLoS One, 16*(3). doi:10.1371/journal.pone.0248009 | | |
|  | Diaz *et al.* 2021 | No mental health 🡪 risk/outcome data |
| Diaz A, Baweja R, Bonatakis JK, Baweja R. Global health disparities in vulnerable populations of psychiatric patients during the COVID-19 pandemic. World J Psychiatry. 2021 Apr 19;11(4):94-108. doi: 10.5498/wjp.v11.i4.94. PMID: 33889535; PMCID: PMC8040151. | | |
|  | Díaz-Simón *et al.* 2021 | Mental health 🡪 risk/outcome- alcohol abuse |
| Díaz-Simón R, Lalueza A, Lora-Tamayo J, Rubio-Rivas M, Mendo CL, Martínez MLT, et al. Clinical Characteristics and Risk Factors of Respiratory Failure in a Cohort of Young Patients Requiring Hospital Admission with SARS-CoV2 Infection in Spain: Results of the Multicenter SEMI-COVID-19 Registry. Journal of general internal medicine : JGIM. 2021;36(10):3080–7. | | |
|  | Diez-Quevedo *et al.* 2021 | Mental health 🡪 course |
| Diez-Quevedo C, Iglesias-Gonzalez M, Giralt-Lopez M, Rangil T, Sanagustin D, Moreira M, . . . Cuevas-Esteban  J. (2021). Mental disorders, psychopharmacological treatments, and mortality in 2150 COVID-19 Spanish  inpatients. *Acta Psychiatr Scand, 143*(6), 526-534. doi:10.1111/acps.13304 | | |
|  | Diminich *et al.* 2022 | Only poster abstract is available |
| Diminich E, Clouston S, Luft B. P271. Risk Factors Associated With SARS-CoV-2 Infection and COVID-19 Related Depression Among Immigrant Latino Essential Workers in Suffolk County, New York. Biological psychiatry (1969). 2022;91(9):S197. | | |
|  | Djuric *et al.* 2021 | No mental health 🡪 risk/outcome data |
| Djuric O, Mancuso P, Zannini A, Nicolaci A, Massari M, Zerbini A, Belloni L, Collini G, Sampaolesi F, Celotti A, Boni I, Giorgi Rossi P; Reggio Emilia COVID-19 Working Group. Are Individuals with Substance Use Disorders at Higher Risk of SARS-CoV-2 Infection? Population-Based Registry Study in Northern Italy. Eur Addict Res. 2021 May 5:1-5. doi: 10.1159/000515101. Epub ahead of print. PMID: 33951659. | | |
|  | Dobre *et al.* 2021 | No mental health 🡪 risk/outcome data |
| Dobre D, Schwan R, Jansen C, Schwitzer T, Martin O, Ligier F, . . . Laprevote V. (2021). Clinical features and  outcomes of COVID-19 patients hospitalized for psychiatric disorders: a French multi-centered prospective  observational study. *Psychol Med*, 1-9. doi:10.1017/s0033291721001537 | | |
|  | Douville *et al.* 2021 | Mental health 🡪 course: but outcome variable entails > 1 category |
| Douville NJ, Douville CB, Mentz G, Mathis MR, Pancaro C, Tremper KK, Engoren M. Clinically  applicable approach for predicting mechanical ventilation in patients with COVID-19. Br J Anaesth.  2021 Mar;126(3):578-589. doi: 10.1016/j.bja.2020.11.034. Epub 2020 Dec 4. PMID: 33454051;  PMCID: PMC7833820. | | |
|  | Durstenfeld *et al.* 2023 | Mental health 🡪 Longcovid data |
| Durstenfeld MS, Peluso MJ, Peyser ND, Lin F, Knight SJ, Djibo A, Khatib R, Kitzman H, O'Brien E, Williams N, Isasi C, Kornak J, Carton TW, Olgin JE, Pletcher MJ, Marcus GM, Beatty AL. Factors Associated With Long COVID Symptoms in an Online Cohort Study. Open Forum Infect Dis. 2023 Feb 1;10(2):ofad047. doi:  10.1093/ofid/ofad047. PMID: 36846611; PMCID: PMC9945931. | | |
|  | Egede *et al.* 2021 | Mental health 🡪 risk and course |
| Egede C, Dawson AZ, Walker RJ, Garacci E, Campbell JA, Egede LE. (2021). Relationship between mental  health diagnoses and COVID-19 test positivity, hospitalization, and mortality in Southeast Wisconsin. *Psychol*  *Med*, 1-9. doi:10.1017/s0033291721002312 | | |
|  | Ekinci *et al.* 2021 | No mental health 🡪 risk/outcome data |
| Ekinci O, Ekinci AE. Considering the prevalence and protective factors of COVID-19 in patients with schizophrenia. Encephale. 2021 Mar 11:S0013-7006(21)00054-3. doi: 10.1016/j.encep.2021.01.001. Epub ahead of print. PMID: 33814165; PMCID: PMC7951799. | | |
|  | Englett *et al.* 2021 | Case report / opinion |
| Englett B, Magdalany A, Gordon TL, Holladay K. COVID-19 reinfection in a patient with a serious mental illness within a long-term inpatient psychiatric care hospital. The mental health clinician. 2021;11(5):292–6. | | |
|  | Essau *et al.* 2021 | No mental health 🡪 risk/outcome data |
| Essau CA, de la Torre-Luque A. Adolescent psychopathological profiles and the outcome of the COVID-19 pandemic: Longitudinal findings from the UK Millennium Cohort Study. Progress in neuro-psychopharmacology & biological psychiatry. 2021;110:110330–110330. | | |
|  | Fabelo-Roche *et al.* 2021 | No mental health 🡪 risk/outcome data |
| Fabelo-Roche JR, Iglesias-Moré S, Gómez-García AM. Persons with Substance Abuse Disorders and Other Addictions: Coping with the COVID-19 Pandemic. MEDICC Rev. 2021 Apr;23(2):10. doi: 10.37757/MR2021.V23.N2.2. Epub 2021 Apr 14. PMID: 33974609. | | |
|  | Fan *et al.* 2021 | No mental health 🡪 risk/outcome data |
| Fan X, Liu Z, Poulsen KL, Wu X, Miyata T, Dasarathy S, Rotroff DM, Nagy LE. Alcohol Consumption is Associated with Poor Prognosis in Obese Patients with COVID-19: a Mendelian Randomization Study using UK Biobank. medRxiv [Preprint]. 2020 Nov 30:2020.11.25.20238915. doi: 10.1101/2020.11.25.20238915. PMID: 33269370; PMCID: PMC7709191. | | |
|  | Fares-Otero *et al.* 2021 | No mental health 🡪 risk/outcome data |
| Fares-Otero NE, Trautmann S, Pfaltz MC, Rodriguez-Jimenez R. Targeting adverse stress-related consequences of the COVID-19 crisis in individuals with psychotic disorders and childhood maltreatment. J Psychiatr Res. 2021 Apr 30;138:453-455. doi: 10.1016/j.jpsychires.2021.04.031. Epub ahead of print. PMID: 33964683. | | |
|  | Farrar *et al.* 2022 | No mental health 🡪 risk/outcome data |
| Farrar DS, Drouin O, Moore Hepburn C, Baerg K, Chan K, Cyr C, Donner EJ, Embree JE, Farrell C, Forgie S, Giroux R, Kang KT, King M, Laffin Thibodeau M, Orkin J, Ouldali N, Papenburg J, Pound CM, Price VE, Proulx-Gauthier JP, Purewal R, Ricci C, Sadarangani M, Salvadori MI, Thibeault R, Top KA, Viel-Thériault I,  Kakkar F, Morris SK. Risk factors for severe COVID-19 in hospitalized children in Canada: A national prospective study from March 2020-May 2021. Lancet Reg Health Am. 2022 Nov;15:100337. doi: 10.1016/j.lana.2022.100337. Epub 2022 Aug 1. PMID: 35936225; PMCID: PMC9342862. | | |
|  | Fitriawan *et al.* 2023 | No mental health 🡪 risk/outcome data |
| Fitriawan AS, Setyaningsih WAW, Wulandari AN, Samutri E, Achmad BF, Budiyati GA, Retnaningsih LN. (2023). Prevalence and predictors of suicidality among nursing students in Indonesia. *KONTAKT-Journal of Nursing & Social Sciences related to Health & Illness*, *25*(1). | | |
|  | Fitzgerald *et al.* 2021 | No mental health 🡪 risk/outcome data |
| Fitzgerald KC, Mecoli CA, Douglas M, Harris S, Aravidis B, Albayda J, Sotirchos ES, Hoke A, Orbai AM, Petri M, Christopher-Stine L, Baer AN, Paik JJ, Adler BL, Tiniakou E, Timlin H, Bhargava P, Newsome SD, Venkatesan A, Chaudhry V, Lloyd TE, Pardo CA, Stern BJ, Lazarev M, Truta B, Saidha S, Chen ES, Sharp M, Gilotra N, Kasper EK, Gelber AC, Bingham CO 3rd, Shah AA, Mowry EM. Risk factors for infection and health impacts of the covid-19 pandemic in people with autoimmune diseases. medRxiv [Preprint]. 2021 Feb 5:2021.02.03.21251069. doi: 10.1101/2021.02.03.21251069. Clin Infect Dis. 2021 May 06;: PMID: 33564774; PMCID: PMC7872366. | | |
|  | Fond *et al.* 2021 | Mental health 🡪 course and mortality |
| Fond G, Pauly V, Leone M, Llorca PM, Orleans V, Loundou A, Lancon C, Auquier P, Baumstarck K, Boyer L. Disparities in Intensive Care Unit Admission and Mortality Among Patients With Schizophrenia and COVID-19: A National Cohort Study. Schizophr Bull. 2021 Apr 29;47(3):624-634. doi: 10.1093/schbul/sbaa158. PMID: 33089862; PMCID: PMC7665717. | | |
|  | Fond *et al.* 2021 | Not available |
| Fond G, Pauly V, Orleans V, Antonini F, Fabre C, Sanz M, Klay S, Jimeno MT, Leone M, Lancon C, Auquier P, Boyer L. Increased in-hospital mortality from COVID-19 in patients with schizophrenia. Encephale. 2021 Apr;47(2):89-95. doi: 10.1016/j.encep.2020.07.003. Epub 2020 Jul 30. PMID: 32933762; PMCID: PMC7392112. | | |
|  | Francis *et al.* 2021 | Mental health 🡪 course |
| Francis NA, Stuart B, Knight M, Vancheeswaran R, Oliver C, Willcox M, Barlow A, Moore M. Predictors of clinical deterioration in patients with suspected COVID-19 managed in a 'virtual hospital' setting: a cohort study. BMJ Open. 2021 Mar 23;11(3):e045356. doi: 10.1136/bmjopen-2020-045356. PMID: 33757955; PMCID: PMC7992373. | | |
|  | Fuld *et al.* 2022 | No preexisting mental health 🡪 risk/outcome data |
| Fuld J, Jenkins RG, Man WDC, Openshaw PJM, Scott JT, Quint J, et al. Clinical characteristics with inflammation profiling of long COVID and association with 1-year recovery following hospitalisation in the UK: a prospective observational study. The lancet respiratory medicine. 2022;10(8):761–75. | | |
|  | Fumagalli *et al.* 2022 | No mental health 🡪 risk/outcome data |
| Fumagalli C, Zocchi C, Tassetti L, Silverii MV, Amato C, Livi L, et al. Factors associated with persistence of symptoms 1 year after COVID-19: A longitudinal, prospective phone-based interview follow-up cohort study. European journal of internal medicine. 2022;97:36–41. | | |
|  | Gabunia *et al.* 2022 | No mental health 🡪 risk/outcome data |
| Gabunia S, Harmon EY, Sonagere MB, Teale AE. Characteristics and Outcomes of COVID-19 Survivors Requiring Inpatient Rehabilitation: A Comparison of Two Waves. Am J Phys Med Rehabil. 2023 Mar 1;102(3):206-213. doi:10.1097/PHM.0000000000002059. Epub 2022 Jun 27. PMID: 35762847; PMCID:  PMC9940787. | | |
|  | Gang *et al.* 2022 | No mental health 🡪 risk/outcome data |
| Gang J, Falzarano F, She WJ, Winoker H, Prigerson HG. Are mortalitys from COVID-19 associated with higher rates of prolonged grief disorder (PGD) than mortalitys from other causes? Mortality studies. 2022;46(6):1287–96. | | |
|  | Gao *et al.* 2020 | No mental health 🡪 risk/outcome data |
| Gao C, Zhao Z, Li F, Liu JL, Xu H, Zeng Y, Yang L, Chen J, Lu X, Wang C, Guo Q. The impact of individual lifestyle and status on the acquisition of COVID-19: A case-Control study. PLoS One. 2020 Nov 5;15(11):e0241540. doi: 10.1371/journal.pone.0241540. PMID: 33152004; PMCID: PMC7643946. | | |
|  | García-Cabrera *et al.* 2021 | Mental health 🡪 mortality |
| García-Cabrera L, Pérez-Abascal N, Montero-Errasquín B, Rexach Cano L, Mateos-Nozal J, Cruz-Jentoft A. Characteristics, hospital referrals and 60-day mortality of older patients living in nursing homes with COVID-19 assessed by a liaison geriatric team during the first wave: a research article. BMC geriatrics. 2021;21(1):1–610. | | |
|  | Gasnier *et al.* 2022 | Mental health 🡪 risk/outcome; idlongcov |
| Gasnier M, Choucha W, Radiguer F, Faulet T, Chappell K, Bougarel A, Kondarjian C, Thorey P, Baldacci A, Ballerini M, Ait Tayeb AEK, Herrero H, Hardy-Leger I, Meyrignac O, Morin L, Lecoq AL, Pham T, Noel N, Jollant F, Montani D, Monnet X, Becquemont L, Corruble E, Colle R; COMEBAC study group. Comorbidity of long COVID and psychiatric disorders after a hospitalisation for COVID-19: a cross-sectional study. J Neurol Neurosurg Psychiatry. 2022 Aug 11:jnnp-2021-328516. doi: 10.1136/jnnp-2021-328516. Epub ahead of print. PMID: 35953265. | | |
|  | Gaspar *et al.* 2023 | No mental health 🡪 risk/outcome data |
| Gaspar P, Dias M, Parreira I, Gonçalves HD, Parlato F, Maione V, Atalaia Barbacena H, Carreiro C, Duarte L. Predictors of Long-COVID-19 and its Impact on Quality of Life: Longitudinal Analysis at 3, 6 and 9 Months after Discharge from a Portuguese Centre. Acta Med Port. 2023 Feb 24. doi: 10.20344/amp.19047. Epub  ahead of print. PMID: 36827994. | | |
|  | Gayam *et al.* 2020 | No mental health 🡪 risk/outcome data |
| Gayam V, Konala VM, Naramala S, Garlapati PR, Merghani MA, Regmi N, Balla M, Adapa S. Presenting characteristics, comorbidities, and outcomes of patients coinfected with COVID-19 and Mycoplasma pneumoniae in the USA. J Med Virol. 2020 Oct;92(10):2181-2187. doi: 10.1002/jmv.26026. Epub 2020 May 25. PMID: 32449972; PMCID: PMC7280653. | | |
|  | Ghaffari *et al.* 2021 | No mental health 🡪 risk/outcome data |
| Ghaffari M, Ansari H, Beladimoghadam N, Aghamiri SH, Haghighi M, Nabavi M, . . . Lima BS. (2021).  Neurological features and outcome in COVID-19: dementia can predict severe disease. *J Neurovirol, 27*(1), 86-  93. doi:10.1007/s13365-020-00918-0 | | |
|  | Giannoglou *et al.* 2021 [MEDRXIV] | Mental health 🡪 mortality |
| Giannoglou D, Meimeti E, Provatopoulou X, Stathopoulos K, Roukas KI, Galanis P. (2020). Predictors of mortality in hospitalized COVID-19 patients in Athens, Greece. *medRxiv*. doi: https://doi.org/10.1101/2020.10.12.20211193 | | |
|  | Gilley *et al.* 2022 | No mental health 🡪 risk/outcome data |
| Gilley KN, Baroudi L, Yu M, Gainsburg I, Reddy N, Bradley C, et al. Risk Factors for COVID-19 in College Students Identified by Physical, Mental, and Social Health Reported During the Fall 2020 Semester: Observational Study Using the Roadmap App and Fitbit Wearable Sensors. JMIR mental health. 2022;9(2):e34645–e34645. | | |
|  | Goldberger *et al.* 2022 | Mental health 🡪 risk/outcome |
| Goldberger N, Bergman-Levy T, Haklai Z, Yoffe R, Davidson M, Susser E, et al. COVID-19 and severe mental illness in Israel: testing, infection, hospitalization, mortality and vaccination rates in a countrywide study. Molecular psychiatry. 2022;27(7):3107–14. | | |
|  | Góme-Atúnez *et al.* 2020 | No mental health 🡪 risk/outcome data |
| Gómez Antúnez M, Muiño Míguez A, Bendala Estrada AD, Maestro de la Calle G, Monge Monge D, Boixeda R, Ena J, Mella Pérez C, Anton Santos JM, Lumbreras Bermejo C; SEMI-COVID-19 Network. Clinical Characteristics and Prognosis of COPD Patients Hospitalized with SARS-CoV-2. Int J Chron Obstruct Pulmon Dis. 2021 Jan 5;15:3433-3445. doi: 10.2147/COPD.S276692. PMID: 33447021; PMCID: PMC7801905. | | |
|  | Gorji *et al.* 2022 | No mental health 🡪 risk/outcome data- anorexia |
| Gorji F, Shafiekhani S, Namdar P, Abdollahzade S, Rafiei S. Machine learning-based COVID-19 diagnosis by demographic characteristics and clinical data. Advances in respiratory medicine. 2022. | | |
|  | Govind *et al.* 2021 | Mental health 🡪 risk but only patients |
| Govind R, Fonseca de Freitas D, Pritchard M, Hayes RD, MacCabe JH. Clozapine treatment and risk of COVID-19 infection: retrospective cohort study. Br J Psychiatry. 2020 Jul 27:1-7. doi: 10.1192/bjp.2020.151. Epub ahead of print. PMID: 32713374; PMCID: PMC7417985. | | |
|  | Gu *et al.* 2020 | No mental health 🡪 risk/outcome data |
| Gu T, Mack JA, Salvatore M, Sankar SP, Valley TS, Singh K, . . . Mukherjee B. (2020). Characteristics  Associated With Racial/Ethnic Disparities in COVID-19 Outcomes in an Academic Health Care System. *JAMA*  *Netw Open, 3*(10). doi:10.1001/jamanetworkopen.2020.25197 | | |
|  | Guliani *et al.* 2022 | No mental health 🡪 risk/outcome data |
| Guliani A, Tandon A, Chakroborty A, Gupta PP. Predictors of post COVID complications in patients admitted with moderate to severe COVID symptoms: A single center, prospective, observational study. Monaldi archives for chest disease. 2022; | | |
|  | Gunal *et al.* 2020 | Exclusion based on language |
| Gunal O, Ture E, Bayburtlu M, Arslan U, Demirag MD, Taskin MH, Kilic, S. (2020). Evaluation of Patients  Diagnosed with COVID-19 in Terms of Risk Factors. *Mikrobiyol Bul, 54*(4), 575-582. doi:10.5578/mb.69811 | | |
|  | Guo *et al.* 2021 | No mental health 🡪 risk/outcome data |
| Guo T, Shen Q, Guo W, He W, Li J, Zhang Y, Wang Y, Zhou Z, Deng D, Ouyang X, Xiang Z, Jiang M, Liang M, Huang P, Peng Z, Xiang X, Liu W, Luo H, Chen P, Peng H. Clinical Characteristics of Elderly Patients with COVID-19 in Hunan Province, China: A Multicenter, Retrospective Study. Gerontology. 2020;66(5):467-475. doi: 10.1159/000508734. Epub 2020 May 29. PMID: 32474561. PMC7274101. | | |
|  | Gurrieri *et al.* 2021 | No mental health 🡪 risk/outcome data |
| Gurrieri L, Fairbairn CE, Sayette MA, Bosch N. Alcohol narrows physical distance between strangers. Proc Natl Acad Sci U S A. 2021 May 18;118(20):e2101937118. doi: 10.1073/pnas.2101937118. PMID: 33972448. | | |
|  | Gutiérrez-Rodríguez *et al.* 2020 | No mental health 🡪 risk/outcome data |
| Gutiérrez Rodríguez J, Montero Muñoz J, Jiménez Muela F, Guirola García-Prendes C, Martínez Rivera M, Gómez Armas L. Variables asociadas con mortalidad en una población de pacientes mayores de 80 años y con algún grado de dependencia funcional, hospitalizados por COVID-19 en un Servicio de Geriatría [Variables associated with mortality in a selected sample of patients older than 80 years and with some degree of functional dependence hospitalized for COVID-19 in a Geriatrics Service]. Rev Esp Geriatr Gerontol. 2020 Nov-Dec;55(6):317-325. Spanish. doi: 10.1016/j.regg.2020.07.002. Epub 2020 Jul 16. PMID: 32736821; PMCID: PMC7365050. | | |
|  | Gutiérrez-Sacristán *et al.* 2022 | No mental health 🡪 risk/outcome data |
| Gutiérrez-Sacristán A, Serret-Larmande A, Hutch MR, Sáez C, Aronow BJ, Bhatnagar S, Bonzel CL, Cai T, Devkota B, Hanauer DA, Loh NHW, Luo Y, Moal B, Ahooyi TM, Njoroge WFM, Omenn GS, Sanchez-Pinto LN, South AM, Sperotto F, Tan ALM, Taylor DM, Verdy G, Visweswaran S, Xia Z, Zahner J, Avillach P, Bourgeois  FT; Consortium for Clinical Characterization of COVID-19 by EHR (4CE). Hospitalizations Associated With Mental Health Conditions Among Adolescents in the US and France During the COVID-19 Pandemic. JAMA Netw Open. 2022 Dec 1;5(12):e2246548. doi: 10.1001/jamanetworkopen.2022.46548. PMID: 36512353;  PMCID: PMC9856226. | | |
|  | Guzek *et al.* 2022 | No mental health 🡪 risk/outcome data |
| Guzek A, Rybicki Z, Woźniak-Kosek A, Tomaszewski D. The Clinical Manifestation of SARS-CoV-2 in Critically Ill Patients with Klebsiella pneumoniae NDM Hospitalized in the ICU of a Modular Hospital during the Third Wave of the Pandemic in Poland-An Observational Cohort Study. Diagnostics (Basel). 2022;12(5):1118. | | |
|  | Haimovich *et al.* 2020 | Mental health 🡪 risk |
| Haimovich A, Warner F, Young HP, Ravindra NG, Sehanobish A, Gong G, Wilson FP, van Dijk D, Schulz W, Taylor RA. Patient factors associated with SARS-CoV-2 in an admitted emergency department population. J Am Coll Emerg Physicians Open. 2020 May 22;1(4):569–77. doi: 10.1002/emp2.12145. Epub ahead of print. PMID: 32838371; PMCID: PMC7280703. | | |
|  | Hamer *et al.* 2020 | No mental health 🡪 risk/outcome data |
| Hamer M, Kivimäki M, Gale CR, Batty GD. Lifestyle risk factors, inflammatory mechanisms, and COVID-19 hospitalization: A community-based cohort study of 387,109 adults in UK. Brain Behav Immun. 2020 Jul;87:184-187. doi: 10.1016/j.bbi.2020.05.059. Epub 2020 May 23. PMID: 32454138; PMCID: PMC7245300. | | |
|  | Harrison *et al.* 2021 | No mental health 🡪 risk/outcome data |
| Harrison E, Monroe-Lord L, Carson AD, Jean-Baptiste AM, Phoenix J, Jackson P, Harris BM, Asongwed E, Richardson ML. COVID-19 pandemic-related changes in wellness behavior among older Americans. BMC Public Health. 2021 Apr 19;21(1):755. doi: 10.1186/s12889-021-10825-6. PMID: 33874931; PMCID: PMC8054850. | | |
|  | Hashemi-Shahri *et al.* 2022 | Mental health 🡪 risk/outcome |
| Hashemi-Shahri SM, Tabatabaei SMN, Ansari-Moghaddam A, Mohammadi M, Okati-Aliabad H, Tabatabaei SM, et al. Epidemiological and clinical risk factors related to severe COVID-19 in Iran: a multi-center study. BMC infectious diseases. 2022;22(1):184–184. | | |
|  | Hashim *et al.* 2020 | No mental health 🡪 risk/outcome data |
| Hashim, M. J., Alsuwaidi, A. R., & Khan, G. (2020). Population Risk Factors for COVID-19 Mortality  in 93 Countries. *J Epidemiol Glob Health, 10*(3), 204-208. doi:10.2991/jegh.k.200721.001 | | |
|  | Hatakeyama *et al.* 2022 | No mental health 🡪 risk/outcome data |
| Hatakeyama J, Inoue S, Liu K, Yamakawa K, Nishida T, Ohshimo S, Hashimoto S, Kanda N, Maruyama S, Ogata Y, Kawakami D, Shimizu H, Hayakawa K, Tanaka A, Oshima T, Fuchigami T, Yawata H, Oe K, Kawauchi A, Yamagata H, Harada M, Sato Y, Nakamura T, Sugiki K, Hakozaki T, Beppu S, Anraku M, Kato N, Iwashita T, Kamijo H, Kitagawa Y, Nagashima M, Nishimaki H, Tokuda K, Nishida O, Nakamura K. Prevalence and Risk Factor Analysis of Post-Intensive Care Syndrome in Patients with COVID-19 Requiring Mechanical Ventilation: A Multicenter Prospective Observational Study. J Clin Med. 2022 Sep 28;11(19):5758. doi: 10.3390/jcm11195758. PMID: 36233627; PMCID: PMC9571505. | | |
|  | Heald *et al.* 2022 | No mental health 🡪 risk/outcome data |
| Heald AH, Jenkins DA, Williams R, Sperrin M, Fachim H, Mudaliar RN, et al. The Risk Factors Potentially Influencing Hospital Admission in People with Diabetes, Following SARS-CoV-2 Infection: A Population-Level Analysis. Diabetes therapy. 2022;13(5):1007–21. | | |
|  | Hedberg *et al.* 2023 | Mental health 🡪 longcovid |
| Hedberg P, Granath F, Bruchfeld J, Askling J, Sjöholm D, Fored M, Färnert A, Naucler P. Post COVID-19 condition diagnosis: A population-based cohort study of occurrence, associated factors, and healthcare use by severity of acute infection. J Intern Med. 2023 Feb;293(2):246-258. doi: 10.1111/joim.13584. Epub  2022 Dec 7. PMID: 36478477; PMCID: PMC9877994. | | |
|  | Heesakkers *et al.* 2022 | No mental health 🡪 risk/outcome data |
| Heesakkers H, van der Hoeven JG, Corsten S, Janssen I, Ewalds E, Simons KS, et al. Clinical Outcomes Among Patients With 1-Year Survival Following Intensive Care Unit Treatment for COVID-19. JAMA : the journal of the American Medical Association. 2022;327(6):559–65. | | |
|  | Hesni *et al.* 2022 | Mental health 🡪 mortality |
| Hesni E, Sayad B, Khosravi Shadmani F, Najafi F, Khodarahmi R, Rahimi Z, et al. Demographics, clinical characteristics, and outcomes of 27,256 hospitalized COVID-19 patients in Kermanshah Province, Iran: a retrospective one-year cohort study. BMC infectious diseases. 2022;22(1):319–319. | | |
|  | Hirakawa *et al.* 2021 | No mental health 🡪 risk/outcome data |
| Hirakawa H, Ishii N. Association between mental illness and COVID-19 in South Korea. Lancet Psychiatry. 2021 Apr;8(4):270-271. doi: 10.1016/S2215-0366(20)30539-3. Epub 2021 Feb 19. PMID: 33617760; PMCID: PMC7906740. | | |
|  | Hirashima *et al.* 2021 | Mental health 🡪 course |
| Hirashima T, Arai T, Kitajima H, Tamura Y, Yamada T, Hashimoto S, Morishita H, Minamoto S, Kawashima K, Kashiwa Y, Kameda M, Takeshita T, Suzuki H, Matsuoka H, Yamaguchi S, Tanaka T, Nagai T 2021. Factors significantly associated with COVID-19 severity in symptomatic patients: A retrospective single-center study. Journal of Infection and Chemotherapy 27 (1), 76–82. | | |
|  | Hoertel *et al.* 2021 | No mental health 🡪 risk/outcome data |
| Hoertel N, Sánchez-Rico M, Cougoule C, Gulbins E, Kornhuber J, Carpinteiro A, Becker KA, Reiersen AM, Lenze EJ, Seftel D, Lemogne C, Limosin F. Repurposing antidepressants inhibiting the sphingomyelinase acid/ceramide system against COVID-19: current evidence and potential mechanisms. Mol Psychiatry. 2021  Dec;26(12):7098-7099. doi: 10.1038/s41380-021-01254-3. Epub 2021 Aug 12. PMID:  34385600; PMCID: PMC8359627. | | |
|  | Hoertel *et al.* 2021 | Mental health 🡪 mortality |
| Hoertel N, Sánchez-Rico M, Vernet R, Beeker N, Jannot AS, Neuraz A, Salamanca E, Paris N, Daniel C, Gramfort A, Lemaitre G, Bernaux M, Bellamine A, Lemogne C, Airagnes G, Burgun A, Limosin F; AP-HP / Universities / INSERM COVID-19 Research Collaboration and AP-HP COVID CDR Initiative. Association between antidepressant use and reduced risk of intubation or mortality in hospitalized patients with COVID-19: results from an observational study. Mol Psychiatry. 2021 Feb 4. doi: 10.1038/s41380-021-01021-4. Epub ahead of print. PMID: 33536545. | | |
|  | Hölzle *et al.* 2020 | No mental health 🡪 risk/outcome data |
| Hölzle P, Aly L, Frank W, Förstl H, Frank A. COVID-19 distresses the depressed while schizophrenic patients are unimpressed: A study on psychiatric inpatients. Psychiatry Res. 2020 Sep;291:113175. doi: 10.1016/j.psychres.2020.113175. Epub 2020 Jun 5. PMID: 32535514; PMCID: | | |
|  | Hossain *et al.* 2020 | No mental health 🡪 risk/outcome data |
| Hossain MK, Hassanzadeganroudsari M, Apostolopoulos V. (2020). Why METH users are at high risk of fatality  due to COVID-19 infection? *Expert Rev Vaccines, 19*(12), 1101-1103. doi:10.1080/14760584.2020.1858059 | | |
|  | Huang *et al.* 2021 | Meta-analysis mechanisms: alcohol – infection risk |
| Huang W, Zhou H, Hodgkinson C, Montero A, Goldman D, Chang SL. Network Meta-Analysis on the Mechanisms Underlying Alcohol Augmentation of COVID-19 Pathologies. Alcohol Clin Exp Res. 2021 Apr;45(4):675-688. doi: 10.1111/acer.14573. Epub 2021 Mar 20. PMID: 33583045; PMCID: PMC8014161. | | |
|  | Huang *et al.* 2021 [MEDRXIV] | No mental health 🡪 risk/outcome data |
| Huang Y, Pinto MD, Borelli JL, Mehrabadi MA, Abrihim H, Dutt N, Lambert N, Nurmi EL, Chakraborty R, Rahmani AM, Downs CA. COVID Symptoms, Symptom Clusters, and Predictors for Becoming a Long-Hauler: Looking for Clarity in the Haze of the Pandemic. medRxiv [Preprint]. 2021 Mar 5:2021.03.03.21252086. doi: 10.1101/2021.03.03.21252086. PMID: 33688670; PMCID: PMC7941647. | | |
|  | Ikemura *et al.* 2021 | No mental health 🡪 risk/outcome data |
| Ikemura K, Bellin E, Yagi Y, Billett H, Saada M, Simone K, Stahl L, Szymanski J, Goldstein DY, Reyes Gil M. Using Automated Machine Learning to Predict the Mortality of Patients With COVID-19: Prediction Model Development Study. J Med Internet Res. 2021 Feb 26;23(2):e23458. doi: 10.2196/23458. PMID: 33539308; PMCID: PMC7919846. | | |
|  | Iqbal *et al.* 2020 | No mental health 🡪 risk/outcome data |
| Iqbal Y, Al Abdulla MA, Albrahim S, Latoo J, Kumar R, Haddad PM. Psychiatric presentation of patients with acute SARS-CoV-2 infection: a retrospective review of 50 consecutive patients seen by a consultation-liaison psychiatry team. BJPsych Open. 2020 Sep 10;6(5):e109. doi: 10.1192/bjo.2020.85. PMID: 32907692; PMCID: PMC7484218. | | |
|  | Ismael *et al.* 2021 | No mental health 🡪 risk/outcome data |
| Ismael F, Zaramella B, Battagin T, Bizario JCS, Gallego J, Villela V, de Queiroz LB, Leal FE, Torales J, Ventriglio A, Marziali ME, Gonçalves PD, Martins SS, Castaldelli-Maia JM. Substance Use in Mild-COVID-19 Patients: A Retrospective Study. Front Public Health. 2021 Mar 4;9:634396. doi: 10.3389/fpubh.2021.634396. PMID: 33748068; PMCID: PMC7969785. | | |
|  | Izurieta *et al.* 2020 | Mental health 🡪 course, mortality |
| Izurieta HS, Graham DJ, Jiao Y, Hu M, Lu Y, Wu Y, Chillarige Y, Wernecke M, Menis M, Pratt D, Kelman J, Forshee R. Natural History of Coronavirus Disease 2019: Risk Factors for Hospitalizations and Mortalitys Among >26 Million US Medicare Beneficiaries. J Infect Dis. 2021 Mar 29;223(6):945-956. doi: 10.1093/infdis/jiaa767. PMID: 33325510; PMCID: PMC7799044. | | |
|  | Jabalameli *et al.* 2022 | Overlap with Wang *et al.* 2020 |
| Jabalameli MR, Zhang ZD. Substance abuse and the risk of severe COVID-19: Mendelian randomization confirms the causal role of opioids but hints a negative causal effect for cannabinoids. Front Genet. 2022 Dec 13;13:1070428. doi: 10.3389/fgene.2022.1070428. PMID: 36583016; PMCID: PMC9792508. | | |
|  | Jacobs *et al.* 2023 | No mental health 🡪 risk/outcome data |
| Jacobs ET, Catalfamo CJ, Colombo PM, Khan SM, Austhof E, Cordova-Marks F, Ernst KC, Farland LV, Pogreba-Brown K. Pre-existing conditions associated with post-acute sequelae of COVID-19. J Autoimmun. 2023 Feb;135:102991. doi:10.1016/j.jaut.2022.102991. Epub 2023 Jan 6. PMID: 36634460; PMCID: PMC9816074. | | |
|  | Jagadeesh *et al.* 2022 | No mental health 🡪 risk/outcome data |
| Jagadeesh N, Deva V, Kapadi S, Shaw D. Risk Factors of 120-Day Mortality Among Hip Fractures With Concomitant COVID-19 Infection. Cureus. 2022 Dec 17;14(12):e32637. doi: 10.7759/cureus.32637. PMID: 36545355; PMCID: PMC9762522. | | |
|  | Jalodia *et al.* 2022 | No mental health 🡪 risk/outcome data |
| Jalodia R, Antoine D, Braniff RG, Dutta RK, Ramakrishnan S, Roy S. Opioid-Use, COVID-19 Infection, and Their Neurological Implications. Frontiers in neurology. 2022;13:884216–884216. | | |
|  | Jang *et al.* 2021 | No mental health 🡪 risk/outcome data |
| Jang SY, Seon JY, Eun BL, Koh SB, Yoo JH, Lee WY, . . . Chang SG (2021). Risk Factors of Outcomes of  COVID-19 Patients in Korea: Focus on Early Symptoms. *J Korean Med Sci, 36*(18), e132.  doi:10.3346/jkms.2021.36.e132 | | |
|  | Jegede *et al.* 2021 | No comparison group for course |
| Jegede, O., Raman, A. A., Tiongson, B., Garlapati, P. R., Hershberger, J., & Gayam, V. (2021). Clinical  characteristics, hospital course, and outcomes among COVID-19 positive patients with mental illness in a  community hospital in New York City. *International Journal of Mental Health, 50*(1), 4-15.  doi:10.1080/00207411.2020.1845567 | | |
|  | Jemberi *et al.* 2020 | No mental health 🡪 risk/outcome data |
| Jemberie WB, Stewart Williams J, Eriksson M, Grönlund AS, Ng N, Blom Nilsson M, Padyab M, Priest KC, Sandlund M, Snellman F, McCarty D, Lundgren LM. Substance Use Disorders and COVID-19: Multi-Faceted Problems Which Require Multi-Pronged Solutions. Front Psychiatry. 2020 Jul 21;11:714. doi: 10.3389/fpsyt.2020.00714. PMID: 32848907; PMCID: PMC7396653. | | |
|  | Jeon *et al.* 2020 | Mental health 🡪 risk, course, mortality |
| Jeon H-L, Kwon JS, Park S-H, Shin J-Y 2020. Association of mental disorders with SARS-CoV-2 infection and severe health outcomes: a nationwide cohort study, BJP, 2020.2008.2005.20169201. | | |
|  | Ji *et al.* 2020 | Double data South Korea nation wide |
| Ji W, Huh K, Kang M, Hong J, Bae GH, Lee R, . . . Jung J (2020). Effect of Underlying Comorbidities on the  Infection and Severity of COVID-19 in Korea: a Nationwide Case-Control Study. *J Korean Med Sci, 35*(25).  doi:10.3346/jkms.2020.35.e237 | | |
|  | Jones *et al.* 2021 | Mental health 🡪 longcovid |
| Jones R, Davis A, Stanley B, Julious S, Ryan D, Jackson DJ, Halpin DMG, Hickman K, Pinnock H, Quint JK, Khunti K, Heaney LG, Oliver P, Siddiqui S, Pavord I, Jones DHM, Hyland M, Ritchie L, Young P, Megaw T, Davis S, Walker S, Holgate S, Beecroft S, Kemppinen A, Appiagyei F, Roberts EJ, Preston M, Hardjojo A, Carter V, van Melle M, Price D. Risk Predictors and Symptom Features of Long COVID Within a Broad Primary Care Patient Population Including Both Tested and Untested Patients. Pragmat Obs Res. 2021 Aug 11;12:93-104. doi:10.2147/POR.S316186. PMID:34408531; PMCID: PMC8366779. | | |
|  | Karaoulanis *et al.* 2021 | Review |
| Karaoulanis SE, Christodoulou NG. Do patients with schizophrenia have higher infection and mortality rates due to COVID-19? A systematic review. Psychiatrikē 2021;32(3):219–23. | | |
|  | Karthaka *et al.* 2021 | No mental health 🡪 risk/outcome data |
| Karthaka C, Baliga S, Walvekar P. Clinical profile and outcomes of COVID-19 positive patients -A cross sectional study. Journal of family medicine and primary care. 2021;10(11):4036–40. | | |
|  | Kerstes *et al.* 2023 | No mental health 🡪 risk/outcome data |
| Jennifer K, Shirley SBD, Avi P, Daniella RC, Naama SS, Anat EZ, Miri MR. Post-acute sequelae of COVID-19 infection. Prev Med Rep. 2023 Feb;31:102097. doi: 10.1016/j.pmedr.2022.102097. Epub 2022 Dec 21. PMID: 36567743; PMCID: PMC9767882. | | |
|  | Khalaf *et al.* 2022 | No mental health 🡪 risk/outcome data |
| Khalaf M, Alboraie M, Abdel-Gawad M, Abdelmalek M, Abu-Elfatth A, Abdelhamed W, et al. Prevalence and Predictors of Persistent Symptoms After Clearance of SARS-CoV-2 Infection: A Multicenter Study from Egypt. Infection and drug resistance. 2022;15:2575–87. | | |
|  | Khirsagar *et al.* 2022 | No mental health 🡪 risk/outcome data |
| Kshirsagar M, Nasir M, Mukherjee S, Becker N, Dodhia R, Weeks WB, Ferres JL, Richardson B. The Risk of Hospitalization and Mortality After Breakthrough SARS- CoV-2 Infection by Vaccine Type: Observational Study of Medical Claims Data. JMIR Public Health Surveill. 2022 Nov 8;8(11):e38898. doi: 10.2196/38898. PMID: 36265135; PMCID: PMC9645422. | | |
|  | Kianersi *et al.* 2021 | No mental health 🡪 risk/outcome data |
| Kianersi S, Ludema C, Macy JT, Garcia Colato E, Chen C, Luetke M, et al. A Cross-Sectional Analysis of Demographic and Behavioral Risk Factors of Severe Acute Respiratory Syndrome Coronavirus 2 Seropositivity Among a Sample of U.S. College Students. Journal of adolescent health. 2021;69(2):219–26. | | |
|  | Kim *et al.* 2021 | Mental health 🡪 mortality |
| Kim E, Kim YC, Park JY, Jung J, Lee JP, Kim H. (2021). Evaluation of the Prognosisof COVID-19 patients  According to the Presence of Underlying Diseases and Drug Treatment. *Int J Environ Res Public Health, 18*(10).  doi:10.3390/ijerph18105342 | | |
|  | Kim *et al.* 2022 | Overlap in data |
| Kim J, Park SH, Kim JM. Effect of Comorbidities on the Infection Rate and Severity of COVID-19: Nationwide Cohort Study With Propensity Score Matching. JMIR Public Health Surveill. 2022 Nov 18;8(11):e35025. doi: 10.2196/35025. PMID: 36265125; PMCID: PMC9678330. | | |
|  | Kirov *et al.* 2021 [MEDRXIV] | Overlap in data |
| Kirov G, Baker E. (2021). COVID-19 and mortality risk in patients with psychiatric disorders. *medRxiv*. | | |
|  | Kolin *et al.* 2021 | Overlap in data |
| Kolin DA, Kulm S, Christos PJ, Elemento O. Clinical, regional, and genetic characteristics of Covid-19 patients from UK Biobank. PLoS One. 2020 Nov 17;15(11):e0241264. doi: 10.1371/journal.pone.0241264. PMID: 33201886; PMCID: PMC7671499. | | |
|  | Kondakov *et al.* 2021 | No mental health 🡪 risk/outcome data |
| Kondakov A, Berdalin A, Lelyuk V, Gubskiy I, Golovin D. Risk Factors of In-Hospital Mortality in Non-Specialized Tertiary Center Repurposed for Medical Care to COVID-19 Patients in Russia. Diagnostics (Basel). 2021;11(9):1687. | | |
|  | Kozloff *et al.* 2020 | No mental health 🡪 risk/outcome data |
| Kozloff N, Mulsant BH, Stergiopoulos V, Voineskos AN. The COVID-19 Global Pandemic: Implications for People With Schizophrenia and Related Disorders. Schizophr Bull. 2020 Jul 8;46(4):752-757. doi: 10.1093/schbul/sbaa051. PMID: 32343342; PMCID: PMC7197583. Y | | |
|  | Kundi *et al.* 2020 | Mental health 🡪 mortality |
| Kundi H, Çetin EHÖ, Canpolat U, Aras S, Celik O, Ata N, Birinci S, Çay S, Özeke Ö, Tanboğa IH, Topaloğlu S.  The role of Frailty on Adverse Outcomes Among Older Patients with COVID-19. J Infect. 2020 Dec;81(6):944-  951. doi: 10.1016/j.jinf.2020.09.029. Epub 2020 Sep 28. PMID: 33002560; PMCID: PMC7521439. | | |
|  | Lagrandeur *et al.* 2023 | No mental health 🡪 risk/outcome data |
| Lagrandeur J, Putallaz P, Krief H, Büla CJ, Coutaz M. Mortality in COVID-19,lder patients hospitalized in a geriatric ward: Is obesity protective? BMC Geriatr. 2023 Apr 11;23(1):228. doi: 10.1186/s12877-023-03937-8. PMID: 37041477; PMCID: PMC10088129. | | |
|  | Landén *et al.* 2021 | No mental health 🡪 risk/outcome data |
| Landén M, Larsson H, Lichtenstein P, Westin J, Song J. Respiratory infections during lithium and valproate medication: a within-individual prospective study of 50,000 patients with bipolar disorder. Int J Bipolar Disord. 2021 Feb 1;9(1):4. doi: 10.1186/s40345-020-00208-y. PMID: 33521836; PMCID: PMC7847747. | | |
|  | Landes *et al.* 2021 | No mental health 🡪 risk/outcome data |
| Landes SD, Turk MA, Formica MK, McDonald KE, Stevens JD. COVID-19 outcomes among people with intellectual and developmental disability living in residential group homes in New York State. Disabil Health J. 2020 Oct;13(4):100969. doi: 10.1016/j.dhjo.2020.100969. Epub 2020 Jun 24. PMID: 32600948; PMCID: PMC7311922. | | |
|  | Laskovski *et al.* 2023 | No mental health 🡪 risk/outcome data |
| Laskovski L, Felcar JM, Fillis MMA, Trelha CS. Risk factors associated with limited functional status among out-of-hospital patients 30 days and one year after a diagnosis of COVID-19: a cohort study. Sci Rep. 2023 Mar 3;13(1):3584. doi: 10.1038/s41598-023-30674-0. PMID: 36869060; PMCID: PMC9982776. | | |
|  | Lassale *et al.* 2020 | No mental health 🡪 risk/outcome data |
| Lassale C, Gaye B, Hamer M, Gale CR, Batty GD. (2020). Ethnic disparities in hospitalisation for COVID-19 in  England: The role of socioeconomic factors, mental health, and inflammatory and pro-inflammatory factors in  a community-based cohort study. *Brain Behavior and Immunity, 88*, 44-49. doi:10.1016/j.bbi.2020.05.074 | | |
|  | Lassen *et al.* 2020 | No mental health 🡪 risk/outcome data |
| Lassen MCH, Skaarup KG, Sengeløv M, Iversen K, Ulrik CS, Jensen JUS, Biering-Sørensen T. Alcohol Consumption and the Risk of Acute Respiratory Distress Syndrome in COVID-19. Ann Am Thorac Soc. 2020 Dec 14. doi: 10.1513/AnnalsATS.202008-988RL. Epub ahead of print. PMID: 33315543. | | |
|  | Lebin *et al.* 2020 | Mental health 🡪 risk |
| Lebin JA, Mudan A, Wu AHB. Chronic alcohol use does not protect against COVID-19 infection. Am J Emerg Med. 2020 Nov 14:S0735-6757(20)31033-0. doi: 10.1016/j.ajem.2020.11.024. Epub ahead of print. PMID: 33221114; PMCID: PMC7666530. | | |
|  | Lee 2021 | Not available |
| Lee S. (2021). Clinical outcomes in COVID-19 patients with severe mental illness: A comparison between  community-acquired and outbreak at sanatorium. *Asia-Pacific Psychiatry, 13*. | | |
|  | Lee *et al.* 2021 a | Double data |
| Lee DY, Cho J, You SC, Park RW, Kim CS, Lee EY, Aizenstein H, Andreescu C, Karim H, Hong CH, Rho HW, Park B, Son SJ. Risk of Mortality in Elderly Coronavirus Disease 2019 Patients With Mental Health Disorders: A Nationwide Retrospective Study in South Korea. Am J Geriatr Psychiatry. 2020 Dec;28(12):1308-1316. doi: 10.1016/j.jagp.2020.09.016. Epub 2020 Sep 28. PMID:33023798; PMCID: PMC7521355. | | |
|  | Lee *et al.* 2021 b The Lancet | Double data |
| Lee SW, Yang JM, Moon SY, Yoo IK, Ha EK, Kim SY, Park UM, Choi S, Lee SH, Ahn YM, Kim JM, Koh HY, Yon DK. Association between mental illness and COVID-19 susceptibility and clinical outcomes in South Korea: a nationwide cohort study. Lancet Psychiatry. 2020 Dec;7(12):1025-1031. doi: 10.1016/S2215-0366(20)30421-1. Epub 2020 Sep 17. PMID: 32950066; PMCID: PMC7498216.  Lee SW, Yang JM, Moon SY, Kim N, Ahn YM, Kim JM, Shin JI, Suh DI, Yon DK; study authors. Association between mental illness and COVID-19 in South Korea: a post-hoc analysis. Lancet Psychiatry. 2021 Apr;8(4):271-272. doi: 10.1016/S2215-0366(21)00043-2. Epub 2021 Feb 19. | | |
|  | Lee *et al.* 2023 | No mental health 🡪 risk/outcome data |
| Lee K, Jang K, Kim H, Bae G, Jang CS, Shin JH. Factors Affecting the Length of Stay in the Emergency Department in Psychiatric Emergency Patients in the COVID-19 Pandemic Context. Inquiry. 2023 Jan-Dec;60:469580231167529. doi:10.1177/00469580231167529. PMID: 37052169; PMCID: PMC10102821. | | |
|  | Lega *et al.* 2021 | Mental health 🡪 mortality |
| Lega I, Nisticò L, Palmieri L, Caroppo E, Lo Noce C, Donfrancesco C, . . . Onder G. (2021). Psychiatric  disorders among hospitalized patients deceased with COVID-19 in Italy. *EClinicalMedicine, 35*, 100854.  doi:10.1016/j.eclinm.2021.100854 | | |
|  | Leite *et al.* 2021 | No mental health 🡪 risk/outcome data |
| Leite VF, Rampim DB, Jorge VC, de Lima MDCC, Cezarino LG, da Rocha CN, Esper RB; Prevent Senior COVID-19 Rehabilitation Study. Persistent Symptoms and Disability After COVID-19 Hospitalization: Data From a Comprehensive Telerehabilitation Program. Arch Phys Med Rehabil. 2021 Mar 10:S0003-9993(21)00225-2. doi: 10.1016/j.apmr.2021.03.001. | | |
|  | Li and Hua 2021 | No mental health 🡪 risk/outcome data |
| Li S, Hua X. Modifiable lifestyle factors and severe COVID-19 risk: a Mendelian randomisation study. BMC Med Genomics. 2021 Feb 3;14(1):38. doi: 10.1186/s12920-021-00887-1. PMID: 33536004; PMCID: PMC7856619. | | |
|  | Li *et al.* 2020 | No mental health 🡪 risk/outcome data |
| Li J, Long X, Zhang Q, Fang X, Li N, Fedorova B, Hu S, Li J, Xiong N, Lin Z. Lee DY, Cho J, You SC, Park RW, Kim CS, Lee EY, Aizenstein H, Andreescu C, Karim H, Hong CH, Rho HW, Park B, Son SJ. Risk of Mortality in Elderly Coronavirus Disease 2019 Patients With Mental Health Disorders: A Nationwide Retrospective Study in South Korea. Am J Geriatr Psychiatry. 2020 Dec;28(12):1308-1316. doi: 10.1016/j.jagp.2020.09.016. Epub 2020 Sep 28. PMID: 33023798; PMCID: PMC7521355. Y | | |
|  | Li *et al.* 2021 | No mental health 🡪 risk/outcome data |
| Li J, Long X, Zhang Q, Fang X, Li N, Federova B, Hu S, Li Jh, Xiong N, Lin Z. Tobacco smoking confers risk for severe COVID-19 unexplainable by pulmonary imaging. J Intern Med. 2021 Apr;289(4):574-583. doi: 10.1111/joim.13190. Epub 2020 Dec 3. PMID: 33270312; PMCID: PMC7753648. | | |
|  | Li *et al.* 2022 | Mental health 🡪 outcome data |
| Li T, Zhang L, Cai S, Lu Z, Bao W, Guo Z, et al. https://cphds.sph.umich.edu:8443/covidphewas/ Journal of affective disorders. 2022;312:331–6. | | |
|  | Liu *et al.* 2020 | No mental health 🡪 risk/outcome data |
| Liu FY, Sun XL, Zhang Y, Ge L, Wang J, Liang X, Li JF, Wang CL, Xing ZT, Chhetri JK, Sun P, Chan P. Evaluation of the Risk Prediction Tools for Patients With Coronavirus Disease 2019 in Wuhan, China: A Single-Centered, Retrospective, Observational Study. Crit Care Med. 2020 Nov;48(11): e1004-e1011. doi: 10.1097/CCM.0000000000004549. PMID: 32897668; PMCID: PMC7448719. | | |
|  | Livingston *et al.* 2020 | No mental health 🡪 risk/outcome data |
| Livingston G, Rostamipour H, Gallagher P, Kalafatis C, Shastri A, Huzzey L, . . . Marston L (2020). Prevalence,  management, and outcomes of SARS-CoV-2 infections in older people and those with dementia in mental  health wards in London, UK: a retrospective observational study. *Lancet Psychiatry, 7*(12), 1054-1063.  doi:10.1016/s2215-0366(20)30434-x | | |
|  | Lok *et al.* 2023 | No mental health 🡪 risk/outcome data |
| Lok N, Aydın Z, Uzun G, Kayaaslan B, Selçuk Tosun A. Relationship of Depression, Hopelessness and Life Satisfaction With Mortality Anxiety in Individuals Who Have Had COVID-19. Omega (Westport). 2023 May 10:302228231174602. doi: 10.1177/00302228231174602. PMID: 37161306; PMCID: PMC10183336. | | |
|  | Luykx *et al.* 2021 | No mental health 🡪 risk/outcome data |
| Luykx JJ, Lin BD. Are psychiatric disorders risk factors for COVID-19 susceptibility and severity? a two-sample, bidirectional, univariable, and multivariable Mendelian Randomization study. Transl Psychiatry. 2021 Apr 8;11(1):210. doi: 10.1038/s41398-021-01325-7. PMID: 33833219; PMCID: PMC8027711. | | |
|  | Ma *et al.* 2021 | No mental health 🡪 risk/outcome data |
| Ma X, Gao Y, Di L, Ma H, Mei B, Zhang J, Wang A, Feng K, Yang L, Chen Z. Characteristics of 1738 Patients with Coronavirus Disease 2019 (COVID-19) in Wuhan, China. Disaster Med Public Health Prep. 2021 Apr 30:1-20. doi: 10.1017/dmp.2021.129. Epub ahead of print. PMID: 33926610. | | |
|  | Maguire and Looi, 2020 | Not available |
| Maguire PA, Looi JCL. (2020). Vulnerability of people with schizophrenia to COVID-19. *Australian and New Zealand Journal of Psychiatry, 54*(10), 1044-1044. doi:10.1177/0004867420940775 | | |
|  | Mahmoud *et al.* 2021 | No mental health 🡪 risk/outcome data, ptsd |
| Mahmoud MH, Alghamdi FA, Alghamdi GA, Alkhotani LA, Alrehaili MA, El-Deeb DK. Study of Post-COVID-19 Syndrome in Saudi Arabia. Curēus (Palo Alto, CA). 2021;13(9):e17787–e17787. | | |
|  | Mallet *et al.* 2021 | No mental health 🡪 risk/outcome data |
| Mallet V, Beeker N, Bouam S, Sogni P, Pol S (2021). Prognosis of French COVID-19 patients with chronic liver  disease: a national retrospective cohort study for 2020. *J Hepatol*. doi:10.1016/j.jhep.2021.04.052 | | |
|  | Maregoni *et al.* 2021 | No mental health 🡪 risk/outcome data |
| Marengoni, A., Zucchelli, A., Grande, G., Fratiglioni, L., & Rizzuto, D. (2020). The impact of delirium on  outcomes for older adults hospitalised with COVID--19. *Age Ageing, 49*(6), 923-926.  doi:10.1093/ageing/afaa189 | | |
|  | Marel *et al.* 2021 | Opinion paper |
| Marel C, Mills KL, Teesson M. Substance use, mental disorders and COVID-19: a volatile mix. Curr Opin Psychiatry. 2021 Mar 17. doi: 10.1097/YCO.0000000000000707. Epub ahead of print. PMID: 33741762. | | |
|  | Marimuthu *et al.* 2021 | No mental health 🡪 risk/outcome data |
| Marimuthu Y, Kunnavil R, Anil NS, Nagaraja SB, Satyanarayana N, Kumar J, Ramya, B (2021). Clinical profile  and risk factors for mortality among COVID-19 inpatients at a tertiary care centre in Bengaluru, India. *Monaldi*  *Arch Chest Dis*. doi:10.4081/monaldi.2021.1724 | | |
|  | Maripuu *et al.* 2021 b | Mental health 🡪 risk |
| Maripuu M, Bendix M, Öhlund L, Widerström M, Werneke U. Mortality Associated With Coronavirus (COVID-19) Infection in Individuals With Severe Mental Disorders in Sweden During the Early Months of the Outbreak-An Exploratory Cross-Sectional Analysis of a Population-Based Register Study. Front Psychiatry. 2021 Jan 8;11:609579. doi: 10.3389/fpsyt.2020.609579. PMID: 33488430; PMCID: PMC7819873. | | |
|  | Martín-Rodríguez *et al.* 2021 | No mental health 🡪 risk/outcome data |
| Martín-Rodríguez F, Sanz-García A, Alberdi Iglesias A, Ortega Rabbione G, Del Pozo Vegas C, de la Torre-Díez I, et al. Mortality risk model for patients with suspected COVID-19 based on information available from an emergency dispatch center. Emergencias : revista de la Sociedad Espanola de Medicina de Emergencias. 2021;33(4):265–72. | | |
|  | Matalon *et al.* 2021 | No mental health 🡪 risk/outcome data |
| Matalon N, Dorman-Ilan S, Hasson-Ohayon I, Hertz-Palmor N, Shani S, Basel D, Gross R, Chen W, Abramovich A, Afek A, Ziv A, Kreiss Y, Pessach IM, Gothelf D. Trajectories of post-traumatic stress symptoms, anxiety, and depression in hospitalized COVID-19 patients: A one-month follow-up. J Psychosom Res. 2021 Apr;143:110399. doi: 10.1016/j.jpsychores.2021.110399. Epub 2021 Feb 16. PMID: 33618149; PMCID: PMC7885629. | | |
|  | Mazibas *et al.* 2023 | No mental health 🡪 risk/outcome data |
| Mazibas H, Speybroeck N, Dhondt E, Lambrecht S, Goorts K. Long COVID in the Belgian Defence forces: prevalence, risk factors and impact on quality of daily functioning. BMJ Mil Health. 2023 Feb 24:e002280. doi:  10.1136/military-2022-002280. Epub ahead of print. PMID: 36828639. | | |
|  | McKeigue *et al.* 2021 | No mental health 🡪 risk/outcome data |
| McKeigue PM, Weir A, Bishop J, McGurnaghan S, Kennedy S, McAllister D, Robertson C, Wood R, Lone N, Murray J, Caparrotta T, Smith-Palmer A, Goldberg D, McMenamin J, Ramsay C, Hutchinson S, Colhoun HM 2020. Rapid Epidemiological Analysis of Comorbidities and Treatments as risk factors for COVID-19 in Scotland (REACT-SCOT): a population-based case-control study, 2020.2005.2028.20115394. | | |
|  | McKetta *et al.* 2021 | No mental health 🡪 risk/outcome data |
| McKetta S, Morrison CN, Keyes KM. Trends in US Alcohol Consumption Frequency During the First Wave of the SARS-CoV-2 Pandemic. Alcohol Clin Exp Res. 2021 Apr;45(4):773-783. doi: 10.1111/acer.14575. Epub 2021 Mar 3. PMID: 33587290; PMCID: PMC8014717. | | |
|  | Meinlschmidt *et al.* 2022 | Mental health 🡪 risk/outcome |
| Meinlschmidt G, Guemghar S, Roemmel N, Battegay E, Hunziker S, Schaefert R. Depressive symptoms, but not anxiety, predict subsequent diagnosis of Coronavirus disease 19: a national cohort study. Epidemiology and psychiatric sciences. 2022;31:e16–e16. | | |
|  | McLaughlin *et al.* 2022 | SUD 🡪 mortality; but overlap |
| McLaughlin A, Burns R, Ryan M, Abbasi W, Harvey L, Hicks J, Sinha P, Assoumou SA. Comparing COVID-19-related Morbidity and Mortality Between Patients With and Without Substance Use Disorders: A Retrospective Cohort Study. Subst Abuse. 2023 Mar 22;17:11782218231160014. doi: 10.1177/11782218231160014. PMID: 36968974; PMCID: PMC10034287. | | |
|  | Mena *et al.* 2021 | No mental health 🡪 risk/outcome data |
| Mena GE, Martinez PP, Mahmud AS, Marquet PA, Buckee CO, Santillana M. Socioeconomic status determines COVID-19 incidence and related mortality in Santiago, Chile. Science. 2021 Apr 27:eabg5298. doi: 10.1126/science.abg5298. Epub ahead of print. PMID: 33906968 | | |
|  | Merzon *et al.* 2020 | Mental health 🡪 risk |
| Merzon E, Manor I, Rotem A, Schneider T, Vinker S, Golan Cohen A, Lauden A, Weizman A, Green I. ADHD as a Risk Factor for Infection With Covid-19. J Atten Disord. 2020 Jul 22:1087054720943271. doi: 10.1177/1087054720943271. Epub ahead of print. PMID: 32697120. | | |
|  | Merzon *et al.* 2021 | Mental health 🡪 course |
| Merzon E, Weiss MD, Cortese S, Rotem A, Schneider T, Craig SG, ... & Manor I. (2021). The Association  between ADHD and the Severity of COVID-19 Infection. *Journal of Attention Disorders*, 10870547211003659.  doi:10.1177/10870547211003659 | | |
|  | Moayed *et al.* 2021 | No mental health 🡪 risk/outcome data |
| Moayed MS, Vahedian-Azimi A, Mirmomeni G, Rahimi-Bashar F, Goharimoghadam K, Pourhoseingholi MA, Abbasi-Farajzadeh M, Hekmat M, Sathyapalan T, Guest PC, Sahebkar A. Depression, Anxiety, and Stress Among Patients with COVID-19: A Cross-Sectional Study. Adv Exp Med Biol. 2021;1321:229-236. doi: 10.1007/978-3-030-59261-5_19. PMID: 33656727. | | |
|  | Moni *et al.* 2021 | No mental health 🡪 risk/outcome data |
| Moni MA, Lin PI, Quinn JMW, Eapen V. COVID-19 patient transcriptomic and genomic profiling reveals comorbidity interactions with psychiatric disorders. Transl Psychiatry. 2021 Mar 15;11(1):160. doi: 10.1038/s41398-020-01151-3. PMID: 33723208; PMCID: PMC7957287. | | |
|  | Morioka *et al.* 2023 | No mental health 🡪 risk/outcome data |
| Morioka S, Tsuzuki S, Maruki T, Terada M, Miyazato Y, Kutsuna S, Saito S, Shimanishi Y, Takahashi K, Sanada M, Ashida S, Akashi M, Kuge C, Osanai Y, Tanaka K, Suzuki M, Hayakawa K, Ohmagari N. Epidemiology of post-COVID conditions beyond 1 year: a cross-sectional study. Public Health. 2023  Mar;216:39-44. doi: 10.1016/j.puhe.2023.01.008. Epub 2023 Feb 13. PMID:36791649. | | |
|  | Morlock *et al.* 2021 | No mental health 🡪 risk/outcome data |
| Morlock R, Morlock A, Downen M, Shah SN. COVID-19 prevalence and predictors in United States adults during peak stay-at-home orders. PLoS One. 2021 Jan 22;16(1):e0245586. doi: 10.1371/journal.pone.0245586. PMID: 33481900; PMCID: PMC7822538 | | |
|  | Mousavi 2020 | Opinion paper |
| Mousavi SB. Coronavirus disease 2019 pandemic: Do not forget patients with severe mental illness. Int J Soc Psychiatry. 2020 Jul 7:20764020939982. doi: 10.1177/0020764020939982. Epub ahead of print. PMID: 32633183. | | |
|  | Muhammad *et al.* 2021 | No mental health 🡪 risk/outcome data |
| Muhammad JS, Siddiqui R, Khan NA. COVID-19: Is There a Link between Alcohol Abuse and SARS-CoV-2-Induced Severe Neurological Manifestations? ACS Pharmacol Transl Sci. 2021 Mar 19;4(2):1024-1025. doi: 10.1021/acsptsci.1c00073. PMID: 33860221; PMCID: PMC8033744. | | |
|  | Mukhatar and Rana, 2020 | No comparison group |
| Mukhtar S, Rana W (2020). COVID-19 and individuals with mental illness in psychiatric facilities. *Psychiatry Res, 289*, 113075. doi:10.1016/j.psychres.2020.113075 | | |
|  | Munblit *et al.* 2021 | No mental health 🡪 risk/outcome data |
| Munblit D, Bobkova P, Spiridonova E, Shikhaleva A, Gamirova A, Blyuss O, et al. Incidence and risk factors for persistent symptoms in adults previously hospitalized for COVID‐19. Clinical and experimental allergy. 2021;51(9):1107–20. | | |
|  | Munch *et al.* 2022 | No mental health 🡪 risk/outcome data |
| Munch PK, Espenhain L, Hansen CH, Krause TG, Ethelberg S. Case-control study of activities associated with SARS-CoV-2 infection in an adult unvaccinated population and overview of societal COVID-19 epidemic counter measures in Denmark. PLoS One. 2022 Nov 16;17(11):e0268849. doi:  10.1371/journal.pone.0268849. PMID: 36383627; PMCID: PMC9668151. | | |
|  | Murga *et al.* 2021 | No mental health 🡪 risk/outcome data |
| Murga I, Aranburu L, Gargiulo PA, Gómez Esteban JC, Lafuente JV. Clinical Heterogeneity in ME/CFS. A Way to Understand Long-COVID19 Fatigue. Frontiers in psychiatry. 2021;12:735784–735784. | | |
|  | Murk *et al.* 2021 | Mental health 🡪 course, overlap |
| Murk W, Gierada M, Fralick M, Weckstein A, Klesh R, Rassen JA. Diagnosis-wide analysis of COVID-19 complications: an exposure-crossover study. CMAJ. 2021 Jan 4;193(1):E10-E18. doi: 10.1503/cmaj.201686. Epub 2020 Dec 7. PMID: 33293424; PMCID: PMC7774475. | | |
|  | Murphy *et al.* 2022 | No full-text access |
| Murphy KA, McGinty EE, Daumit GL. Hospitalization, Mechanical Ventilation, and Mortality After COVID-19 Among Adults With or Without Serious Mental Illness. Psychiatric services (Washington, DC). 2022;73(3):335–8. | | |
|  | Muruganandam *et al.* 2020 | No mental health 🡪 risk/outcome data |
| Muruganandam P, Neelamegam S, Menon V, Alexander J, Chaturvedi SK. COVID-19 and Severe Mental Illness: Impact on patients and its relation with their awareness about COVID-19. Psychiatry Res. 2020 Sep;291:113265. doi: 10.1016/j.psychres.2020.113265. Epub 2020 Jun 29. PMID: 32763536; PMCID: PMC7322460. | | |
|  | Musheyev *et al.* 2021 | Mental health 🡪 risk/outcome |
| Musheyev B, Janowicz R, Borg L, Matarlo M, Boyle H, Hou W, et al. Characterizing non-critically ill COVID-19 survivors with and without in-hospital rehabilitation. Scientific reports. 2021;11(1):21039–21039. | | |
|  | Narayan *et al.* 2021 | No mental health 🡪 risk/outcome data |
| Narayan A, Balkrishnan R. A health crisis within a health crisis: Opioid access in the COVID-19 pandemic. Subst Abus. 2021 Apr 13:1-5. doi: 10.1080/08897077.2021.1900981. Epub ahead of print. PMID: 33849399. | | |
|  | Nehme *et al.* 2022 | Mental health 🡪 risk/outcome – self report |
| Nehme M, Braillard O, Chappuis F, Courvoisier DS, Kaiser L, Soccal PM, et al. One‐year persistent symptoms and functional impairment in SARS‐CoV‐2 positive and negative individuals. Journal of internal medicine. 2022;292(1):103–15. | | |
|  | Nemani *et al.* 2021a | Mental health 🡪 mortality |
| Nemani K, Li C, Olfson M, Blessing EM, Razavian N, Chen J, Petkova E, Goff DC. Association of Psychiatric Disorders With Mortality Among Patients With COVID-19. JAMA Psychiatry. 2021 Apr 1;78(4):380-386. doi: 10.1001/jamapsychiatry.2020.4442. PMID: 33502436; PMCID: PMC7841576. | | |
|  | Nemani *et al.* 2021b | Mental health 🡪 risk/outcome – research letter |
| Nemani K, Conderino S, Marx J, Thorpe LE, Goff DC. Association Between Antipsychotic Use and COVID-19 Mortality Among People With Serious Mental Illness. Archives of general psychiatry. 2021;78(12):1391–3 | | |
|  | Nemani *et al.* 2022 | No mental health 🡪 risk/outcome data |
| Nemani K, Williams SZ, Olfson M, Leckman-Westin E, Finnerty M, Kammer J, et al. Association Between the Use of Psychotropic Medications and the Risk of COVID-19 Infection Among Long-term Inpatients With Serious Mental Illness in a New York State-wide Psychiatric Hospital System. JAMA network open. 2022;5(5):e2210743–e2210743. | | |
|  | Neuman Podczaska *et al.* 2020 | No mental health 🡪 risk/outcome data |
| Neumann-Podczaska A, Chojnicki M, Karbowski LM, Al-Saad SR, Hashmi AA, Chudek J, Tobis S, Kropinska S, Mozer-Lisewska I, Suwalska A, Tykarski A, Wieczorowska-Tobis K. Clinical Characteristics and Survival Analysis in a Small Sample of Older COVID-19 Patients with Defined 60-Day Outcome. Int J Environ Res Public Health. 2020 Nov 12;17(22):8362. doi: 10.3390/ijerph17228362. PMID: 33198124; PMCID: PMC7698090. | | |
|  | Neville *et al.* 2022 | No mental health 🡪 risk/outcome data |
| Neville TH, Hays RD, Tseng CH, Gonzalez CA, Chen L, Hong A, et al. Survival After Severe COVID-19: Long-Term Outcomes of Patients Admitted to an Intensive Care Unit. Journal of intensive care medicine. 2022;37(8):1019–28. | | |
|  | Ngepah 2021 | No mental health 🡪 risk/outcome data |
| Ngepah N. Socio-economic determinants of global COVID-19 mortalities: policy lessons for current and future pandemics. Health Policy Plan. 2021 Jan 13:czaa161. doi: 10.1093/heapol/czaa161. Epub ahead of print. PMID: 33439993; PMCID: PMC7928932. | | |
|  | Nguyen *et al.* 2020 | No mental health 🡪 risk/outcome data |
| Nguyen HC, Nguyen MH, Do BN, Tran CQ, Nguyen TTP, Pham KM, Pham LV, Tran KV, Duong TT, Tran TV, Duong TH, Nguyen TT, Nguyen QH, Hoang TM, Nguyen KT, Pham TTM, Yang SH, Chao JC, Duong TV. People with Suspected COVID-19 Symptoms Were More Likely Depressed and Had Lower Health-Related Quality of Life: The Potential Benefit of Health Literacy. J Clin Med. 2020 Mar 31;9(4):965. doi: 10.3390/jcm9040965. PMID: 32244415; PMCID: PMC7231234. | | |
|  | Nilsson *et al.* 2022 | Mental health 🡪 risk/outcome |
| Nilsson SF, Laursen TM, Osler M, Hjorthøj C, Benros ME, Ethelberg S, Mølbak K, Nordentoft M. Adverse SARS-CoV-2-associated outcomes among people experiencing social marginalisation and psychiatric vulnerability: A population-based cohort study among 4,4 million people. Lancet Reg Health Eur. 2022 Sep;20:100421. doi: 10.1016/j.lanepe.2022.100421. Epub 2022 Jun 30. PMID:35789954; PMCID: PMC9242846. | | |
|  | Nishimi *et al.* 2021 | Mental health 🡪 risk/outcome |
| Nishimi K, Neylan TC, Bertenthal D, Seal KH, O'Donovan A. Association of Psychiatric Disorders With Incidence of SARS-CoV-2 Breakthrough Infection Among Vaccinated Adults. JAMA network open. 2022;5(4):e227287–e227287. | | |
|  | Novak *et al.* 2021 | No mental health 🡪 risk/outcome data |
| Novak P, Sanmartin MX, Ali MM, Chen J. Health Conditions Associated With Severe Illness From COVID-19 Among Individuals With Serious Mental Illness. Psychiatr Serv. 2021 Apr 1;72(4):468-469. doi: 10.1176/appi.ps.202000300. Epub 2020 Nov 19. PMID: 33208029. | | |
|  | Ocsovszky *et al.* 2022 | No usable mental health 🡪 risk/outcome data |
| Ocsovszky Z, Otohal J, Berényi B, Juhász V, Skoda R, Bokor L, et al. The associations of long-COVID symptoms, clinical characteristics and affective psychological constructs in a non-hospitalized cohort. Physiology international. 2022;109(2):230–45. | | |
|  | Ohlis *et al.* 2022 | Mental health 🡪 risk/outcome / no mentally healthy control group |
| Ohlis A, Sörberg Wallin A, Sarafis A, Sjöqvist H, MacCabe JH, Ahlen J, et al. Clozapine treatment and risk of severe COVID‐19 infection. Acta psychiatrica Scandinavica. 2022;145(1):79–85. | | |
|  | Okubo *et al.* 2021 | No mental health 🡪 risk/outcome data |
| Okubo R, Yoshioka T, Nakaya T, Hanibuchi T, Okano H, Ikezawa S, Tsuno K, Murayama H, Tabuchi T. Urbanization level and neighborhood deprivation, not COVID-19 case numbers by residence area, are associated with severe psychological distress and new-onset suicidal ideation during the COVID-19 pandemic. J Affect Disord. 2021 May 15;287:89-95. doi: 10.1016/j.jad.2021.03.028. Epub 2021 Mar 15. PMID: 33774320. | | |
|  | Orlando *et al.* 2021 | Mental health 🡪 risk and course |
| Orlando V, Rea F, Savaré L, Guarino I, Mucherino S, Perrella A, Trama U, Coscioni E, Menditto E, Corrao G. Development and validation of a clinical risk score to predict the risk of SARS-CoV-2 infection from administrative data: A population-based cohort study from Italy. PLoS One. 2021 Jan 20;16(1):e0237202. doi: 10.1371/journal.pone.0237202. PMID: 33471809; PMCID: PMC7816996. | | |
|  | Park and Rhim, 2021 | No mental health 🡪 risk/outcome data |
| Park J, Rhim HC (2021). Association between mental illness and COVID-19 in South Korea. *Lancet Psychiatry,*  *8*(4), 270. doi:10.1016/s2215-0366(20)30465-x | | |
|  | Pavarin *et al.* 2022 | Mental health 🡪 risk/outcome |
| Pavarin RM, Fabbri C, De Ronchi D. COVID-19 hospitalization rates in individuals with substance or alcohol use disorders. Psychiatry research. 2022;311:114521–114521. | | |
|  | Peckham *et al.* 2021 | No mental health 🡪 risk/outcome data |
| Peckham E, Allgar V, Crosland S, Heron P, Johnston G, Newbronner E, Ratschen E, Spanakis P, Wadman R, Walker L, Gilbody S. Investigating smoking and nicotine dependence among people with severe mental illness during the COVID-19 pandemic: analysis of linked data from a UK Closing the Gap cohort. BJPsych Open. 2021 Apr 23;7(3):e86. doi: 10.1192/bjo.2021.45. PMID: 33888178; PMCID: PMC8082119. | | |
|  | Perera *et al.* 2021 | No mental health 🡪 risk/outcome data |
| Perera G, Mueller C, Broadbent M, Stewart R, Velayudhan L (2021). Mortality among mental health services  for older adults during the COVID-19 pandemic: a retrospective analysis from South London. *Int*  *Psychogeriatr, 33*(5), 527-528. doi:10.1017/s1041610221000442 | | |
|  | Pérez-Segura *et al.* 2021 | No usable mental health 🡪 risk/outcome data |
| Pérez-Segura P, Paz-Cabezas M, Núñez-Gil IJ, Arroyo-Espliguero R, Maroun Eid C, Romero R, et al. Prognostic factors at admission on patients with cancer and COVID-19: Analysis of HOPE registry data. Medicina clinica. 2021;157(7):318–24. | | |
|  | Perrot *et al.* 2022 | No usable mental health 🡪 risk/outcome data |
| Perrot C, Lebrun-Givois C, Edjolo A, Rouch I, Laurent B, Herrmann M, Dorey JM, Célarier T, Buisson A, Pongan É. Anxiété, dépression et évolution du coping durant la pandémie de Covid-19 dans une population de patients âgés avec troubles psychiatriques [Anxiety, depression and coping evolution during the Covid-19 pandemic in older people with psychiatric diseases]. Geriatr Psychol Neuropsychiatr Vieil. 2022 Dec 1;20(4):537-546. French. doi:10.1684/pnv.2022.1074. PMID: 36700446. | | |
|  | Piumatti *et al.* 2022 | No usable mental health 🡪 risk/outcome data |
| Piumatti G, Amati R, Richard A, Baysson H, Purgato M, Guessous I, Stringhini S, Albanese E; SEROCoV-POP; Specchio-Covid Study Group, The Corona Immunitas Ticino Working Group. Associations between Depression and Self-Reported COVID-19 Symptoms among Adults: Results from Two Population-Based Seroprevalence Studies in Switzerland. Int J Environ Res Public Health. 2022 Dec 12;19(24):16696. doi:  10.3390/ijerph192416696. PMID: 36554578; PMCID: PMC9779289. | | |
|  | Pizzonia *et al.* 2021 | No mental health 🡪 risk/outcome data |
| Pizzonia KL, Koscinski B, Suhr JA, Accorso C, Allan DM, Allan NP. Insomnia during the COVID-19 pandemic: the role of depression and COVID-19-related risk factors. Cogn Behav Ther. 2021 Mar 31:1-15. doi: 10.1080/16506073.2021.1879241. Epub ahead of print. PMID: 33787448. | | |
|  | Poblador-Plou *et al.* 2021 | Mental health 🡪 course |
| Poblador-Plou B, Carmona-Pírez J, Ioakeim-Skoufa I, Poncel-Falcó A, Bliek-Bueno K, Cano-Del Pozo M, Gimeno-Feliú LA, González-Rubio F, Aza-Pascual-Salcedo M, Bandrés-Liso AC, Díez-Manglano J, Marta-Moreno J, Mucherino S, Gimeno-Miguel A, Prados-Torres A, EpiChron Group. Baseline Chronic Comorbidity and Mortality in Laboratory-Confirmed COVID-19 Cases: Results from the PRECOVID Study in Spain. Int J Environ Res Public Health. 2020 Jul 17;17(14):5171. doi:10.3390/ijerph17145171. PMID: 32709002; PMCID: PMC7400393. | | |
|  | Posso *et al.* 2021 | No mental health 🡪 risk/outcome data |
| Posso M, Comas M, Román M, Domingo L, Louro J, González C, Castells X. (2020). Comorbidities and Mortality in Patients With COVID-19 Aged 60 Years and Older in a University Hospital in Spain. *Arch Bronconeumol (Engl Ed), 56*(11), 756-758. doi:10.1016/j.arbr.2020.06.010 | | |
|  | Qeadan *et al.* 2021 | Mental health 🡪 risk/outcome |
| Qeadan F, VanSant-Webb E, Tingey B, Rogers TN, Brooks E, Mensah NA, Winkfield KM, Saeed AI, English K, Rogers CR. Racial disparities in COVID-19 outcomes exist despite comparable Elixhauser comorbidity indices between Blacks, Hispanics, Native Americans, and Whites. Sci Rep. 2021 Apr 22;11(1):8738. doi: 10.1038/s41598-021-88308-2. PMID: 33888833; PMCID: PMC8062526. | | |
|  | Qi *et al.* 2020 | No mental health 🡪 risk/outcome data |
| Qi X, Wang J, Li X, Wang Z, Liu Y, Yang H, Li X, Shi J, Xiang H, Liu T, Kawada N, Maruyama H, Jiang Z, Wang F, Takehara T, Rockey DC, Sarin SK; COVID-Cirrhosis-CHESS Group. Clinical course of COVID-19 in patients with pre-existing decompensated cirrhosis: initial report from China. Hepatol Int. 2020 Jul;14(4):478-482. doi: 10.1007/s12072-020-10051-z. Epub 2020 May 22. PMID: 32440857; PMCID: PMC7242176. Y | | |
|  | Qiao *et al.* 2022 | Mental health 🡪 risk/outcome |
| Qiao S, Zhang J, Chen S, Olatosi B, Hardeman S, Narasimhan M, et al. How Different Pre-existing Mental Disorders and Their Co-occurrence Affects COVID-19 Clinical Outcomes? A Real-World Data Study in the Southern United States. Frontiers in public health. 2022;10:831189–831189. | | |
|  | Rajkumar 2021a | No mental health 🡪 risk/outcome data |
| Rajkumar RP. Is There a Relationship Between ADHD and COVID-19 Prevalence and Mortality Indices? An Analysis of Data From 156 Countries. Journal of attention disorders. 2022;26(8):1069–77. | | |
|  | Rajkumar 2021b | Mental health 🡪 risk/outcome |
| Rajkumar RP. The relationship between pre-COVID prevalence of common mental disorders and the impact of COVID-19. Minerva Psychiatry. 2021;62(3). | | |
|  | Rajkumar 2022 | Mental health 🡪 risk/outcome |
| Rajkumar, R. P. (2021). Cross-National Variations in COVID-19 Mortality: The Role of Diet, Obesity and Depression. *Diseases, 9*(2). doi:10.3390/diseases9020036 | | |
|  | Ramos-Rincon *et al.* 2021 | No mental health 🡪 risk/outcome data |
| Ramos-Rincon JM, Buonaiuto V, Ricci M, Martín-Carmona J, Paredes-Ruíz D, Calderón-Moreno M, Rubio-Rivas M, Beato-Pérez JL, Arnalich-Fernández F, Monge- Monge D, Vargas-Núñez JA, Acebes-Repiso G, Mendez-Bailon M, Perales-Fraile I, García-García GM, Guisado-Vasco P, Abdelhady-Kishta A, Pascual-Pérez MD, Rodríguez-Fernández-Viagas C, Montaño-Martínez A, López-Ruiz A, Gonzalez-Juarez MJ, Pérez-García C, Casas-Rojo JM, Gómez-Huelgas R; SEMI-COVID-19 Network. Clinical Characteristics and Risk Factors for Mortality in Very Old Patients Hospitalized With COVID-19 in Spain. J Gerontol A Biol Sci Med Sci. 2021 Feb 25;76(3):e28-e37. doi: 10.1093/gerona/glaa243. PMID: 33103720; PMCID:PMC7797762. | | |
|  | Ranger *et al.* 2023 | No mental health 🡪 risk/outcome data |
| Ranger TA, Clift AK, Patone M, Coupland CAC, Hatch R, Thomas K, Watkinson P, Hippisley-Cox J. Preexisting Neuropsychiatric Conditions and Associated Risk of Severe COVID-19 Infection and Other Acute Respiratory Infections. JAMA Psychiatry. 2023 Jan 1;80(1):57-65. doi: 10.1001/jamapsychiatry.2022.3614. PMID:  36350602; PMCID: PMC9647578. | | |
|  | Rauchman *et al.* 2021 | No mental health 🡪 risk/outcome data |
| Rauchman, S, Mendelson, S, Rauchman, C, Pinkhasov, A, Kasselman, L J, & Reiss, A B. Anti-Depressants And Covid-19 Severity: A Retrospective Study Of Hospitalized Adult Patients. 2022 Western Medical Research Conference *Journal of Investigative Medicine*2022;70**:**112-337. | | |
|  | Rebora *et al.* 2021 | No mental health 🡪 risk/outcome data |
| Rebora P, Rozzini R, Bianchetti A, Blangiardo P, Marchegiani A, Piazzoli A, . . . Bellelli G. (2021). Delirium in Patients with SARS-CoV-2 Infection: A Multicenter Study. *J Am Geriatr Soc, 69*(2), 293-299. doi:10.1111/jgs.16969 | | |
|  | Reilev *et al.* 2020 | Double data |
| Reilev M, Kristensen KB, Pottegård A, Lund LC, Hallas J, Ernst MT, Christiansen CF, Sørensen HT, Johansen NB, Brun NC, Voldstedlund M, Støvring H, Thomsen MK, Christensen S, Gubbels S, Krause TG, Mølbak K, Thomsen RW. Characteristics and predictors of hospitalization and mortality in the first 11 122 cases with a positive RT-PCR test for SARS-CoV-2 in Denmark: a nationwide cohort. Int J Epidemiol. 2020 Oct 1;49(5):1468-1481. doi: 10.1093/ije/dyaa140. PMID: 32887982; PMCID: PMC7499657. | | |
|  | Ren *et al.* 2020 | No mental health 🡪 risk/outcome data |
| Ren L, Yao D, Cui Z, Chen S, Yan H. Corona Virus Disease 2019 patients with different disease severity or age range: A single-center study of clinical features and prognosis. Medicine (Baltimore). 2020 Dec 4;99(49):e22899. doi: 10.1097/MD.0000000000022899. PMID: 33285678; PMCID: PMC7717834. | | |
|  | Righi *et al.* 2022 | No mental health 🡪 risk/outcome data |
| Righi E, Mirandola M, Mazzaferri F, Dossi G, Razzaboni E, Zaffagnini A, et al. Determinants of persistence of symptoms and impact on physical and mental wellbeing in Long COVID: A prospective cohort study. The Journal of infection. 2022;84(4):566–72. | | |
|  | Rivera-Izquirdo *et al.* 2020 | No mental health 🡪 risk/outcome data |
| Rivera-Izquierdo M, Del Carmen Valero-Ubierna M, R-delAmo JL, Fernández-García MÁ, Martínez-Diz S, Tahery-Mahmoud A, Rodríguez-Camacho M, Gámiz-Molina AB, Barba-Gyengo N, Gámez-Baeza P, Cabrero-Rodríguez C, Guirado-Ruiz PA, Martín- Romero DT, Láinez-Ramos-Bossini AJ, Sánchez-Pérez MR, Mancera-Romero J, García- Martín M, Martín-delosReyes LM, Martínez-Ruiz V, Lardelli-Claret P, Jiménez- Mejías E. Sociodemographic, clinical and laboratory factors on admission associated with COVID-19 mortality in hospitalized patients: A retrospective observational study. PLoS One. 2020 Jun 25;15(6):e0235107. doi: 10.1371/journal.pone.0235107. PMID: 32584868; PMCID: PMC7316360. y | | |
|  | Rivera-Izquierdo *et al.* 2022 | No mental health 🡪 risk/outcome data |
| Rivera-Izquierdo M, Láinez-Ramos-Bossini AJ, de Alba IGF, Ortiz-González-Serna R, Serrano-Ortiz Álvaro, Fernández-Martínez NF, et al. Long COVID 12 months after discharge: persistent symptoms in patients hospitalised due to COVID-19 and patients hospitalised due to other causes-a multicentre cohort study. BMC medicine. 2022;20(1):92–92. | | |
|  | Rodriguez *et al.* 2021 | No mental health 🡪 risk/outcome data |
| Rodriguez CV, Horcajadas FA, Arroba CMA, Ripoll CC, Gonzalez AJ, Pajares ME, . . . Rubio G. (2021). COVID-  19-Related Neuropsychiatric Symptoms in Patients With Alcohol Abuse Conditions During the SARS-CoV-2  Pandemic: A Retrospective Cohort Study Using Real World Data From Electronic Health Records of a Tertiary  Hospital. *Front Neurol, 12*. doi:10.3389/fneur.2021.630566 | | |
|  | Rodríguez-Molinero *et al.* 2020 | Mental health 🡪 course |
| Rodríguez-Molinero A, Gálvez-Barrón C, Miñarro A, Macho O, López GF, Robles MT, Dapena MD, Martínez S,  Milà Ràfols N, Monaco EE, Hidalgo García A; COVID-19 Research Group of CSAPG. Association between COVID-  19 prognosis and disease presentation, comorbidities and chronic treatment of hospitalized patients. PLoS  One. 2020 Oct 15;15(10):e0239571. doi: 10.1371/journal.pone.0239571. PMID: 33057443; PMCID:  PMC7561079. | | |
|  | Roessler *et al.* 2022 | No mental health 🡪 risk/outcome data |
| Roessler M, Tesch F, Batram M, Jacob J, Loser F, Weidinger O, Wende D, Vivirito A, Toepfner N, Ehm F, Seifert M, Nagel O, König C, Jucknewitz R, Armann JP, Berner R, Treskova-Schwarzbach M, Hertle D, Scholz S, Stern S, Ballesteros P, Baßler S, Bertele B, Repschläger U, Richter N, Riederer C, Sobik F, Schramm A, Schulte C, Wieler L, Walker J, Scheidt-Nave C, Schmitt J. Post- COVID-19-associated morbidity in children, adolescents, and adults: A matched cohort study including more than 157,000 individuals with COVID-19 in Germany. PLoS Med. 2022 Nov 10;19(11):e1004122. doi: 10.1371/journal.pmed.1004122. PMID: 36355754; PMCID: PMC9648706. | | |
|  | Roig *et al.* 2021 | No mental health 🡪 risk/outcome data |
| Roig SO, Soler-Blanco N, Jimenez IT, Otero EV, Moreno-Arino M, Gomez-Valent M. (2021). Clinical and pharmacological data in COVID-19 hospitalized nonagenarian patients. *Revista Espanola De Quimioterapia, 34*(2), 145-150. doi:10.37201/req/130.2020 | | |
|  | Romero-Duarte *et al.* 2022 | Mental health 🡪 risk/outcome |
| Romero Duarte Á, Rivera Izquierdo M, Láinez Ramos-Bossini AJ, Redruello Guerrero P & Cárdenas Cruz A Factors associated with readmission to the Emergency Department in a cohort of COVID-19 hospitalized patients. Signa Vitae. 2022. 18(1);47-54. | | |
|  | Rosoff *et al.* 2021 | No mental health 🡪 risk/outcome data |
| Rosoff DB, Yoo J, Lohoff F. W. (2021). A genetically-informed study disentangling the relationships between tobacco smoking, cannabis use, alcohol consumption, substance use disorders and respiratory infections, including COVID-19. *medRxiv*. doi:10.1101/2021.02.11.21251581 | | |
|  | Rubin 2020 | Opinion paper |
| Rubin R. Substance Use Disorder Linked to Higher COVID-19 Risk. JAMA. 2020 Oct 27; 324(16):1598. doi: 10.1001/jama.2020.19686. PMID: 33107926. | | |
|  | Saengow *et al.* 2021 | Opinion paper |
| Saengow U, Assanangkornchai S, Casswell S. Alcohol: a probable risk factor of COVID-19 severity. Addiction. 2021 Jan;116(1):204-205. doi: 10.1111/add.15194. Epub 2020 Aug 12. PMID: 32688440; PMCID: PMC7404678. | | |
|  | Said *et al.* 2021 | No mental health 🡪 risk/outcome data |
| Said DS, Lopes G, Lorettu L, Farina G, Napodano CMP, Amadori A, Pichierri G, Cegolon L, Padrini S, Bellizzi S, Alzoubi Y. Mental health and COVID-19 pandemics: The worrisome humanitarian perspective from the Middle East. J Glob Health. 2021 Jan 30;11:03014. doi: 10.7189/jogh.11.03014. PMID: 33643624; PMCID: PMC7898376. | | |
|  | Saldi *et al.* 2021 | No mental health 🡪 risk/outcome data |
| Saldi SRF, Safitri ED, Setiati S, Ranakusuma RW, Marsigit J, Azwar MK, et al. Prognostic Scoring System for Mortality of Hospitalized COVID-19 Patients in Resource-Limited Settings: A Multicenter Study from COVID-19 Referral Hospitals. Acta medica Indonesiana. 2021;53(4):407–15. | | |
|  | Salim 2021 | Opinion paper |
| Salim S. The Stress of the COVID-19 Pandemic: Beyond the Data. Curr Neuropharmacol. 2021 Mar 10. doi: 10.2174/1570159X19666210311103136. Epub ahead of print. PMID: 33719975. | | |
|  | Salinas-Botrán *et al.* 2021 | Mental health 🡪 risk/outcome |
| Salinas-Botrán A, Sanz-Cánovas J, Pérez-Somarriba J, Pérez-Belmonte LM, Cobos-Palacios L, Rubio-Rivas M, et al. Clinical characteristics and risk factors for mortality upon admission in patients with heart failure hospitalized due to COVID-19 in Spain. Revista clínica espanõla. 2022;222(5):255–65. | | |
|  | Salvatore *et al.* 2021 | Mental health 🡪 course |
| Salvatore M, Gu T, Mack JA, Sankar SP, Patil S, Valley TS, . . . Mukherjeea B (2021). A Phenome-Wide Association Study (PheWAS) of COVID-19 Outcomes by Race Using the Electronic Health Records Data in Michigan Medicine. *J Clin Med, 10*(7). doi:10.3390/jcm10071351 | | |
|  | Sami *et al.* 2020 | No mental health 🡪 risk/outcome data |
| Sami R, Soltaninejad F, Amra B, Naderi Z, Haghjooy Javanmard S, Iraj B, Haji Ahmadi S, Shayganfar A, Dehghan M, Khademi N, Sadat Hosseini N, Mortazavi M, Mansourian M, Mañanas MA, Marateb HR, Adibi P. A one-year hospital-based prospective COVID-19 open-cohort in the Eastern Mediterranean region: The Khorshid COVID Cohort (KCC) study. PLoS One. 2020 Nov 5;15(11):e0241537. doi: 10.1371/journal.pone.0241537. PMID: 33151983; PMCID: PMC7644058. | | |
|  | Saurabh *et al.* 2021 | No mental health 🡪 risk/outcome data |
| Saurabh S, Verma MK, Gautam V, Kumar N, Jain V, Goel AD, Gupta MK, Sharma PP, Bhardwaj P, Singh K, Nag VL, Garg MK, Misra S. Tobacco, alcohol use and other risk factors for developing symptomatic COVID-19 vs asymptomatic SARS-CoV-2 infection: a case-control study from western Rajasthan, India. Trans R Soc Trop Med Hyg. 2021 Jan 14:traa172. doi: 10.1093/trstmh/traa172. | | |
|  | Schieber *et al.* 2023 | Mix disorders and SUDS 🡪 hospitalization but overlap in data |
| Schieber LZ, Dunphy C, Schieber RA, Lopes-Cardozo B, Moonesinghe R, Guy GP Jr. Hospitalization Associated With Comorbid Psychiatric and Substance Use Disorders Among Adults With COVID-19 Treated in US Emergency Departments From April 2020 to August 2021. JAMA Psychiatry. 2023 Apr 1;80(4):331-341. doi:  10.1001/jamapsychiatry.2022.5047. | | |
|  | Schott *et al.* 2023 | No mental health 🡪 risk/outcome data |
| Schott W, Tao S, Shea L. Prevalence of high-risk conditions for severe COVID-19 among Medicaid-enrolled children with autism and mental health diagnoses in the United States. Autism. 2023 Feb 17:13623613231155265. doi: 10.1177/13623613231155265. | | |
|  | Segaloff *et al.* 2021 | No mental health 🡪 risk/outcome data |
| Segaloff HE, Cole D, Rosenblum HG, Lee CC, Morgan CN, Remington P, et al. Risk Factors for Severe Acute Respiratory Syndrome Coronavirus 2 (SARS-CoV-2) Infection and Presence of Anti–SARS-CoV-2 Antibodies Among University Student Dormitory Residents, September–November 2020. Open forum infectious diseases. 2021;8(9):ofab405–ofab405. | | |
|  | Shafran *et al.* 2021 | No mental health 🡪 risk/outcome data |
| Shafran R, Rachman S, Whittal M, Radomsky A, Coughtrey A. Fear and Anxiety in COVID-19: Preexisting Anxiety Disorders. Cogn Behav Pract. 2021 Apr 19. doi: 10.1016/j.cbpra.2021.03.003. | | |
|  | Shanbehzadeh *et al.* 2023 | No mental health 🡪 risk/outcome data |
| Shanbehzadeh S, Zanjari N, Yassin M, Yassin Z, Tavahomi M. Association between long COVID, functional activity, and health-related quality of life in older adults. BMC Geriatr. 2023 Jan 23;23(1):40. doi:  10.1186/s12877-023-03757-w. | | |
|  | Shang *et al.* 2020 | No mental health 🡪 risk/outcome data |
| Shang Y, Xu C, Jiang F, Huang R, Li Y, Zhou Y, Xu F, Dai H. Clinical characteristics and changes of chest CT features in 307 patients with common COVID-19 pneumonia infected SARS-CoV-2: A multicenter study in Jiangsu, China. Int J Infect Dis. 2020 Jul;96:157-162. doi: 10.1016/j.ijid.2020.05.006. | | |
|  | Shi *et al.* 2020 | No mental health 🡪 risk/outcome data |
| Shi SM, Bakaev I, Chen HL, Travison TG, Berry SD. (2020). Risk Factors, Presentation, and Course of Coronavirus Disease 2019 in a Large, Academic Long-Term Care Facility. *J Am Med Dir Assoc, 21*(10), 1378-+. doi:10.1016/j.jamda.2020.08.027 | | |
|  | Shrivastava *et al.* 2020 | Opinion paper |
| Shrivastava SR, Shrivastava PS. COVID-19 and Alcohol Consumption: No Preventive or Therapeutic Benefits. Int J Prev Med. 2020 Jul 9;11:91. doi: 10.4103/ijpvm.IJPVM_198_20. | | |
|  | Siddiqui *et al.* 2023 | SUDS No mental health 🡪 risk/outcome data |
| Siddiqui S, Kelly L, Bosch N, Law A, Patel LA, Perkins N, Armaignac DL, Zabolotskikh I, Christie A, Krishna Mohan S, Deo N, Bansal V, Kumar VK, Gajic O, Kashyap R, Domecq JP, Boman K, Walkey A, Banner-Goodspeed V, Schaefer MS. Discharge Disposition and Loss of Independence Among Survivors of COVID-19  Admitted to Intensive Care: Results From the SCCM Discovery Viral Infection and Respiratory Illness Universal Study (VIRUS). J Intensive Care Med. 2023 May 8:8850666231174375. doi: 10.1177/08850666231174375. | | |
|  | Simon *et al.* 2020 | Opinion paper |
| Simon NM, Saxe GN, Marmar CR. Mental Health Disorders Related to COVID-19-Related Mortalitys. JAMA. 2020 Oct 20;324(15):1493-1494. doi:10.1001/jama.2020.19632. PMID: 33044510. | | |
|  | Simons *et al.* 2020 | Meta-analysis smoking 🡪 COVID-19 risk |
| Simons D, Shahab L, Brown J, Perski O. The association of smoking status with SARS-CoV-2 infection, hospitalization and mortality from COVID-19: a living rapid evidence review with Bayesian meta-analyses (version 7). Addiction. 2021 Jun;116(6):1319-1368. doi: 10.1111/add.15276. Epub 2020 Nov 17. PMID: 33007104; PMCID: PMC7590402. | | |
|  | Singh *et al.* 2021 [MEDRXIV] | No mental health 🡪 risk/outcome data |
| Singh, R., Rathore, S. S., Khan, H., Bhurwal, A., Sheraton, M., Ghosh, P., ... & Bansal, V. (2021). Mortality and Severity in COVID-19 Patients on ACEIs & ARBs-A Meta-Regression Analysis. *medRxiv*. | | |
|  | Sisó-Almirall *et al.* 2020 | Mental health 🡪 course |
| Sisó-Almirall A, Kostov B, Mas-Heredia M, Vilanova-Rotllan S, Sequeira-Aymar E, Sans-Corrales M, Sant-Arderiu E, Cayuelas-Redondo L, Martínez-Pérez A, García-Plana N, Anguita-Guimet A, Benavent-Àreu J. Prognostic factors in Spanish COVID-19 patients: A case series from Barcelona. PLoS One. 2020 Aug 21;15(8):e0237960. doi: 10.1371/journal.pone.0237960. PMID: 32822413; PMCID: PMC7444503. | | |
|  | Siva 2021 | Opinion paper |
| Siva N. Severe mental illness: reassessing COVID-19 vaccine priorities. Lancet. 2021 Feb 20;397(10275):657. doi: 10.1016/S0140-6736(21)00429-3. PMID: 33610199; PMCID: PMC7906718. | | |
|  | Slaunwhite *et al.* 2020 | No mental health 🡪 risk/outcome data |
| Slaunwhite, A. K., Gan, W. Q., Xavier, C., Zhao, B., Buxton, J. A., & Desai, R. (2020). Overdose and risk  factors for coronavirus disease 2019. *Drug Alcohol Depend, 212*. doi:10.1016/j.drugalcdep.2020.108047 | | |
|  | Sobotka *et al.* 2023 | No mental health 🡪 risk/outcome data |
| Sobotka LA, Jain A, Peng J, Allen KD, McShane CJ, Ramsey ML, Wellner MR, Kirkpatrick RB. Patients with alcohol-related liver disease hospitalized during the COVID-19 pandemic experienced worse outcomes. Ann Hepatol. 2023 May-Jun;28(3):101088. doi: 10.1016/j.aohep.2023.101088. Epub 2023 Mar 16. PMID:  36933885; PMCID: PMC10017381. | | |
|  | Sorg *et al.* 2022 | No mental health 🡪 risk/outcome data |
| Sorg AL, Becht S, Jank M, Armann J, von Both U, Hufnagel M, Lander F, Liese JG, Niehues T, Verjans E, Wetzke M, Stojanov S, Behrends U, Drosten C, Schroten H, von Kries R. Association of SARS-CoV-2 Seropositivity With Myalgic Encephalomyelitis and/or Chronic Fatigue Syndrome Among Children and Adolescents in Germany. JAMA Netw Open. 2022 Sep 1;5(9):e2233454. doi: 10.1001/jamanetworkopen. 2022.33454. PMID: 36166227; PMCID: PMC9516317. | | |
|  | Spagnolo *et al.* 2020 | Opinion paper |
| Spagnolo PA, Montemitro C, Leggio L. New Challenges in Addiction Medicine: COVID-19 Infection in Patients With Alcohol and Substance Use Disorders-The Perfect Storm. Am J Psychiatry. 2020 Sep 1;177(9):805-807. doi: 10.1176/appi.ajp.2020.20040417. Epub 2020 Jul 14. PMID: 32660296. | | |
|  | Stahlman *et al.* 2021 | Mental health 🡪 risk/outcome, No full-text access |
| Stahlman S, Hiban K, Mahaney H, Ford S. Incident COVID-19 Infections, Active and Reserve Components, 1 January 2020-31 August 2021. MSMR (US Army Center for Health Promotion and Preventive Medicine, Executive Communications Division). 2021;28(12):14–21. | | |
|  | Stanton *et al.* 2020 | No mental health 🡪 risk/outcome data |
| Stanton R, To QG, Khalesi S, Williams SL, Alley SJ, Thwaite TL, Fenning AS, Vandelanotte C. Depression, Anxiety and Stress during COVID-19: Associations with Changes in Physical Activity, Sleep, Tobacco and Alcohol Use in Australian Adults. Int J Environ Res Public Health. 2020 Jun 7;17(11):4065. doi: 10.3390/ijerph17114065. PMID: 32517294; PMCID: PMC7312903. | | |
|  | Susanto *et al.* 2022 | No mental health 🡪 risk/outcome data |
| Susanto AD, Isbaniah F, Pratomo IP, Antariksa B, Samoedro E, Taufik M, et al. Clinical characteristics and quality of life of persistent symptoms of COVID-19 syndrome in Indonesia. Germs (Bucureşti). 2022;12(2):158–68. | | |
|  | Schwarzinger *et al.* 2023 | Exclude overlap |
| Schwarzinger M, Luchini S, Teschl M, Alla F, Mallet V, Rehm J. Mental disorders, COVID-19-related life-saving measures and mortality in France: A nationwide cohort study. PLoS Med. 2023 Feb 6;20(2):e1004134. doi:  10.1371/journal.pmed.1004134. PMID: 36745669; PMCID: PMC10089350. | | |
|  | Swendson 2020 | Opinion paper |
| Swendsen J. COVID-19 and mental health: How one pandemic can reveal another. J Behav Cogn Ther. 2020 Sep;30(3):161-163. doi: 10.1016/j.jbct.2020.08.001. Epub 2020 Sep 15. PMID: 32954371; PMCID: PMC7492066. | | |
|  | Talhari *et al.* 2023 | No mental health 🡪 risk/outcome data |
| Talhari C, Criado PR, Castro CCS, Ianhez M, Ramos PM, Miot HA. Prevalence of and risk factors for post-COVID: Results from a survey of 6,958 patients from Brazil. An Acad Bras Cienc. 2023 Mar 24;95(1):e20220143. doi:10.1590/0001-3765202320220143. PMID: 36995792. | | |
|  | Tamburin *et al.* 2021 | No mental health 🡪 risk/outcome data |
| Tamburin S, Mantovani E, De Bernardis E, Zipeto D, Lugoboni F (2021). COVID-19 and related symptoms in  patients under disulfiram for alcohol use disorder. *Internal and Emergency Medicine*, 1-3.  doi:10.1007/s11739-021-02633-y | | |
|  | Tang *et al.* 2020 | Mental health 🡪 risk and course |
| Tang O, Bigelow BF, Sheikh F, Peters M, Zenilman JM, Bennett R, Katz MJ. Outcomes of Nursing Home COVID-19 Patients by Initial Symptoms and Comorbidity: Results of Universal Testing of 1970 Residents. J Am Med Dir Assoc. 2020 Dec;21(12):1767-1773.e1. doi: 10.1016/j.jamda.2020.10.011. Epub 2020 Oct 14. PMID: 33153910; PMCID: PMC7556822. | | |
|  | Taquet *et al.* 2021 a | No mental health 🡪 risk/outcome data |
| Taquet M, Geddes JR, Husain M, Luciano S, Harrison PJ. 6-month neurological and psychiatric outcomes in 236 379 survivors of COVID-19: a retrospective cohort study using electronic health records. Lancet Psychiatry. 2021 May;8(5):416-427. doi: 10.1016/S2215-0366(21)00084-5. Epub 2021 Apr 6. PMID: 33836148; PMCID: PMC8023694. | | |
|  | Taquet *et al.* 2021 b | Mental health 🡪 risk and course |
| Taquet M, Luciano S, Geddes JR, Harrison PJ. Bidirectional associations between COVID-19 and psychiatric disorder: retrospective cohort studies of 62 354 COVID-19 cases in the USA. Lancet Psychiatry. 2021 Feb;8(2):130-140. doi: 10.1016/S2215-0366(20)30462-4. Epub 2020 Nov 9. | | |
|  | Teixeira *et al.* 2021 | Mental health 🡪 risk |
| Teixeira AL, Krause TM, Ghosh L, Shahani L, Machado-Vieira R, Lane SD, et al. Analysis of COVID-19 Infection and Mortality Among Patients With Psychiatric Disorders, 2020. JAMA network open. 2021;4(11):e2134969–e2134969. | | |
|  | Testino 2020 | Opinion paper |
| Testino G. Are Patients With Alcohol Use Disorders at Increased Risk for Covid-19 Infection? Alcohol Alcohol. 2020 Jun 25;55(4):344-346. doi: 10.1093/alcalc/agaa037. PMID: 32400858; PMCID: PMC7239257. | | |
|  | Testino et al. 2022 | No mental health 🡪 risk/outcome data |
| Testino G, Pellicano R. Alcohol consumption: confirmed as cause of increased COVID-19 disease severity. Minerva Gastroenterology. 2022;68(2):232–3. | | |
|  | Thompson et al. 2022 | Mental health 🡪 long covid |
| Thompson EJ, Williams DM, Walker AJ, Mitchell RE, Niedzwiedz CL, Yang TC, Huggins CF, Kwong ASF, Silverwood RJ, Di Gessa G, Bowyer RCE, Northstone K, Hou B, Green MJ, Dodgeon B, Doores KJ, Duncan EL, Williams FMK; OpenSAFELY Collaborative, Steptoe A, Porteous DJ, McEachan RRC, Tomlinson L, Goldacre B, Patalay P, Ploubidis GB, Katikireddi SV, Tilling K, Rentsch CT, Timpson NJ, Chaturvedi N, Steves CJ. Long COVID burden and risk factors in 10 UK longitudinal studies and electronic health records. Nat Commun. 2022 Jun 28;13(1):3528. doi: 10.1038/s41467-022-30836-0. PMID: 35764621; PMCID: PMC9240035. | | |
|  | Thronicke et al. 2022 | No mental health 🡪 risk/outcome data |
| Thronicke A, Hinse M, Weinert S, Jakubowski A, Grieb G, Matthes H. Factors Associated with Self-Reported Post/Long-COVID-A Real-World Data Study. Int J Environ Res Public Health. 2022 Dec 2;19(23):16124. doi:  10.3390/ijerph192316124. PMID: 36498197; PMCID: PMC9738553. | | |
|  | Tobolowsky *et al.* 2021 | No mental health 🡪 risk/outcome data |
| Tobolowsky FA, Bardossy AC, Currie DW, Schwartz NG, Zacks RLT, Chow EJ, Dyal JW, Ali H, Kay M, Duchin JS, Brostrom-Smith C, Clark S, Sykes K, Jernigan JA, Honein MA, Clark TA, Stone ND, Reddy SC, Rao AK. Signs, Symptoms, and Comorbidities Associated With Onset and Prognosis of COVID-19 in a Nursing Home. J Am Med Dir Assoc. 2021 Mar;22(3):498-503. doi: 10.1016/j.jamda.2021.01.070. Epub 2021 Jan 28. PMID: 33549565; PMCID: PMC7843086. | | |
|  | Tokuda *et al.* 2023 | Serious mental illness 🡪 mortality |
| Tokuda Y, Barnett PB, Sanji S, Takaizumi Y, Tomono M, Tokuda H, Taniguchi K, Shibuya K. Serious mental illness and in-hospital mortality among hospitalized patients with acute COVID-19: A large-database analysis in Japan. Gen Hosp Psychiatry. 2023 May-Jun;82:1-6. doi: 10.1016/j.genhosppsych.2023.01.014. Epub  2023 Feb 3. PMID: 36868102; PMCID: PMC9894824. | | |
|  | Tsai *et al.* 2021 | No validated predictor variable |
| Tsai J, Huang M, Elbogen E. Mental Health and Psychosocial Characteristics Associated With COVID-19 Among U.S. Adults. Psychiatr Serv. 2021 Apr 1;72(4):444-447. doi: 10.1176/appi.ps.202000540. Epub 2021 Feb 3. PMID:33530731. | | |
|  | Tsigkas *et al.* 2021 | No mental health 🡪 risk/outcome data |
| Tsigkas G, Koufou EE, Katsanos K, Patrinos P, Moulias A, Miliordos I, Almpanis G, Christodoulou I, Papanikolaou F, Dimitroula T, Kivetos A, Vardas P, Davlouros P. Potential Relationship Between Lifestyle Changes and Incidence of Hospital Admissions for Acute Coronary Syndrome During the COVID-19 Lockdown. Front Cardiovasc Med. 2021 Feb 11;8:604374. doi: 10.3389/fcvm.2021.604374. PMID: 33644128; PMCID: PMC7904890. | | |
|  | Turk *et al.* 2020 | No mental health 🡪 risk/outcome data |
| Turk MA, Landes SD, Formica MK, Goss KD. Intellectual and developmental disability and COVID-19 case-fatality trends: TriNetX analysis. Disabil Health J. 2020 Jul;13(3):100942. doi: 10.1016/j.dhjo.2020.100942. Epub 2020 May 24. PMID: 32473875; PMCID: PMC7245650. | | |
|  | Tzur Bitan *et al.* 2021 | Mental health 🡪 risk and course |
| Tzur Bitan D, Kridin K, Cohen AD, Weinstein O. COVID-19 hospitalisation, mortality, vaccination, and postvaccination trends among people with schizophrenia in Israel: a longitudinal cohort study. The Lancet Psychiatry. 2021;8(10):901–8. | | |
|  | Tzur Bitan *et al.* 2022 | Mental health 🡪 booster vaccination rate |
| Tzur Bitan D, Kridin K, Givon-Lavi N, Krieger I, Kaliner E, Cohen AD, et al. COVID-19 Booster Vaccination Among Individuals With Schizophrenia in Israel. Archives of general psychiatry. 2022;79(5):508–12. | | |
|  | Vadukapuram *et al.* 2022 | Review article |
| Vadukapuram R, Trivedi C, Mansuri Z. Does a Mental Health Diagnosis Worsen Outcomes From COVID-19? Primary care companion for CNS disorders. 2022;24(1). | | |
|  | Vaish *et al.* 2022 |  |
| Vaish A, Ray S, Tyson B. A Study on the Correlations Between Comorbid Disease Conditions and Central and Peripheral Neurological Manifestations of COVID-19. Cureus. 2022 Oct 2;14(10):e29838. doi: 10.7759/cureus.29838. PMID: 36337781;PMCID: PMC9625534. | | |
|  | Valente *et al.* 2021 | No mental health 🡪 risk/outcome data |
| Valente JY, Sohi I, Garcia-Cerde R, Monteiro MG, Sanchez ZM. What is associated with the increased frequency of heavy episodic drinking during the COVID-19 pandemic? Data from the PAHO regional web-based survey. Drug Alcohol Depend. 2021 Apr 1;221:108621. doi: 10.1016/j.drugalcdep.2021.108621. Epub 2021 Feb 16. PMID: 33636598. | | |
|  | Vallecillo *et al.* 2021 | No mental health 🡪 risk/outcome data |
| Vallecillo G, Perelló R, Güerri R, Fonseca F, Torrens M (2021). Clinical impact of COVID-19 on people with  substance use disorders. *J Public Health (Oxf), 43*(1), 9-12. doi:10.1093/pubmed/fdaa181 | | |
|  | van der Meer *et al.* 2020 | Exclude; double data |
| van der Meer D, Pinzón-Espinosa J, Lin BD, Tijdink JK, Vinkers CH, Guloksuz S, Luykx JJ. Associations between  psychiatric disorders, COVID-19 testing probability and COVID-19 testing results: findings from a population-  based study. BJPsych Open. 2020 Jul 22;6(5):e87. doi:10.1192/bjo.2020.75. PMID: 32696734; PMCID:  PMC7417998. | | |
|  | van der Valk *et al.* 2021 | No mental health 🡪 risk/outcome data |
| van der Valk JPM, Heijboer FWJ, van Middendorp H, Evers AWM, In 't Veen JCCM. Case-control study of patient characteristics, knowledge of the COVID-19 disease, risk behaviour and mental state in patients visiting an emergency room with COVID-19 symptoms in the Netherlands. PLoS One. 2021 Apr 28;16(4):e0249847. doi: 10.1371/journal.pone.0249847. PMID: 33909639; PMCID: PMC8081234. | | |
|  | van Laar *et al.* 2020 | No mental health 🡪 risk/outcome data |
| van Laar MW, Oomen PE, van Miltenburg CJA, Vercoulen E, Freeman TP, Hall WD. Cannabis and COVID-19: Reasons for Concern. Front Psychiatry. 2020 Dec 21;11:601653. doi: 10.3389/fpsyt.2020.601653. PMID: 33408655; PMCID: PMC7779403. | | |
|  | Varela-Rodríguez *et al.* 2021 | Mental health 🡪 course |
| Varela Rodríguez C, Arias Horcajadas F, Martín-Arriscado Arroba C, Combarro Ripoll C, Juanes Gonzalez A, Esperesate Pajares M, Rodrigo Holgado I, Cadenas Manceñido Á, Sánchez Rodríguez L, Baselga Penalva B, Marín M, Rubio G. COVID-19-Related Neuropsychiatric Symptoms in Patients With Alcohol Abuse Conditions During the SARS-CoV-2 Pandemic: A Retrospective Cohort Study Using Real World Data From Electronic Health Records of a Tertiary Hospital. Front Neurol. 2021 Mar 3;12:630566. doi: 10.3389/fneur.2021.630566. PMID: 33746884;PMCID: PMC7966461 | | |
|  | Vedhara *et al.* 2022 | No mental health 🡪 risk/outcome data |
| Vedhara K, Ayling K, Jia R, Fairclough L, Morling JR, Ball JK, et al. Relationship Between Anxiety, Depression, and Susceptibility to Severe Acute Respiratory Syndrome Coronavirus 2 Infection: Proof of Concept. The Journal of infectious diseases. 2022;225(12):2137–41. | | |
|  | Velásquez García *et al.* 2021 | Mental health 🡪 risk/outcome |
| Velásquez García HA, Wilton J, Smolina K, Chong M, Rasali D, Otterstatter M, et al. Mental Health and Substance Use Associated with Hospitalization among People with COVID-19: A Population-Based Cohort Study. Viruses. 2021;13(11):2196. | | |
|  | Veldhuis *et al.* 2021 | No mental health 🡪 risk/outcome data |
| Veldhuis CB, Nesoff ED, McKowen ALW, Rice DR, Ghoneima H, Wootton AR, Papautsky EL, Arigo D, Goldberg S, Anderson JC. Addressing the critical need for long-term mental health data during the COVID-19 pandemic: Changes in mental health from April to September 2020. Prev Med. 2021 May;146:106465. doi:10.1016/j.ypmed.2021.106465. Epub 2021 Feb 27. PMID: 33647353. | | |
|  | Vena *et al.* 2020 | No mental health 🡪 risk/outcome data |
| Vena A, Giacobbe DR, Di Biagio A, Mikulska M, Taramasso L, De Maria A, Ball L, Brunetti I, Loconte M, Patroniti NA, Robba C, Delfino E, Dentone C, Magnasco L, Nicolini L, Toscanini F, Bavastro M, Cerchiaro M, Barisione E, Giacomini M, Mora S, Baldi F, Balletto E, Berruti M, Briano F, Sepulcri C, Dettori S, Labate L, Mirabella M, Portunato F, Pincino R, Russo C, Tutino S, Pelosi P, Bassetti M; GECOVID study group. Clinical characteristics, management and in-hospital mortality of patients with coronavirus disease 2019 in Genoa, Italy. Clin Microbiol Infect. 2020 Nov;26(11):1537-1544. doi: 10.1016/j.cmi.2020.07.049. Epub 2020 Aug 15. PMID: 32810610; PMCID: PMC7428680. | | |
|  | Vidot *et al.* 2021 | No mental health 🡪 risk/outcome data |
| Vidot DC, Islam JY, Marlene Camacho-Rivera, Harrell MB, Rao DR, Chavez JV, Lucas G Ochoa, Hlaing WM, Weiner M, Messiah SE. The COVID-19 cannabis health study: Results from an epidemiologic assessment of adults who use cannabis for medicinal reasons in the United States. J Addict Dis. 2021 Jan-Mar;39(1):26-36. doi: 10.1080/10550887.2020.1811455. Epub 2020 Sep 15. PMID: 32933383. | | |
|  | Vissink *et al.* 2021 | No mental health 🡪 risk/outcome data |
| Vissink CE, van Hell HH, Galenkamp N, van Rossum IW. The effects of the COVID-19 outbreak and measures in patients with a pre-existing psychiatric diagnosis: A cross-sectional study. J Affect Disord Rep. 2021 Apr;4:100102. doi: 10.1016/j.jadr.2021.100102. Epub 2021 Feb 1. PMID: 33558866; PMCID: PMC7848531. | | |
|  | Volpatto *et al.* 2021 | Review article |
| Volpatto VL, Borgonhi EM, Ornell F, Bavaresco DV, Moura HF, Rabelo-da-Ponte FD, et al. High morbidity and mortality risk due to COVID-19 by smoked drug users. Trends in psychiatry and psychotherapy. 2021. | | |
|  | Vrotsou *et al.* 2021 | Mental health 🡪 course |
| Vrotsou K, Rotaeche R, Mateo-Abad M, Machón M, Vergara I. Variables associated with COVID-19 severity: an observational study of non-paediatric confirmed cases from the general population of the Basque Country, Spain. BMJ Open. 2021 Apr 1;11(4):e049066. doi: 10.1136/bmjopen-2021-049066. PMID: 33795313; PMCID: PMC8024058. | | |
|  | Vukotic *et al.* 2021 | No mental health 🡪 risk/outcome data |
| Vukotic M, Milosevic Z, Bjelica D, Zarubca M. Mental distress during the COVID-19 pandemic of female students adults without a pre-existing mental health condition. African health sciences. 2021;21(4):1544–5. | | |
|  | Wafula *et al.* 2023 | No mental health 🡪 risk and mortality |
| Wafula ST, Ninsiima LL, Mendoza H, Ssempebwa JC, Walter F, Musoke D. Association between recent COVID-19 diagnosis, depression and anxiety symptoms among slum residents in Kampala, Uganda. PLoS One. 2023 May 4;18(5):e0280338. doi: 10.1371/journal.pone.0280338. PMID: 37141298; PMCID: PMC10159354. | | |
|  | Wan *et al.* 2020 a | No mental health 🡪 risk and mortality |
| Wan Y, Wu J, Ni LH, Luo QQ, Yuan C, Fan F, . . . Xie Q (2020). Prognosis analysis of patients with mental  disorders with COVID-19: a single -center retrospective study. *Aging-Us, 12*(12), 11238-11244.  doi:10.18632/aging.103371 | | |
|  | Wang *et al.* 2020 | No mental health 🡪 risk and mortality |
| Wang D, Hu B, Hu C, Zhu F, Liu X, Zhang J, Wang B, Xiang H, Cheng Z, Xiong Y, Zhao Y, Li Y, Wang X, Peng Z. Clinical Characteristics of 138 Hospitalized Patients With 2019 Novel Coronavirus-Infected Pneumonia in Wuhan, China. JAMA. 2020 Mar 17;323(11):1061-1069. doi: 10.1001/jama.2020.1585. | | |
|  | Wang *et al.* 2021 (1) | Mental health 🡪 risk/outcome |
| Wang Y, Yang Y, Ren L, Shao Y, Tao W, Dai XJ. Preexisting Mental Disorders Increase the Risk of COVID-19 Infection and Associated Mortality. Frontiers in public health. 2021;9:684112–684112. | | |
|  | Wang *et al.* 2021 (2) | Mental health 🡪 risk and mortality |
| Wang QQ, Kaelber DC, Xu R, Volkow ND. COVID-19 risk and outcomes in patients with substance use disorders: analyses from electronic health records in the United States. Mol Psychiatry. 2021 Jan;26(1):30-39. doi: 10.1038/s41380-020-00880-7. Epub 2020 Sep 14. Erratum in: Mol Psychiatry. 2020 Sep 30: PMID: 32929211; PMCID: PMC7488216. | | |
|  | Wang *et al.* 2021 (3) | Mental health 🡪 risk |
| Wang Q, Xu R, Volkow ND. Increased risk of COVID-19 infection and mortality in people with mental disorders: analysis from electronic health records in the United States. World Psychiatry. 2021 Feb;20(1):124-130. doi: 10.1002/wps.20806. Epub 2020 Oct 7. PMID: 33026219; PMCID: PMC7675495. | | |
|  | Wang *et al.* 2022 (1 NHS) | Mental health 🡪 COURSE/LONGCOVID PROSPECTIVE |
| Wang S, Quan L, Chavarro JE, Slopen N, Kubzansky LD, Koenen KC, Kang JH, Weisskopf MG, Branch-Elliman W, Roberts AL. Associations of Depression, Anxiety, Worry, Perceived Stress, and Loneliness Prior to Infection With Risk of Post-COVID-19 Conditions. JAMA Psychiatry. 2022 Nov 1;79(11):1081-1091. doi: 10.1001/jamapsychiatry.2022.2640. | | |
|  | Wang *et al.* 2022 (2 NHS) | Mental health 🡪 hospitalization PROSPECTIVE |
| Wang S, Quan L, Ding M, Kang JH, Koenen KC, Kubzansky LD, Branch-Elliman W, Chavarro JE, Roberts AL. Depression, worry, and loneliness are associated with subsequent risk of hospitalization for COVID-19: a prospective study. Psychol Med. 2022 May 19:1-10. doi: 10.1017/S0033291722000691. PMID: 35586906; PMCID: PMC9924056. | | |
|  | Welch *et al.* 2021 | Mental health 🡪 mortality |
| Welch C, for the Geriatric Medicine Research Collaborative; Covid Collaborative. Age and frailty are independently associated with increased COVID-19 mortality and increased care needs in survivors: results of an international multi-centre study. Age Ageing. 2021 May 5;50(3):617-630. doi: 10.1093/ageing/afab026. PMID: 33951161. | | |
|  | Wen *et al.* 2021 | No mental health 🡪 risk/outcome data |
| Wen HF, Barnett ML, Saloner B (2020). Clinical Risk Factors for COVID-19 Among People With Substance Use  Disorders. *Psychiatric Services, 71*(12), 1308-1308. doi:10.1176/appi.ps.202000215 | | |
|  | Wiertz *et al.* 2021 | No mental health 🡪 risk/outcome data |
| Wiertz CMH, Vints WAJ, Maas GJCM, Rasquin SMC, van Horn YY, Dremmen MPM, Hemmen B, Verbunt JA. COVID-19: patient characteristics in the first phase of post-intensive care rehabilitation. Arch Rehabil Res Clin Transl. 2021 Feb 4:100108. doi:10.1016/j.arrct.2021.100108. Epub ahead of print. PMID: 33558860; PMCID: PMC7859717. | | |
|  | Williams *et al.* 2020 | No mental health 🡪 risk/outcome data |
| Williams R, Jenkins DA, Ashcroft DM, Brown B, Campbell S, Carr MJ, Cheraghi-Sohi S, Kapur N, Thomas O, Webb RT, Peek N. Diagnosis of physical and mental health conditions in primary care during the COVID-19 pandemic: a retrospective cohort study. Lancet Public Health. 2020 Oct;5(10):e543-e550. doi:10.1016/S2468-2667(20)30201-2. Epub 2020 Sep 23. PMID: 32979305; PMCID: PMC7511209. | | |
|  | Wisnivesky *et al.* 2022 | No mental health 🡪 risk/outcome data |
| Wisnivesky JP, Govindarajulu U, Bagiella E, Goswami R, Kale M, Campbell KN, et al. Association of Vaccination with the Persistence of Post-COVID Symptoms. Journal of general internal medicine : JGIM. 2022;37(7):1748–53. | | |
|  | Wong *et al.* 2021 | No mental health 🡪 risk/outcome data |
| Wong LP, Alias H. Temporal changes in psychobehavioural responses during the early phase of the COVID-19 pandemic in Malaysia. J Behav Med. 2021 Feb;44(1):18-28. doi: 10.1007/s10865-020-00172-z. Epub 2020 Aug 5. PMID: 32757088; PMCID: PMC7405711. | | |
|  | Wong *et al.* 2023 | No mental health 🡪 risk/outcome data |
| Wong MC, Huang J, Wong YY, Wong GL, Yip TC, Chan RN, Chau SW, Ng SC, Wing YK, Chan FK. Epidemiology, Symptomatology, and Risk Factors for Long COVID Symptoms: Population-Based, Multicenter Study. JMIR Public Health Surveill. 2023 Mar 7;9:e42315. doi: 10.2196/42315. PMID: 36645453; PMCID: PMC9994465. | | |
|  | Woodruff *et al.* 2021 | No mental health 🡪 risk/outcome data |
| Woodruff RC, Campbell AP, Taylor CA, Chai SJ, Kawasaki B, Meek J, et al. Risk Factors for Severe COVID-19 in Children. Pediatrics (Evanston). 2022;149(1):1. | | |
|  | Wright *et al.* 2023 |  |
| Wright A, De Livera A, Lee KH, Higgs C, Nicholson M, Gibbs L, Jorm A. A repeated cross-sectional and longitudinal study of mental health and wellbeing during COVID-19 lockdowns in Victoria, Australia. BMC Public Health. 2022 Dec 27;22(1):2434. doi: 10.1186/s12889-022-14836-9. Erratum in: BMC Public Health.  2023 Jan 13;23(1):97. PMID: 36575409; PMCID: PMC9793381. | | |
|  | Xiang *et al.* 2021 [MEDRXIV] | No mental health 🡪 risk/outcome data [genetic risk] |
| Xiang Y, Qiu J, Zhang R, Chau CKL, Rao S, So HC. (2021). Neuropsychiatric disorders as risk factors and consequences of COVID-19: A Mendelian randomization study. *medRxiv*. | | |
|  | Xie *et al.* 2020 | No mental health 🡪 risk/outcome data |
| Xie Q, Fan F, Fan XP, Wang XJ, Chen MJ, Zhong BL, Chiu HF. COVID-19 patients managed in psychiatric inpatient settings due to first-episode mental disorders in Wuhan, China: clinical characteristics, treatments, outcomes, and our experiences. Transl Psychiatry. 2020 Oct 2;10(1):337. doi: 10.1038/s41398-020-01022-x. PMID: 33009366; PMCID: PMC7531059. | | |
|  | Xu *et al.* 2021A? | No mental health 🡪 risk/outcome data |
| Xu J, Yin Z, Liu Y, Wang S, Duan L, An Y, Fan J, Liao T, Jin Y, Chen J. Clinical characteristics and outcomes of severe or critical COVID-19 patients presenting no respiratory symptoms or fever at onset. Engineering (Beijing). 2020 Oct 29. doi: 10.1016/j.eng.2020.09.009. Epub ahead of print. PMID: 33163252; PMCID: PMC7598919. Y | | |
|  | Xu *et al.* 2023 | Anxiety, bipolar disorder, schizophrenia 🡪 mortality |
| Xu H, Li S, Mehta HB, Hommel EL, Goodwin JS. Excess mortalitys from COVID-19 among Medicare beneficiaries with psychiatric diagnoses: Community versus nursing home. J Am Geriatr Soc. 2023 Jan;71(1):167-177. doi: 10.1111/jgs.18062. Epub 2022 Sep 22. PMID: 36137264; PMCID: PMC9537955. | | |
|  | Yaksi *et al.* 2022 | No mental health 🡪 risk/outcome data |
| Yaksi N, Teker AG, Imre A. Long COVID in Hospitalized COVID-19 Patients: A Retrospective Cohort Study. Iranian journal of public health. 2022;51(1):88–95. | | |
|  | Yang *et al.* 2020 UK | Mental health 🡪 risk and course |
| Yang H, Chen W, Hu Y, Chen Y, Zeng Y, Sun Y, Ying Z, He J, Qu Y, Lu D, Fang F, Valdimarsdottir UA, Song H 2020. Pre-pandemic psychiatric disorders and risk of COVID-19: a UK Biobank cohort analysis. The Lancet Healthy Longevity 1 (2), e69–e79. | | |
|  | Yang *et al.* 2021 | Opinion paper |
| Yang Y, Li W, Zhang Q, Zhang L, Cheung T, Ng CH, Xiang YT. Should people with severe mental illness be prioritized for the COVID-19 vaccination? Int J Biol Sci. 2021 Apr 10;17(6):1443-1445. doi: 10.7150/ijbs.57750. PMID: 33907507; PMCID: PMC8071759. | | |
|  | Yang *et al.* 2022 LA | Mental health 🡪 risk/outcome-anorexia |
| Yang HH, Wu TJ, Yu AC, Wells C, Orshansky G, Lee JT. Predictors of Mortality, Survival, Need for Intubation, and Need for Oxygen Support Among Admitted COVID-19 Patients of the Veterans Affairs Greater Los Angeles Healthcare System. Military medicine. 2022; 188(5-6):1276-1284. doi: 10.1093/milmed/usab550. | | |
|  | Yanover *et al.* 2020 | Mental health 🡪 course |
| Yanover C, Mizrahi B, Kalkstein N, Marcus K, Akiva P, Barer Y, ... & Chodick G (2020). What Factors Increase the Risk of Complications in SARS-CoV-2–Infected Patients? A Cohort Study in a Nationwide Israeli Health Organization. *JMIR public health and surveillance*, *6*(3), e20872. | | |
|  | Yee *et al.* 2021 | No mental health 🡪 risk/outcome data |
| Yee K, Peh HP, Tan YP, Teo I, Tan EUT, Paul J, Rangabashyam M, Ramalingam MB, Chow W, Tan HK. Stressors and coping strategies of migrant workers diagnosed with COVID-19 in Singapore: a qualitative study. BMJ Open. 2021 Mar 19;11(3):e045949. doi: 10.1136/bmjopen-2020-045949. PMID: 33741672; PMCID: PMC7985935. | | |
|  | Yolken 2021 | Opinion paper |
| Yolken R. (2021). COVID-19 and psychiatry: can electronic medical records provide the answers? *Lancet Psychiatry, 8*(2), 89-91. doi:10.1016/s2215-0366(20)30479-x | | |
|  | Yoshida *et al.* 2021 | No mental health 🡪 risk/outcome data |
| Yoshida N, Iwata S, Ogawa M, Izawa KP, Kuroda S, Kohsaka S, et al. Intensive Care Unit Admission for Moderate-to-Severe COVID-19 Patients With Known Cardiovascular Diseases or Their Risk Factors　― Insights From a Nationwide Japanese Cohort Study. Circulation reports. 2021;3(7):375–80. | | |
|  | Zhang *et al.* 2020 | No mental health 🡪 risk/outcome data |
| Zhang J, Wang M, Zhao M, Guo S, Xu Y, Ye J, Ding W, Wang Z, Ye D, Pan W, Liu M, Li D, Luo Z, Liu J, Wan J. The Clinical Characteristics and Prognosis Factors of Mild-Moderate Patients With COVID-19 in a Mobile Cabin Hospital: A Retrospective, Single-Center Study. Front Public Health. 2020 Jun 5;8:264. doi: 10.3389/fpubh.2020.00264. PMID: 32582615; PMCID: PMC7291856. | | |
|  | Zhang *et al.* 2021 | No mental health 🡪 risk/outcome data |
| Zhang H, Wu Y, He Y, Liu X, Liu M, Tang Y, et al. Age-Related Risk Factors and Complications of Patients With COVID-19: A Population-Based Retrospective Study. Frontiers in medicine. 2021;8:757459–757459. | | |
|  | Zhang *et al.* 2021 | Mental health 🡪 no risk/outcome data, anorexia |
| Zhang X, Wang F, Shen Y, Zhang X, Cen Y, Wang B, et al. Symptoms and Health Outcomes Among Survivors of COVID-19 Infection 1 Year After Discharge From Hospitals in Wuhan, China. JAMA network open. 2021;4(9):e2127403–e2127403. | | |
|  | Zhang *et al.* 2023 | No mental health 🡪 risk/outcome data |
| Zhang D, Chung VC, Chan DC, Xu Z, Zhou W, Tam KW, Lee RC, Sit RW, Mercer SW, Wong SY. Determinants of post-COVID-19 symptoms among adults aged 55 or above with chronic conditions in primary care: data from a prospective cohort in Hong Kong. Front Public Health. 2023 May 5;11:1138147. doi:  10.3389/fpubh.2023.1138147. PMID: 37213637; PMCID: PMC10196359 | | |
|  | Zhao *et al.* 2020 | No mental health 🡪 risk/outcome data |
| Zhao A, Li Z, Ke Y, Huo S, Ma Y, Zhang Y, Zhang J, Ren Z. Dietary Diversity among Chinese Residents during the COVID-19 Outbreak and Its Associated Factors. Nutrients. 2020 Jun 6;12(6):1699. doi: 10.3390/nu12061699. PMID: 32517210; PMCID: PMC7352896. Y | | |
|  | Zheng *et al.* 2020 | No mental health 🡪 risk/outcome data |
| Zheng XC, Chen JH, Deng LS, Fang ZX, Chen GQ, Ye D, . . . Hong ZS (2021). Risk factors for the COVID-19  severity and its correlation with viral shedding: A retrospective cohort study. *J Med Virol, 93*(2), 952-961.  doi:10.1002/jmv.26367 | | |
|  | Zhong *et al.* 2021 | No mental health 🡪 risk/outcome data |
| Zhong R, Chen L, Zhang Q, Li B, Qiu Y, Wang W, Tan D, Zou Y. Which Factors, Smoking, Drinking Alcohol, Betel Quid Chewing, or Underlying Diseases, Are More Likely to Influence the Severity of COVID-19? Front Physiol. 2021 Jan 18;11:623498. doi: 10.3389/fphys.2020.623498. PMID: 33536941; PMCID: PMC7849623. | | |
|  | Zhou *et al.* 2020 | No mental health 🡪 risk/outcome data |
| Zhou C, Huang Z, Tan W, Li X, Yin W, Xiao Y, Tao Z, Geng S, Hu Y. Predictive factors of severe coronavirus disease 2019 in previously healthy young adults: a single-center, retrospective study. Respir Res. 2020 Jun 22;21(1):157. doi: 10.1186/s12931-020-01412-1. PMID: 32571410; PMCID: PMC7306646. | | |
|  | Zhu *et al.* 2020 *J Psychiatric Res* | No mental health 🡪 risk/outcome data |
| Zhu Z, Liu Q, Jiang X, Manandhar U, Luo Z, Zheng X, Li Y, Xie J, Zhang B. The psychological status of people affected by the COVID-19 outbreak in China. J Psychiatr Res. 2020 Oct;129:1-7. doi: 10.1016/j.jpsyychires.2020.05.026. Epub 2020 May 28. PMID: 32526513; PMCID: PMC7255091. | | |
|  | Zhu *et al.* 2020 *PLoS ONE* | No mental health 🡪 risk/outcome data |
| Zhu S, Gao Q, Yang L, Yang Y, Xia W, Cai X, Hui Y, Zhu D, Zhang Y, Zhang G, Wu S, Wang Y, Zhou Z, Liu H, Zhang C, Zhang B, Yang J, Feng M, Ni Z, Chen B, Du C, He H, Qu Y, Wei Q, He C, Reinhardt JD. Prevalence and risk factors of disability and anxiety in a retrospective cohort of 432 survivors of Coronavirus Disease-2019 (Covid-19) from China. PLoS One. 2020 Dec 17;15(12):e0243883. doi: 10.1371/journal.pone.0243883. PMID: 33332386; PMCID: PMC7746260. | | |
|  | Zielinska-Turek *et al.* 2021 | No mental health 🡪 risk/outcome data |
| Zielinska-Turek J, Jasinska A, Kolakowska J, Szadurska J, Kosior DA, Dorobek M. (2021). Clinical features of  neurological patients with coronavirus 2019: an observational study of one centre. *Neurol Neurochir Pol,*  *55*(2), 195-201. doi:10.5603/PJNNS.a2021.0011 | | |
|  | Zijlmans *et al.* 2021 | Mental health 🡪 no risk/outcome data |
| Zijlmans J, Teela L, van Ewijk H, Klip H, van der Mheen M, Ruisch H, et al. Mental and Social Health of Children and Adolescents With Pre-existing Mental or Somatic Problems During the COVID-19 Pandemic Lockdown. Frontiers in psychiatry. 2021;12:692853–692853. | | |
|  | Zimering *et al.* 2020 | No mental health 🡪 risk/outcome data |
| Zimering MB, Razzaki T, Tsang T, Shin JJ. Inverse Association between Serotonin 2A Receptor Antagonist Medication Use and Mortality in Severe COVID-19 Infection. Endocrinol Diabetes Metab J. 2020 Sep 15;4(4):1-5. PMID: 33117497; PMCID: PMC7590925. | | |
|  | Zimmermann *et al.* 2021 | No mental health, review paper |
| Zimmermann P, Pittet LF, Curtis N. How Common is Long COVID in Children and Adolescents? The Pediatric infectious disease journal. 2021;40(12):e482–e487. | | |

**Table S3**. Characteristics of included studies and samples by outcome

|  | *Type of study* | Controls / statistical control or matching |
| --- | --- | --- |
| Al-Aly *et al.* 2022 | Retrospective | -/- condition / comorbidities |
| Allen *et al.* 2020 | Retrospective | -/- condition / sociodemographic variables, comorbidities |
| Amin *et al.* 2022 | Retrospective | Healthy controls / no control |
| Azar *al.* 2020 | Retrospective | -/- condition / sociodemographic variables, comorbidities |
| Bailey *et al.* 2021 | Retrospective | -/- condition / sociodemographic variables, comorbidities |
| Baillargeon *et al.* 2021 | Retrospective | -/- condition / propensity matching |
| Barcella *et al.* 2020 | Retrospective | -/- condition / sociodemographic variables, comorbidities |
| Bayrak and Çadirci, 2021 | Prospective | -/- condition / no control |
| Bellan *et al.* 2022 | Prospective | -/- condition / sociodemographic variables, comorbidities |
| Bhopalwala *et al.* 2022 | Retrospective | -/- condition / sociodemographic variables, comorbidities |
| Canal-Rivero *et al.* 2021 | Retrospective | -/- condition / no control |
| Castro *et al.* 2021 | Retrospective | -/- condition / sociodemographic variables, comorbidities (for mood, not for SUD and anxiety) |
| Catalan *et al.* 2022 | Retrospective | -/- condition / sociodemographic variables, comorbidities |
| Cavallaro *et al.* 2021 | Retrospective | -/- condition / sociodemographic variables, comorbidities |
| Chang *et al.* 2021 | Retrospective | -/- condition / sociodemographic variables, comorbidities |
| Chen *et al.* 2022 | Retrospective | -/- condition / sociodemographic variables, comorbidities |
| Chen *et al.* 2021b | Retrospective | -/- condition / sociodemographic variables, comorbidities |
| Clift *et al.* 2020 | Retrospective | -/- condition / sociodemographic variables, comorbidities |
| Clouston *et al.* 2021 | Prospective | -/- condition / sociodemographic variables, comorbidities |
| Cohen *et al.* 2022 | Retrospective | -/- condition / sociodemographic variables, comorbidities |
| Cummins *et al.* 2021 | Retrospective | -/- condition / sociodemographic variables, comorbidities |
| Dai *et al.* 2022 | Prospective | -/- condition / sociodemographic variables, comorbidities |
| Descamps *et al.* 2022 | Retrospective | -/- condition / sociodemographic variables, comorbidities |
| De Miranda *et al.* 2022 | Prospective | -/- condition / No control |
| De Vito *et al.* 2021 | Retrospective | -/- condition / controlled but not further specified |
| Díaz-Simón *et al.* 2021 | Retrospective | -/- condition / No control |
| Diez-Quevedo *et al.* 2021 | Retrospective | -/- condition / controlled but not further specified |
| Durstenfeld *et al.* 2023 | Prospective | -/- condition / No control |
| Egede *et al.* 2021 | Retrospective | -/- condition / sociodemographic variables |
| Fond *et al.* 2021 | Prospective | Null psychiatric diagnosis condition / propensity matching |
| Francis *et al.* 2021 | Prospective | -/- condition / controlled but not further specified |
| Garcia‑Cabrera *et al.* 2021 | Retrospective | -/- condition / no control |
| Gasnier *et al.* 2022 | Retrospective | -/- condition / sociodemographic variables, ICU stay |
| Giannoglou *et al.* 2021 | Retrospective | -/- condition / no control |
| Goldberger *et al.* 2022 | Retrospective | -/- condition / age |
| Haimovich *et al.* 2020 | Retrospective | -/- condition / controlled but not further specified |
| Hashemi‑Shahri *et al.* 2022 | Retrospective | -/- condition / controlled but not further specified |
| Hedberg *et al.* 2023 | Retrospective | -/- comorbidities / sociodemographic variables |
| Hirashima *et al.* 2021 | Retrospective | -/- condition / no control |
| Izurieta *et al.* 2020 | Retrospective | -/- condition / no control |
| Jeon *et al.* 2020 | Retrospective | -/- condition / matching |
| Jones *et al.* 2021 | Retrospective | -/- condition / no control |
| Kundi *et al.* 2020 | Retrospective | -/- condition / no control |
| Lebin *et al.* 2020 | Retrospective | -/- condition / no control |
| Lee *et al.* 2021 a | Prospective | -/- condition / sociodemographic variables, comorbidities |
| Lee *et al.* 2021 b | Retrospective | -/- condition / propensity matching |
| Lega *et al.* 2021 | Retrospective | -/- condition / no control |
| Li *et al.* 2022 | Prospective | -/- condition / sociodemographic variables, comorbidities |
| Maripuu *et al.* 2021 | Retrospective | -/- condition / no control |
| Meinlschmidt *et al.* 2022 | Prospective | -/- condition / sociodemographic variables, comorbidities |
| Merzon *et al.* 2020 | Retrospective | -/- condition / sociodemographic variables, comorbidities |
| Merzon *et al.* 2021 | Retrospective | -/- condition / sociodemographic variables, comorbidities |
| Musheyev *et al.* 2021 | Retrospective | -/- condition / sociodemographic variables, comorbidities |
| Nemani *et al.* 2021 (1) | Retrospective | -/- condition / sociodemographic variables, comorbidities |
| Nemani *et al.* 2021 (2) | Retrospective | -/- condition / sociodemographic variables, comorbidities |
| Nilsson *et al.* 2022 | Retrospective | -/- condition / sociodemographic variables, comorbidities |
| Nishimi *et al.* 2021 | Retrospective | -/- condition / sociodemographic variables, comorbidities |
| Orlando *et al.* 2021 | Retrospective | -/- condition / sociodemographic variables, comorbidities |
| Pavarin *et al.* 2022 | Retrospective | -/- condition / sociodemographic variables, comorbidities |
| Poblador-Plou *et al.* 2021 | Prospective | -/- condition / sociodemographic variables |
| Qeadan *et al.* 2021 | Retrospective | -/- condition / sociodemographic variables, comorbidities |
| Rodríguez-Molinero *et al.* 2020 | Retrospective | -/- condition / no control |
| Salvatore *et al.* 2021 | Retrospective | -/- condition / sociodemographic variables |
| Sisó-Almirall *et al.* 2020 | Retrospective | -/- condition / sociodemographic variables |
| Tang *et al.* 2020 | Retrospective | -/- condition / sociodemographic variables, comorbidities |
| Taquet *et al.* 2021 | Retrospective | -/- condition / sociodemographic variables, comorbidities |
| Teixeira *et al.* 2021 | Retrospective | -/- comorbidities / sociodemographic variables, comorbidities |
| Thompson *et al.* 2022 | Retrospective | -/- condition / sociodemographic variables, comorbidities |
| Tokuda *et al.* 2023 | Retrospective | -/- condition / sociodemographic variables, comorbidities |
| Tzur Bitan *et al.* 2021 | Retrospective | -/- condition / sociodemographic variables, comorbidities |
| Varela-Rodríguez *et al.* 2021 | Retrospective | -/- condition / sociodemographic variables, comorbidities |
| Velásquez García *et al.* 2021 | Retrospective | -/- condition / controlled but not further specified |
| Vrotsou *et al.* 2021 | Retrospective | -/- condition / no control |
| Wang *et al.* 2021 (1) | Retrospective (C-C) | -/- comorbidities / sociodemographic variables |
| Wang *et al.* 2021 (2) | Retrospective (C-C) | -/- condition / sociodemographic variables, comorbidities |
| Wang *et al.* 2022 (3) | Retrospective | -/- condition / matching |
| Wang *et al.* 2022 (1) | Prospective | -/- condition / sociodemographic variables |
| Wang *et al.* 2022 (2) | Prospective | -/- condition / sociodemographic variables |
| Welch *et al.* 2021 | Retrospective | -/- condition / sociodemographic variables, comorbidities |
| Yang *et al.* 2020 | Retrospective | -/- condition / sociodemographic variables, comorbidities |
| Yanover *et al.* 2020 | Retrospective | -/- condition / age |

-/- condition; versus people without the condition under study

-/- comorbidities versus people without the condition under study and no other comorbidity

**Table S4.** Overlap inclusion over meta-analyses and date of assessment.

| ***Study*** | ***In earlier meta-analyses ^1^*** | ***Outcome assessment*** | ***Variant ^2^*** |
| --- | --- | --- | --- |
| Al-Aly *et al.* 2022 | 1 [N], 2 [N], 3 [N], 4 [N] | *Not known* | *Not known* |
| Allen *et al.* 2020 | 1 [N], 2 [Y], 3 [N], 4 [Y] | 01-01-2020 – 26-10-2020 | Wuhan |
| Amin *et al.* 2022 | 1 [N], 2 [N], 3 [N], 4 [N] | O8-2021 – 01-2022 | Omicron |
| Azar *al.* 2020 | 1 [Y], 2 [N], 3 [N], 4 [N] | 01-01-2020 – 08-04-2020 | Wuhan |
| Bailey *et al.* 2021 | 1 [N], 2 [N], 3 [N], 4 [N] | 01-01-2020 – 08-09-2020 | Wuhan |
| Baillargeon *et al.* 2021 | 1 [N], 2 [Y], 3 [N], 4 [Y] | Prior to 14-06-2020 | Wuhan |
| Barcella *et al.* 2020 | 1 [N], 2 [N], 3 [N], 4 [N] | 27-02-2020 – 02-01-2021 | Wuhan |
| Bayrak & Çadirci, 2021 | 1 [N], 2 [N], 3 [N], 4 [N] | 01-08-2020 – 31-10-2020 | Wuhan |
| Bellan *et al.* 2022 | 1 [N], 2 [N], 3 [N], 4 [N] | Prior to 28-06-2021 | Mix |
| Bhopalwala *et al.* 2022 | 1 [N], 2 [N], 3 [N], 4 [N] | 04-2020 – 12-2020 | Wuhan |
| Canal-Rivero *et al.* 2021 | 1 [N], 2 [N], 3 [N], 4 [Y] | 01-03-2020 – 30-11-2020 | Wuhan |
| Castro *et al.* 2021 | 1 [N], 2 [N], 3 [N], 4 [N] | 25-02-2020 – 04-05-2020 | Wuhan |
| Catalan *et al.* 2022 | 1 [N], 2 [N], 3 [N], 4 [N] | 03-2020– 03-2021 | Wuhan |
| Cavallaro *et al.* 2021 | 1 [N], 2 [N], 3 [Y], 4 [N] | Prior to 28-06-2020 | Wuhan |
| Chang *et al.* 2021 | 1 [Y], 2 [N], 3 [N], 4 [N] | 01-01-2020 – 30-09-2020 | Wuhan |
| Chen *et al.* 2021 (1) | 1 [N], 2 [N], 3 [N], 4 [N] | 01-02-2020 – 31-08-2020 | Wuhan |
| Chen *et al.* 2021 (2) | 1 [N], 2 [N], 3 [N], 4 [N] | 01-03-2020 – 28-02-2021 | Mix |
| Clift *et al.* 2020 | 1 [N], 2 [N], 3 [N], 4 [N] | 24-01-2020 – 30-04-2020 | Wuhan |
| Clouston *et al.* 2021 | 1 [N], 2 [N], 3 [N], 4 [N] | 03-07-2020 – 01-09-2020 | Wuhan |
| Cohen *et al.* 2022 | 1 [N], 2 [N], 3 [N], 4 [N] | *Not known* | *Not known* |
| Cummins *et al.* 2021 | 1 [N], 2 [Y], 3 [N], 4 [N] | 01-02-2020 – 30-06-2020 | Wuhan |
| Dai *et al.* 2022 | 1 [N], 2 [N], 3 [N], 4 [N] | *Not known* | *Not known* |
| Descamps *et al.* 2022 | 1 [N], 2 [N], 3 [N], 4 [N] | 03-2020 – 09-2020 | Wuhan |
| De Miranda *et al.* 2022 | 1 [N], 2 [N], 3 [N], 4 [N] | 03-2020 – 11-2021 | Mix |
| De Vito *et al.* 2021 | 1 [N], 2 [N], 3 [N], 4 [N] | 09-03-2020 – 31-04-2020 | Wuhan |
| Díaz-Simón *et al.* 2021 | 1 [N], 2 [N], 3 [N], 4 [N] | 01-03-2020 – 02-07-2020 | Wuhan |
| Diez-Quevedo *et al.* 2021 | 1 [N], 2 [N], 3 [N], 4 [N] | 01-03-2020 – 17-11-2020 | Wuhan |
| Durstenfeld *et al.* 2023 | 1 [N], 2 [N], 3 [N], 4 [N] | 26-03-2020 – 04-04-2022 | Mix |
| Egede *et al.* 2021 | 1 [N], 2 [Y], 3 [N], 4 [N] | 01-03-2020 – 10-07-2020 | Wuhan |
| Fond *et al.* 2021 | 1 [N], 2 [Y], 3 [Y], 4 [Y] | 01-02-2020 – 09-06-2020 | Wuhan |
| Francis *et al.* 2021 | 1 [N], 2 [N], 3 [N], 4 [N] | 17-03-2020 – 17-05-2020 | Wuhan |
| Garcia‑Cabrera *et al.* 2021 | 1 [N], 2 [N], 3 [N], 4 [N] | 01-03-2020 – 01-05-2020 | Wuhan |
| Gasnier *et al.* 2022 | 1 [N], 2 [N], 3 [N], 4 [N] | 15-07-2020 – 18-09-2020 | Wuhan |
| Giannoglou *et al.* 2021 | 1 [N], 2 [N], 3 [Y], 4 [N] | 21-02-2020 – 30-06-2020 | Wuhan |
| Goldberger *et al.* 2022 | 1 [N], 2 [N], 3 [N], 4 [N] | 01-03-2020 – 31-03-2020 | Wuhan |
| Haimovich *et al.* 2020 | 1 [Y], 2 [N], 3 [N], 4 [N] | 01-03-2020 – 08-04-2020 | Wuhan |
| Hashemi‑Shahri *et al.* 2022 | 1 [N], 2 [N], 3 [N], 4 [N] | 29-02-2020 – 31-04-2020 | Wuhan |
| Hedberg *et al.* 2022 | 1 [N], 2 [N], 3 [N], 4 [N] | 01-03-2020 – 31-07-2021 | Mix |
| Hesni *et al.* 2022 | 1 [N], 2 [N], 3 [N], 4 [N] | *Not known* | *Not known* |
| Hirashima *et al.* 2021 | 1 [N], 2 [N], 3 [Y], 4 [N] | 20-02-2020 – 30-04-2020 | Wuhan |
| Izurieta *et al.* 2020 | 1 [Y], 2 [N], 3 [N], 4 [Y] | 01-04-2020 – 08-05-2020 | Wuhan |
| Jeon *et al.* 2020 | 1 [Y], 2 [Y], 3 [Y], 4 [N] | 01-12-2019 – 15-05-2020 | Wuhan |
| Jones *et al.* 2021 | 1 [N], 2 [N], 3 [N], 4 [N] | 07-08-2020 – 20-01-2021 | Wuhan |
| Kundi *et al.* 2020 | 1 [Y], 2 [N], 3 [N], 4 [N] | 11-03-2020 – 22-06-2020 | Wuhan |
| Lebin *et al.* 2020 | 1 [N], 2 [N], 3 [N], 4 [N] | 01-05-2020 – 31-07-2020 | Wuhan |
| Lee *et al.* 2021 (1) | 1 [N], 2 [Y], 3 [Y], 4 [Y] | 01-01-2020 – 10-04-2020 | Wuhan |
| Lee *et al.* 2021 (2) | 1 [N], 2 [Y], 3 [Y], 4 [N] | 01-01-2020 – 15-05-2020 | Wuhan |
| Lega *et al.* 2021 | 1 [N], 2 [N], 3 [N], 4 [N] | 01-01-2020 – 14-12-2020 | Wuhan |
| Li *et al.* 2022 | 1 [N], 2 [N], 3 [N], 4 [N] | 06-01-2020 – 09-03-2020 | Wuhan |
| Maripuu *et al.* 2021 | 1 [N], 2 [N], 3 [N], 4 [N] | 11-03-2020 – 15-06-2020 | Wuhan |
| Merzon *et al.* 2020 | 1 [N], 2 [N], 3 [N], 4 [N] | 01-02-2020 – 30-06-2020 | Wuhan |
| Merzon *et al.* 2021 | 1 [N], 2 [N], 3 [N], 4 [N] | 01-02-2020 – 30-04-2020 | Wuhan |
| Meinschmidt *et al.* 2022 | 1 [N], 2 [N], 3 [N], 4 [N] | *Not known* | *Not known* |
| Musheyev *et al.* 2021 | 1 [N], 2 [N], 3 [N], 4 [N] | 27-03-2020 – 11-08-2020 | Wuhan |
| Nemani *et al.* 2021 (1) | 1 [Y], 2 [Y], 3 [Y], 4 [Y] | 03-03-2020 – 17-02-2021 | Mix |
| Nemani *et al.* 2021 (2) | 1 [N], 2 [N], 3 [N], 4 [N] | 03-03-2020 – 15-07-2020 | Wuhan |
| Nilsson *et al.* 2022 | 1 [N], 2 [N], 3 [N], 4 [N] | 27-02-2020 – 15-10-2021 | Mix |
| Nishimi *et al.* 2022 | 1 [N], 2 [N], 3 [N], 4 [N] | 20-02-2020 – 16-11-2021 | Mix |
| Orlando *et al.* 2021 | 1 [Y], 2 [N], 3 [N], 4 [N] | Prior to 10-06-2020 | Wuhan |
| Pavarin *et al.* 2022 | 1 [N], 2 [N], 3 [N], 4 [N] | 01-01-2020 – 30-12-2020 | Wuhan |
| Poblador-Plou *et al.* 2021 | 1 [Y], 2 [Y], 3 [N], 4 [Y] | 04-03-2020 – 17-04-2020 | Wuhan |
| Qeadan *et al.* 2021 | 1 [N], 2 [N], 3 [N], 4 [N] | 01-01-2020 – 30-06-2020 | Wuhan |
| Rodríguez-M. *et al.* 2020 | 1 [Y], 2 [N], 3 [N], 4 [N] | 12-03-2020 – 02-05-2020 | Wuhan |
| Salvatore *et al.* 2021 | 1 [Y], 2 [N], 3 [N], 4 [N] | 10-03-2020 – 02-10-2020 | Wuhan |
| Sisó-Almirall *et al.* 2020 | 1 [N], 2 [N], 3 [N], 4 [Y] | 29-02-2020 – 04-04-2020 | Wuhan |
| Tang *et al.* 2020 | 1 [Y], 2 [N], 3 [N], 4 [N] | 01-02-2020 – 12-06-2020 | Wuhan |
| Taquet *et al.* 2021 | 1 [Y], 2 [Y], 3 [N], 4 [N] | 01-03-2020 – 01-06-2020 | Wuhan |
| Teixeira *et al.* 2021 | 1 [N], 2 [N], 3 [N], 4 [N] | 01-01-2020 – 29-12-2020 | Wuhan |
| Thompson *et al.* 2022 | 1 [N], 2 [N], 3 [N], 4 [N] | Prior to spring 2021 | Mix |
| Tokuda *et al.* 2023 | 1 [N], 2 [N], 3 [N], 4 [N] | 01-01-2020 – 30-11-2021 | Mix |
| Tzur Bitan *et al.* 2021 | 1 [N], 2 [Y], 3 [N], 4 [Y] | Prior to October 2020 | Wuhan |
| Varela-Rodríguez *et al.* 2021 | 1 [N], 2 [N], 3 [N], 4 [N] | 25-02-2020 – 04-09-2020 | Wuhan |
| Velásquez García *et al.* 2021 | 1 [N], 2 [N], 3 [N], 4 [N] | 26-01-2020 – 15-01-2020 | Mix |
| Vrotsou *et al.* 2021 | 1 [N], 2 [N], 3 [N], 4 [N] | 28-02-2020 – 31-05-2020 | Wuhan |
| Wang *et al.* 2021 (1) | 1 [Y], 2 [N], 3 [Y], 4 [N] | 01-01-2020 – 03-02-2020 | Wuhan |
| Wang *et al.* 2022 (2) | 1 [Y], 2 [N], 3 [Y], 4 [N] | Prior to 15-06-2020 | Wuhan |
| Wang *et al.* 2021 (3) | 1 [N], 2 [N], 3 [N], 4 [N] | Prior to 24-02-2021 | Mix |
| Wang *et al.* 2022 (1 and 3) | 1 [N], 2 [N], 3 [N], 4 [N] | 04-2020 – 05-2020 | Wuhan |
| Welch *et al.* 2021 | 1 [N], 2 [N], 3 [N], 4 [Y] | Prior to 2021 | Wuhan |
| Yang *et al.* 2020 | 1 [Y], 2 [Y], 3 [Y], 4 [Y] | 01-01-2020 – 28-06-2020 | Wuhan |
| Yanover *et al.* 2020 | 1 [Y], 2 [Y], 3 [N], 4 [Y] | Prior to 22-04-2020 | Wuhan |

^1^ 1. Ceban *et al.,* 2021), 2. Fond *et al.,* 2021, 3. Toubasi *et al.,* 2021, Vai *et al.,* 2021.

^2^ Wuhan [December 2020], Alfa [January – June 2021], Delta [June 2021], Omicron [October 2021]

**Table S5**. Characteristics of included studies and samples by outcome

|  | Predictor [Method] | Outcome |
| --- | --- | --- |
| Al-Aly *et al.* 2022 | Time frame not known, any mental health conditions [ICD 10] | Infection risk, mortality, long covid  Covid assessment: not known |
| Allen *et al.* 2020 | Lifetime SUD [ICD 10] | Infection risk, hospitalization, ICU admission, mortality  Covid assessment: positive PCR |
| Amin *et al.* 2022 | Time frame not known, schizophrenia, age [ICD 10] | Infection risk  Covid assessment: positive PCR |
| Azar *et al.* 2020 | Timeframe not known, mood disorder [ICD 10] | Infection risk, hospitalization, ICU admission, mortality  Covid assessment: any kind |
| Bailey *et al.* 2021 | Lifetime, any mental health conditions [ICD-10] | Infection risk Covid assessment: diagnosis |
| Baillargeon *et al.* 2021 | Lifetime SUD [ICD 10] | Hospitalization, ventilator use, mortality  Covid assessment: |
| Barcella *et al.* 2020 | Lifetime schizophrenia spectrum, bipolar depression, unipolar depression, other disorders [ICD 8 and 10] | Severe COVID19 (yes *vs* no), mortality  Covid assessment: diagnosis |
| Bayrak & Çadirci, 2021 | Current / lifetime unknown, Depression – method unknown | Mortality  Covid assessment: positive PCR |
| Bellan *et al.* 2022 ***^2^*** | Current anxiety and depressive symptoms [MINI] | Longcovid  Covid assessment: not known |
| Bhopalwala *et al.* 2022 | Time frame not known, opiate or alcohol dependence [ICD-10] | Duration of hospitalization, readmission, mortality  Covid assessment: diagnosis |
| Canal-Rivero *et al.* 2021 | Current severe mental disorders (psychotic spectrum, affective spectrum, personality disorders, other disorders)[ICD 10] | Infection risk, hospitalization, ICU admission, mortality  Covid assessment: not known |
| Castro *et al.* 2021 | Lifetime mood disorders incl. bipolar disorder, SUD, anxiety disorder [ICD 10] | Hospital discharge to nursing facility/with care *vs* home, mortality. Effect size on hospital discharge was recalculated because of a prevalence > .10  Covid assessment: positive test |
| Catalan *et al.* 2021 | Lifetime affective disorders, SUD, anxiety disorders, psychosis, personality disorders and eating disorders [ICD 8-10, DSM IV] | Mortality  Covid assessment: not known |
| Cavallaro *et al.* 2021 | Lifetime serious mental illness [incl. a.o., depression, anxiety, and schizophrenia], diagnosed by the UK national health services – exact method is unknown | ICU admissions, Mortality  Covid assessment: not known |
| Chang *et al.* 2021 | Lifetime depression, schizophrenia, opioid use disorder [ICD 10-CM] | Hospitalization  Covid assessment: diagnosis |
| Chen *et al.* 2022 (1) | Current / lifetime unknown, severe mental illness | Mortality  Covid assessment: not known |
| Chen *et al.* 2021 (2) | Current / lifetime unknown, severe mental illness, dementia, lockdown exposed vs lockdown unexposed | Mortality  Covid assessment: positive PCR |
| Clift *et al.* 2021 | Lifetime severe mental illness, amongst which bipolar disorder, psychosis, schizophrenia or schizoaffective disorder, severe depression – exact method is unknown | Hospitalization (for mortality, the UK biobank data is preferred)  Covid assessment: diagnosis |
| Clouston *et al.* 2021 | Current/lifetime unknown depression, comorbidities | Mortality, final discharge  Covid assessment: not known |
| Cohen *et al.* 2022 | ADHD (medication treatment yes vs no) vs non-ADHD | Infection risk  Covid assessment: diagnosis |
| Cummins *et al.* 2021 | Lifetime depression and sever mental illness – exact diseases and method unknown | Hospitalization, ICU admissions, mortality  Covid assessment: diagnosis |
| Dai *et al.* 2022 | Lifetime mental disorders [ICD 10] | Infection risk  Covid assessment: diagnosis |
| Descamps *et al.* 2022 | Lifetime psychotic disorders, mood disorders, anxiety disorders, SUD [ICD 10] | Course, ICU admission, mortality Covid assessment: diagnosis |
| De Miranda *et al.* 2022 | Depression diagnosis, timw frame not known | Longcovid  Covid assessment: diagnosis |
| De Vito *et al.* 2021 | Diagnosed mental illness, exact method is unknown | Infection risk, symptomatic, mortality  Covid assessment: positive PCR |
| Díaz-Simón *et al.* 2021 | Alcohol use disorder, timeframe and method unknown | incidence of respiratory  failure, ICU admission, mortality, length of hospital stay  Covid assessment: positive PCR |
| Diez-Quevedo *et al.* 2021 | Current alcohol use disorder, mood disorder, ‘stress, anxiety, adjustment disorder’ [ICD-10] | Mortality  Covid assessment: diagnosis |
| Durstenfeld *et al.* 2023 | Precovid depression (PHQ-9) and anxiety (GAD-7) symptoms | Longcovid  Covid assessment: not known |
| Egede *et al.* 2021 | Current bipolar, psychotic-, internalizing-, externalizing- disorders [ICD 9 and 10] | Infection risk, hospitalization, mortality  Covid assessment: positive PCR |
| Fond *et al.* 2021 | Time frame not known, schizophrenia [ICD 10 and CCAM] | ICU admission, mortality  Covid assessment: diagnosis |
| Francis *et al.* 2021 | Current / lifetime unknown, diagnosed mental health status - method unknown | Deterioration  Covid assessment: mix |
| Garcia‑Cabrera *et al.* 2021 | Current mental and functional health status, comorbidities | Emergency department referrals, mortality, hospitalization  Covid assessment: mix |
| Gasnier *et al.* 2022 | Current psychiatric disorders [DSM 5] | Longcovid  Covid assessment: not known |
| Giannoglou *et al.* 2021 | Current / lifetime unknown, diagnosed mental health disease – exact method is unknown | Mortality  Covid assessment: not known |
| Goldberger *et al.* 2022 | Current [ICD 10], psychiatric disorders (schizophrenia, affective disorders, bipolar disorder | Infection risk, hospitalization, mortality  Covid assessment: positive PCR |
| Haimovich *et al.* 2020 | Lifetime psychosis, depression [ICD 9 and 10] | Infection risk  Covid assessment: diagnosis |
| Hashemi‑Shahri *et al.* 2022 | Current substance abuse | Severity  Covid assessment: not known |
| Hedberg *et al.* 2023 ***^2^*** | Current / lifetime unknown, diagnosed mental health disease – exact method is unknown | Longcovid  Covid assessment: diagnosis |
| Hirashima *et al.* 2021 | Current / lifetime unknown, panic and OCD – exact method is unknown | Severity  Covid assessment: positive PCR |
| Izurieta *et al.* 2020 | Lifetime depression [ICD 10 CM] | Hospitalization, mortality  Covid assessment: diagnosis |
| Jeon *et al.* 2020 | Current mood disorders and schizophrenia/schizotypal/delusional disorders [ICD 10] | Severity, ICU admission, mortality  Covid assessment: positive PCR |
| Jones *et al.* 2021 ^2^ | Mental disorders [mix assessment] | Longcovid  Covid assessment: self-diagnosis |
| Kundi *et al.* 2020 | Depression, SUD [ICD 10 CM] | Mortality  Covid assessment: positive PCR |
| Lebin *et al.* 2020 | Lifetime AUD – exact method is unknown | Infection risk  Covid assessment: positive PCR |
| Lee *et al.* 2021 a | Past year anxiety and stress related disorders, mood disorders, SUD, personality disorders, eating disorders [ICD 10] | Infection risk  Covid assessment: diagnosis |
| Lee *et al.* 2021 b | 6-month mental disorder of any type – [ICD 10] | Mortality  Covid assessment: not known |
| Lega *et al.* 2021 | Lifetime severe psychiatric disorder (psychotic- and bipolar disorder) and common mental disorder (depression and anxiety) [ICD 10] | Severity, ICU admission  Covid assessment: positive PCR |
| Li *et al.* 2022 | Current depression and anxiety. Validated cut-off score on the HADS | Course  Covid assessment: diagnosis |
| Maripuu *et al.* 2021 | Lifetime severe mental disorder; psychotic disorders, bipolar disorders [ICD 10] | Mortality  Covid assessment: diagnosis |
| Merzon *et al.* 2020 | Lifetime autism spectrum disorder, depression and anxiety, schizophrenia, ADHD [ICD 9 and 10] | Infection risk  Covid assessment: positive PCR |
| Merzon *et al.* 2021 | Lifetime autism spectrum disorder, depression and anxiety, schizophrenia, ADHD [ICD 9 and 10] | Hospitalization, symptomatic  Covid assessment: positive PCR |
| Musheyev *et al.* 2021 | Any psychiatric disorder. Time frame not known, in hospital rehabilitation | pre-COVID-19 admission, discharge assistive equipment, length of stay,  discharge medical follow-up refferals  Covid assessment: positive PCR |
| Nemani *et al.* 2021 (1) | Current schizophrenia, schizoaffective disorder, or bipolar disorder [ICD 10] | Mortalitiy  Covid assessment: |
| Nemani *et al.* 2021 (2) | Current anxiety disorder, mood disorder, schizophrenia spectrum disorder [ICD 10R CM] | Infection risk, 45-day mortality or hospice  Covid assessment: positive PCR |
| Nilsson *et al.* 2022 | Current substance abuse, severe mental illness [ICD 8 and 10] | Hospitalization, mortality, intensive care admission  Covid assessment: positive PCR |
| Nishimi *et al.* 2021 | Current depressive disorder, posttraumatic stress disorder, anxiety disorders, alcohol use disorder, substance use disorders, bipolar disorders, psychotic disorder, attention-deficit/hyperactivity disorder, eating disorder [ICD 9 and 10] | Breakthrough Infection risk  Covid assessment: positive PCR |
| Orlando *et al.* 2021 | Lifetime psychosis, depression, anxiety (ICD-9) | Test positivity, mortality  Covid assessment: positive PCR |
| Pavarin *et al.* 2022 | Lifetime SUD or AUD [ICD 10 or 9] | Hospitalization rate, mortality  Covid assessment: not known |
| Poblador-Plou *et al.* 2021 | Current adjustment disorder, anxiety disorders, developmental disorders, mood disorders, personality disorders, schizophrenia/psychotic disorders, substance-related disorders, [ICD 9 CM] | Mortality  Covid assessment: not known |
| Qeadan *et al.* 2021 | Current OUD [ICD 9 and 10] | Hospitalization, severity, mortality  Covid assessment: not known |
| Rodríguez-Molinero *et al.* 2020 | Lifetime AUD, schizophrenia, depression, other psychiatric disorders | Course (severe vs mild) and mortality  Covid assessment: positive PCR |
| Salvatore *et al.* 2021 | Lifetime anxiety, bipolar disorder, depression, SUD, schizophrenia/psychosis [ICD 9 and 10] | Hospitalization, ICU admission, mortality  Covid assessment: positive PCR |
| Sisó-Almirall *et al.* 2020 | Depression, method of assessment and time frame not known | Mortality/ICU admission, hospitalization  Covid assessment: positive PCR |
| Tang *et al.* 2020 | Depression, time frame not known [ICD 10R CM] | Infection risk, hospitalization, mortality  Covid assessment: positive PCR |
| Taquet *et al.* 2021 | Current any psychiatric illness [ICD 10] | Infection risk  Covid assessment: diagnosis |
| Teixeira *et al.* 2021 | Time frame not known, schizophrenia, mood disorders, anxiety disorders [ICD 9 and 10] | Infection risk and mortality  Covid assessment: positive PCR |
| Thompson *et al.* 2022 | Mental health disorders. Time frame unknown | Long COVID  Covid assessment: self-diagnosis |
| Tokuda *et al.* 2023 | Lifetime schizophrenia and psychotic disorders, bipolar disorder [ICD 10] | In hospital mortality  Covid assessment: positive PCR |
| Tzur Bitan *et al.* 2021 | Lifetime schizophrenia [ICD 9 and 10] | Infection risk, hospitalization, mortality  Covid assessment: not known |
| Varela-Rodríguez *et al.* 2021 | Lifetime AUD [ICD 10 and DSM 5] | Infection risk, ICU admission, respirator need, complications  Covid assessment: diagnosis |
| Velásquez García *et al.* 2021 | Current schizophrenia and psychotic disorders, mood and anxiety disorders, dementia, SUD [ICD 9 and 10] | Hospitalization  Covid assessment: positive PCR |
| Vrotsou *et al.* 2021 | Lifetime psychotic disorders, other mental disorders [ICD 9] | Hospitalization (incl. ICU), mortality  Covid assessment: positive PCR+ |
| Wang *et al.* 2021 (1) | Time frame not known, psychotic disorder, substance use disorder, bipolar disorder, anxiety, and depression [ICD 10] | Risk of infection and mortality  Covid assessment: diagnosis |
| Wang *et al.* 2021 (2) | Current, any SUD, SNOMED concept codes | Infection risk, hospitalization (incl. ICU), mortality  Covid assessment: diagnosis |
| Wang *et al.* 2021 (3) | Lifetime (of whom 63% recent) ADHD, bipolar disorder, unipolar depression, schizophrenia SNOMED concept codes | Hospitalization, mortality  Covid assessment: positive PCR |
| Wang *et al.* 2022 (1) | Lifetime depression, through multiple indicators, a.o., CESD-10 depression | Longcovid  Covid assessment: self-reported |
| Wang *et al.* 2022 (2) | Lifetime depression, through multiple indicators, a.o., CESD-10 depression. Current diagnosis may concur with the onset of COVID. | Hospitalization  Covid assessment: self-reported |
| Welch *et al.* 2021 | Lifetime, mental health, diagnosed by a clinician [ICD and DSM] | ICU admission, care after discharge, mortality  Covid assessment: positive PCR |
| Yang *et al.* 2020 | Current/recent depression, anxiety, stress-related disorders, SUD, psychotic disorder [ICD 9 and ICD 10) | Infection risk, inpatient status, mortality  Covid assessment: mix |
| Yanover *et al.* 2020 | Depression, no information on he time frame nor on the exact method. | Complicated disease  Covid assessment: positive PCR |

*Abbreviations.* CCAM, Classification Commune des Actes M.dicaux (CCAM); DSM, Diagnostic and Statistical Manual of Mental Disorders; ICD, International Classification of Disease, ICU, Intensive Care Unit

| **BOX 2.** Article selection to avoid overlapping data sets |
| --- |
| *Overlap prior to inclusion of articles*  Below we describe our decisions for countries for which potential overlap was an issue.  *UK Biobank*  Seven articles (Atkins *et al.,* 2020, Batty *et al.,* 2021, Batty *et al.,* 2020; Yang *et al.,* 2020, van der Meer *et al.,* 2020, Kolin *et al.,* 2021; Kirov *et al.,* 2021 ; Wang *et al.,* 2021[3]) reported on data derived from the UK Biobank. Based on the above specified criteria we only included Yang *et al.* 2020. Clift *et al.* 2021 do not report on UK Biobank data but they do report on COVID-19 related mortality based on UK nationwide data. Given that it is highly likely that this data set will have overlap with the UK Biobank, we also excluded the reporting on mortality in this study from further analysis. We did use Clift *et al.* for analysis on hospitalization, since Yang *et al.* 2020 do not report on this outcome. For the overall analysis on COVID-19 course we excluded Clift *et al.* 2021 because of a relative lack of specificity of the predictor variable ‘severe mental illness’. We did not include Wang *et al.,* 2021[3] for mortality outcomes because of sample size.  *Danish nation wide*  Barcella *et al.* (2021) report on SARS-CoV-2 infection rate and COVID-19 related mortality based on Danish nationwide data. For this reason, the Danish infection risk data and the mortality data reported by Reilev *et al.* (2021) and Brieghel *et al.* (2021) respectively, were not included in the meta-analysis on mortality. The data reported by Nilsson *et al.* (2021) were used in analyses of SUD and the mix category mental disorders on hospitalization and ICU admission and mortality.  *USA nation wide*  Four articles reported on nationwide data from the USA (Murk *et al.* 2021, Taquet *et al.* 2021; Wang *et al.* 2021 a; Wang *et al.* 2021 b). Based on the above, we decided to include Taquet *et al.* 2021 in the meta-analysis on risk for non-SUD patients above Wang *et al.* 2021 b. Wang *et al.* 2021 a was included in the meta-analysis on infection risk and COVID-19 course of SUD patients. Wang *et al.* 2021 b, was included in analyses on course and mortality analyses for patients with non-SUD mental disorders. Murk *et al.* 2021 was excluded because of the smallest sample size.  *South-Korean nation-wide data*  We detected 7 published records that used (parts) of nationwide data from gathered in South Korea *(*Lee *et al.* 2021 a Lee *et al.* 2021 b; Lee *et al.* 2021 c; Park and Rhim, 2021; Kim *et al.* 2021; Jeon *et al.* 2020 *risk* An *et al.* 2020). Kim *et al.* 2021 and An *et al.* 2020 were excluded for reasons specified above. Lee *et al.* 2021 b was excluded in the met-analysis on COVID-19 course and mortality in the general population. For these outcomes we favored Jeon *et al.* 2020 as their predictor variables are more specific. Lee *et al.* 2021 a was included in the meta-analysis on infection rate. Lee *et al.* 2021 c was included in the meta-analysis on mortality in older adults.  References to the articles mentioned here can be found in **Table S2**.  *Overlap after inclusion of articles*  After inclusion we examined the per predictor variable and outcome and tried to optimize the data in order to both avoid overlap and loss of relevant data (see the supplemental excel file). |

**Table S6.** Quality assessment of included prospective and cross-sectional studies (**T** is total score)

| Al-Aly *et al.* 2022 | 1**⊕**; 2**∅**; 3**∅**; 4a**⊕**; 4b**∅**; 5**⊕**; 6**⊕**; 7**⊕**; 8**⊗**; 9**∅**; 10**∅**; 11**⊕**; 12**∅**; 13**⊕**; 14**⊕**: **T = 4** |
| --- | --- |
| Allen *et al.* 2020 | 1**⊕**; 2**⊕**; 3**∅**; 4a**⊕**; 4b**⊕**; 5**⊗**; 6**⊕**; 7**⊕**; 8**⊕**; 9**⊕**; 10**⊗**; 11**⊕**; 12**∅**; 13**⊕**; 14**⊕**: **T = 6** |
| Amin *et al.* 2022 | 1**⊕**; 2**∅**; 3**∅**; 4a**⊕**; 4b**∅**; 5**⊕**; 6**⊕**; 7**⊕**; 8**⊗**; 9**∅**; 10**∅**; 11**⊕**; 12**∅**; 13**⊕**; 14**⊕**: **T = 3** |
| Azar *al.* 2020 | 1**⊕**; 2**⊕**; 3**∅**; 4a**∅**; 4b**∅**; 5**⊗**; 6**⊕**; 7**⊕**; 8**⊗**; 9**⊗**; 10**∅**; 11**⊕**; 12**∅**; 13**⊕**; 14**⊕**: **T = 2** |
| Bailey *et al.* 2021 | 1**⊕**; 2**⊕**; 3**∅**; 4a**⊕**; 4b**⊕**; 5**⊗**; 6**⊕**; 7**⊕**; 8**⊗**; 9**⊗**; 10**⊗**; 11**⊕**; 12**∅**; 13**⊕**; 14**⊗**: **T = 3** |
| Baillargeon *et al.* 2021 | 1**⊕**; 2**⊕**; 3**∅**; 4a**⊕**; 4b**⊕**; 5**⊗**; 6**⊕**; 7**⊕**; 8**⊕**; 9**⊗**; 10**⊗**; 11**⊕**; 12**∅**; 13**⊕**; 14**⊕**: **T = 5** |
| Barcella *et al.* 2020 | 1**⊕**; 2**⊕**; 3**⊕**; 4a**⊕**; 4b**⊕**; 5**⊕**; 6**⊕**; 7**⊕**; 8**⊕**; 9**⊕**; 10**⊗**; 11**⊕**; 12**∅**; 13**⊕**; 14**⊕**: **T = 10** |
| Bayrak & Çadirci, 2021 | 1**⊕**; 2**⊕**; 3**∅**; 4a**⊕**; 4b**⊕**; 5**⊗**; 6**⊕**; 7**⊕**; 8**⊗**; 9**⊗**; 10**⊗**; 11**⊕**; 12**∅**; 13**⊕**; 14**⊗**: **T = 3** |
| Bellan *et al.* 2022 ***^2^*** | 1**⊕**; 2**∅**; 3**∅**; 4a**⊕**; 4b**∅**; 5**⊕**; 6**⊕**; 7**⊕**; 8**⊗**; 9**∅**; 10**∅**; 11**⊕**; 12**∅**; 13**⊕**; 14**⊕**: **T = 7** |
| Bhopalwala *et al.* 2022 | 1**⊕**; 2**∅**; 3**∅**; 4a**⊕**; 4b**∅**; 5**⊕**; 6**⊕**; 7**⊕**; 8**⊗**; 9**∅**; 10**∅**; 11**⊕**; 12**∅**; 13**⊕**; 14**⊕**: **T = 5** |
| Canal-Rivero *et al.* 2021 | 1**⊕**; 2**⊕**; 3**⊕**; 4a**∅**; 4b**∅**; 5**⊗**; 6**⊕**; 7**⊕**; 8**⊗**; 9**⊗**; 10**∅**; 11**⊕**; 12**∅**; 13**⊕**; 14**⊗**: **T = 1** |
| Castro *et al.* 2021 | 1**⊕**; 2**⊕**; 3**⊕**; 4a**⊕**; 4b**⊕**; 5**⊗**; 6**⊕**; 7**⊕**; 8**⊗**; 9**⊗**; 10**⊗**; 11**⊕**; 12**∅**; 13**⊕**; 14**∅**: **T = 3** |
| Catalan *et al.* 2021 | 1**⊕**; 2**∅**; 3**∅**; 4a**⊕**; 4b**∅**; 5**⊕**; 6**⊕**; 7**⊕**; 8**⊗**; 9**∅**; 10**∅**; 11**⊕**; 12**∅**; 13**⊕**; 14**⊕**: **T = 5** |
| Cavallaro *et al.* 2021 | 1**⊕**; 2**⊕**; 3**∅**; 4a**∅**; 4b**∅**; 5**⊗**; 6**⊕**; 7**⊕**; 8**⊗**; 9**⊗**; 10**⊗**; 11**⊕**; 12**∅**; 13**⊕**; 14**⊗**: **T = 1** |
| Chang *et al.* 2021 | 1**⊕**; 2**⊕**; 3**⊕**; 4a**∅**; 4b**∅**; 5**⊗**; 6**⊕**; 7**⊕**; 8**⊗**; 9**⊗**; 10**∅**; 11**⊕**; 12**∅**; 13**⊕**; 14**⊕**: **T = 3** |
| Chen *et al.* 2021 (1) | 1**⊕**; 2**∅**; 3**∅**; 4a**⊕**; 4b**∅**; 5**⊕**; 6**⊕**; 7**⊕**; 8**⊗**; 9**∅**; 10**∅**; 11**⊕**; 12**∅**; 13**⊕**; 14**⊕**: **T = 5** |
| Chen *et al.* 2021 (2) | 1**⊕**; 2**∅**; 3**∅**; 4a**⊕**; 4b**∅**; 5**⊕**; 6**⊕**; 7**⊕**; 8**⊗**; 9**∅**; 10**∅**; 11**⊕**; 12**∅**; 13**⊕**; 14**⊕**: **T = 5** |
| Clift *et al.* 2020 | 1**⊕**; 2**⊕**; 3**⊕**; 4a**∅**; 4b**∅**; 5**⊗**; 6**⊕**; 7**⊕**; 8**⊗**; 9**⊗**; 10**∅**; 11**⊕**; 12**∅**; 13**⊕**; 14**⊕**: **T = 3** |
| Clouston *et al.* 2021 | 1**⊕**; 2**⊗**; 3**∅**; 4a**∅**; 4b**⊗**; 5**⊗**; 6**⊕**; 7**⊕**; 8**⊕**; 9**⊕**; 10**⊗**; 11**⊕**; 12**∅**; 13**⊕**; 14**⊗**: **T = 2** |
| Cohen *et al.* 2022 | 1**⊕**; 2**⊗**; 3**∅**; 4a**∅**; 4b**⊗**; 5**⊗**; 6**⊕**; 7**⊕**; 8**⊕**; 9**⊕**; 10**⊗**; 11**⊕**; 12**∅**; 13**⊕**; 14**⊗**: **T = 0** |
| Cummins *et al.* 2021 | 1**⊕**; 2**⊕**; 3**∅**; 4a**∅**; 4b**∅**; 5**⊗**; 6**⊕**; 7**⊕**; 8**⊕**; 9**⊗**; 10**∅**; 11**⊕**; 12**∅**; 13**⊕**; 14**⊕**: **T = 4** |
| Dai *et al.* 2022 | 1**⊕**; 2**⊗**; 3**∅**; 4a**∅**; 4b**⊗**; 5**⊗**; 6**⊕**; 7**⊕**; 8**⊕**; 9**⊕**; 10**⊗**; 11**⊕**; 12**∅**; 13**⊕**; 14**⊗**: **T = 2** |
| Descamps *et al.* 2022 | 1**⊕**; 2**⊗**; 3**∅**; 4a**∅**; 4b**⊗**; 5**⊗**; 6**⊕**; 7**⊕**; 8**⊕**; 9**⊕**; 10**⊗**; 11**⊕**; 12**∅**; 13**⊕**; 14**⊗**: **T = 0** |
| De Miranda *et al.* 2022 ***^2^*** | 1**⊕**; 2**⊗**; 3**∅**; 4a**∅**; 4b**⊗**; 5**⊗**; 6**⊕**; 7**⊕**; 8**⊕**; 9**⊕**; 10**⊗**; 11**⊕**; 12**∅**; 13**⊕**; 14**⊗**: **T = 0** |
| De Vito *et al.* 2021 | 1**⊕**; 2**⊕**; 3**∅**; 4a**⊕**; 4b**⊕**; 5**⊗**; 6**⊕**; 7**⊕**; 8**⊗**; 9**⊗**; 10**⊗**; 11**⊕**; 12**∅**; 13**⊕**; 14**⊕**: **T = 3** |
| Díaz-Simón *et al.* 2021 | 1**⊕**; 2**⊗**; 3**∅**; 4a**∅**; 4b**⊗**; 5**⊗**; 6**⊕**; 7**⊕**; 8**⊕**; 9**⊕**; 10**⊗**; 11**⊕**; 12**∅**; 13**⊕**; 14**⊗**: **T = -2** |
| Diez-Quevedo *et al.* 2021 | 1**⊕**; 2**∅**; 3**∅**; 4a**∅**; 4b**∅**; 5**⊗**; 6**⊕**; 7**⊕**; 8**⊕**; 9**⊕**; 10**⊗**; 11**⊕**; 12**∅**; 13**⊕**; 14**⊕**: **T = 4** |
| Durstenfeld *et al.* 2022 ***^2^*** | 1**⊕**; 2**⊕**; 3**⊕**; 4a**⊕**; 4b**⊗**; 5**⊗**; 6**⊕**; 7**⊕**; 8**⊕**; 9**⊕**; 10**∅**; 11**⊕**; 12**∅**; 13**⊕**; 14**⊕**: **T = 5** |
| Egede *et al.* 2021 | 1**⊕**; 2**⊕**; 3**⊕**; 4a**⊕**; 4b**⊗**; 5**⊗**; 6**⊕**; 7**⊕**; 8**⊕**; 9**⊕**; 10**∅**; 11**⊕**; 12**∅**; 13**⊕**; 14**⊕**: **T = 7** |
| Fond *et al.* 2021 | 1**⊕**; 2**⊕**; 3**⊕**; 4a**⊕**; 4b**⊕**; 5**⊗**; 6**⊕**; 7**⊕**; 8**⊕**; 9**⊗**; 10**∅**; 11**⊕**; 12**∅**; 13**⊕**; 14**⊕**: **T = 9** |
| Francis *et al.* 2021 | 1**⊕**; 2**∅**; 3**∅**; 4a**⊕**; 4b**∅**; 5**⊕**; 6**⊕**; 7**⊕**; 8**⊗**; 9**∅**; 10**∅**; 11**⊕**; 12**∅**; 13**⊕**; 14**⊕**: **T = 7** |
| Garcia‑Cabrera *et al.* 2021 | 1**⊕**; 2**∅**; 3**∅**; 4a**⊕**; 4b**∅**; 5**⊕**; 6**⊕**; 7**⊕**; 8**⊗**; 9**∅**; 10**∅**; 11**⊕**; 12**∅**; 13**⊕**; 14**⊕**: **T = 3** |
| Gasnier *et al.* 2022 | 1**⊕**; 2**∅**; 3**∅**; 4a**⊕**; 4b**∅**; 5**⊕**; 6**⊕**; 7**⊕**; 8**⊗**; 9**∅**; 10**∅**; 11**⊕**; 12**∅**; 13**⊕**; 14**⊕**: **T = 5** |
| Giannoglou *et al.* 2021 | 1**⊕**; 2**⊗**; 3**∅**; 4a**∅**; 4b**∅**; 5**⊗**; 6**⊕**; 7**⊕**; 8**⊕**; 9**⊕**; 10**⊗**; 11**⊕**; 12**∅**; 13**⊕**; 14**⊗**: **T = -3** |
| Goldberger *et al.* 2022 | 1**⊕**; 2**⊗**; 3**∅**; 4a**∅**; 4b**⊗**; 5**⊗**; 6**⊕**; 7**⊕**; 8**⊕**; 9**⊕**; 10**⊗**; 11**⊕**; 12**∅**; 13**⊕**; 14**⊗**: **T = 0** |
| Haimovich *et al.* 2020 | 1**⊕**; 2**⊗**; 3**∅**; 4a**∅**; 4b**⊗**; 5**⊗**; 6**⊕**; 7**⊕**; 8**⊕**; 9**⊕**; 10**⊗**; 11**⊕**; 12**∅**; 13**⊕**; 14**⊗**: **T = 0** |
| Hashemi‑Shahri *et al.* 2022 | 1**⊕**; 2**⊗**; 3**∅**; 4a**∅**; 4b**⊗**; 5**⊗**; 6**⊕**; 7**⊕**; 8**⊕**; 9**⊕**; 10**⊗**; 11**⊕**; 12**∅**; 13**⊕**; 14**⊗**: **T = 0** |
| Hedberg *et al.* 2023 ***^2^*** | 1**⊕**; 2**⊗**; 3**∅**; 4a**∅**; 4b**⊗**; 5**⊗**; 6**⊕**; 7**⊕**; 8**⊕**; 9**⊕**; 10**⊗**; 11**⊕**; 12**∅**; 13**⊕**; 14**⊗**: **T = 0** |
| Hirashima *et al.* 2021 | 1**∅**; 2**⊗**; 3**∅**; 4a**∅**; 4b**∅**; 5**⊗**; 6**⊕**; 7**⊕**; 8**⊕**; 9**⊕**; 10**⊗**; 11**⊕**; 12**∅**; 13**⊕**; 14**⊗**: **T = -2** |
| Izurieta *et al.* 2020 | 1**⊕**; 2**⊕**; 3**⊕**; 4a**⊕**; 4b**⊕**; 5**⊗**; 6**⊕**; 7**⊕**; 8**⊕**; 9**⊕**; 10**∅**; 11**⊕**; 12**∅**; 13**⊕**; 14**⊗**: **T = 5** |
| Jeon *et al.* 2020 | 1**⊕**; 2**⊕**; 3**⊕**; 4a**⊕**; 4b**⊕**; 5**⊗**; 6**⊕**; 7**⊕**; 8**⊕**; 9**⊕**; 10**∅**; 11**⊕**; 12**∅**; 13**⊕**; 14**⊕**: **T = 9** |
| Jones *et al.* 2020 | 1**⊕**; 2**⊕**; 3**∅**; 4a**∅**; 4b**∅**; 5**⊗**; 6**⊕**; 7**⊕**; 8**⊕**; 9**⊕**; 10**∅**; 11**⊕**; 12**∅**; 13**⊕**; 14**⊗**: **T = 4** |
| Kundi *et al.* 2020 | 1**⊕**; 2**⊕**; 3**⊕**; 4a**⊕**; 4b**⊕**; 5**⊗**; 6**⊕**; 7**⊕**; 8**⊕**; 9**⊕**; 10**∅**; 11**⊕**; 12**∅**; 13**⊕**; 14**⊗**: **T = 5** |
| Lebin *et al.* 2020 | 1**⊕**; 2**⊕**; 3**∅**; 4a**∅**; 4b**∅**; 5**⊗**; 6**⊕**; 7**⊕**; 8**⊕**; 9**⊕**; 10**∅**; 11**⊕**; 12**∅**; 13**⊕**; 14**⊗**: **T = 4** |
| Lee *et al.* 2021 (1) | 1**⊕**; 2**⊕**; 3**⊕**; 4a**⊕**; 4b**⊕**; 5**⊕**; 6**⊕**; 7**⊕**; 8**⊕**; 9**⊕**; 10**∅**; 11**⊕**; 12**∅**; 13**⊕**; 14**⊕**: **T = 12** |
| Lee *et al.* 2021 (2) | 1**⊕**; 2**⊕**; 3**⊕**; 4a**⊕**; 4b**⊕**; 5**⊗**; 6**⊕**; 7**⊕**; 8**⊗**; 9**∅**; 10**∅**; 11**⊕**; 12**∅**; 13**⊕**; 14**⊕**: **T = 6** |
| Lega *et al.* 2021 | 1**⊕**; 2**⊕**; 3**∅**; 4a**∅**; 4b**∅**; 5**⊗**; 6**⊕**; 7**⊕**; 8**⊕**; 9**⊕**; 10**∅**; 11**⊕**; 12**∅**; 13**⊕**; 14**⊕**: **T = 4** |
| Li *et al.* 2021 | 1**⊕**; 2**⊗**; 3**∅**; 4a**∅**; 4b**⊗**; 5**⊗**; 6**⊕**; 7**⊕**; 8**⊕**; 9**⊕**; 10**⊗**; 11**⊕**; 12**∅**; 13**⊕**; 14**⊗**: **T = 2** |
| Maripuu *et al.* 2021 | 1**⊕**; 2**⊕**; 3**⊕**; 4a**⊕**; 4b**⊕**; 5**⊗**; 6**⊕**; 7**⊕**; 8**⊗**; 9**⊗**; 10**∅**; 11**⊕**; 12**∅**; 13**⊕**; 14**⊗**: **T = 3** |
| Meinschmidt *et al.* 2022 | 1**⊕**; 2**⊕**; 3**⊕**; 4a**⊕**; 4b**⊕**; 5**⊗**; 6**⊕**; 7**⊕**; 8**⊗**; 9**⊗**; 10**∅**; 11**⊕**; 12**∅**; 13**⊕**; 14**⊗**: **T = 7** |
| Merzon *et al.* 2020 | 1**⊕**; 2**⊕**; 3**⊕**; 4a**⊕**; 4b**⊕**; 5**⊗**; 6**⊕**; 7**⊕**; 8**⊗**; 9**⊗**; 10**∅**; 11**⊕**; 12**∅**; 13**⊕**; 14**⊕**: **T = 5** |
| Merzon *et al.* 2021 | 1**⊕**; 2**⊕**; 3**⊕**; 4a**⊕**; 4b**⊕**; 5**⊗**; 6**⊕**; 7**⊕**; 8**⊗**; 9**⊗**; 10**∅**; 11**⊕**; 12**∅**; 13**⊕**; 14**⊕**: **T = 5** |
| Musheyev *et al.* 2021 | 1**⊕**; 2**⊗**; 3**∅**; 4a**∅**; 4b**⊗**; 5**⊗**; 6**⊕**; 7**⊕**; 8**⊕**; 9**⊕**; 10**⊗**; 11**⊕**; 12**∅**; 13**⊕**; 14**⊗**: **T = 0** |
| Nemani *et al.* 2021 (1) | 1**⊕**; 2**⊕**; 3**⊕**; 4a**⊕**; 4b**⊕**; 5**⊗**; 6**⊕**; 7**⊕**; 8**⊗**; 9**⊕**; 10**∅**; 11**⊕**; 12**∅**; 13**⊕**; 14**⊕**: **T = 6** |
| Nemani *et al.* 2021 (2) | 1**⊕**; 2**⊗**; 3**∅**; 4a**∅**; 4b**⊗**; 5**⊗**; 6**⊕**; 7**⊕**; 8**⊕**; 9**⊕**; 10**⊗**; 11**⊕**; 12**∅**; 13**⊕**; 14**⊗**: **T = 0** |
| Nilsson *et al.* 2022 | 1**⊕**; 2**⊗**; 3**∅**; 4a**∅**; 4b**⊗**; 5**⊗**; 6**⊕**; 7**⊕**; 8**⊕**; 9**⊕**; 10**⊗**; 11**⊕**; 12**∅**; 13**⊕**; 14**⊗**: **T = 0** |
| Nishimi *et al.* 2021 ***^1^*** | 1**⊕**; 2**⊕**; 3**⊕**; 4a**⊕**; 4b**⊕**; 5**⊗**; 6**⊕**; 7**⊕**; 8**⊕**; 9**⊗**; 10**∅**; 11**⊕**; 12**∅**; 13**⊕**; 14**⊗**: **T = 7** |
| Orlando *et al.* 2021 | 1**⊕**; 2**⊕**; 3**⊕**; 4a**⊕**; 4b**⊕**; 5**⊗**; 6**⊕**; 7**⊕**; 8**⊗**; 9**⊗**; 10**∅**; 11**⊕**; 12**∅**; 13**⊕**; 14**⊕**: **T = 5** |
| Pavarin *et al.* 2022 | 1**⊕**; 2**⊗**; 3**∅**; 4a**∅**; 4b**⊗**; 5**⊗**; 6**⊕**; 7**⊕**; 8**⊕**; 9**⊕**; 10**⊗**; 11**⊕**; 12**∅**; 13**⊕**; 14**⊗**: **T = 0** |
| Poblador-Plou *et al.* 2021 | 1**⊕**; 2**⊕**; 3**⊕**; 4a**⊕**; 4b**⊕**; 5**⊗**; 6**⊕**; 7**⊕**; 8**⊕**; 9**⊕**; 10**∅**; 11**⊕**; 12**∅**; 13**⊕**; 14**∅**: **T = 9** |
| Qeadan *et al.* 2021 | 1**⊕**; 2**⊕**; 3**⊕**; 4a**⊕**; 4b**⊕**; 5**⊗**; 6**⊕**; 7**⊕**; 8**⊗**; 9**⊕**; 10**∅**; 11**⊕**; 12**∅**; 13**⊕**; 14**⊕**: **T = 7** |
| Rodríguez-M. *et al.* 2020 | 1**⊕**; 2**⊕**; 3**⊕**; 4a**⊕**; 4b**⊕**; 5**⊗**; 6**⊕**; 7**⊕**; 8**⊕**; 9**∅**; 10**∅**; 11**⊕**; 12**∅**; 13**⊕**; 14**⊗**: **T = 6** |
| Salvatore *et al.* 2021 | 1**⊕**; 2**⊕**; 3**⊕**; 4a**⊕**; 4b**⊕**; 5**⊗**; 6**⊕**; 7**⊕**; 8**⊕**; 9**⊕**; 10**∅**; 11**⊕**; 12**∅**; 13**⊕**; 14**⊕**: **T = 9** |
| Sisó-Almirall *et al.* 2020 | 1**⊕**; 2**⊕**; 3**∅**; 4a**∅**; 4b**⊕**; 5**⊗**; 6**⊕**; 7**⊕**; 8**⊗**; 9**⊗**; 10**∅**; 11**⊕**; 12**∅**; 13**⊕**; 14**∅**: **T = 3** |
| Tang *et al.* 2020 | 1**⊕**; 2**∅**; 3**⊕**; 4a**⊕**; 4b**⊕**; 5**⊗**; 6**⊕**; 7**⊕**; 8**⊗**; 9**⊗**; 10**∅**; 11**⊕**; 12**∅**; 13**⊕**; 14**⊕**: **T = 4** |
| Taquet *et al.* 2021 | 1**⊕**; 2**⊕**; 3**⊕**; 4a**⊕**; 4b**⊕**; 5**⊗**; 6**⊕**; 7**⊕**; 8**⊗**; 9**⊕**; 10**∅**; 11**⊕**; 12**∅**; 13**⊕**; 14**⊕**: **T = 6** |
| Teixeira *et al.* 2021 | 1**⊕**; 2**⊗**; 3**∅**; 4a**∅**; 4b**⊗**; 5**⊗**; 6**⊕**; 7**⊕**; 8**⊕**; 9**⊕**; 10**⊗**; 11**⊕**; 12**∅**; 13**⊕**; 14**⊗**: **T = 0** |
| Thompson *et al.* 2022 ***^2^*** | 1**⊕**; 2**⊗**; 3**∅**; 4a**∅**; 4b**⊗**; 5**⊗**; 6**⊕**; 7**⊕**; 8**⊕**; 9**⊕**; 10**⊗**; 11**⊕**; 12**∅**; 13**⊕**; 14**⊗**: **T = -2** |
| Tokuda *et al.* 2023 | 1**⊕**; 2**⊗**; 3**∅**; 4a**∅**; 4b**⊗**; 5**⊗**; 6**⊕**; 7**⊕**; 8**⊕**; 9**⊕**; 10**⊗**; 11**⊕**; 12**∅**; 13**⊕**; 14**⊗**: **T = 0** |
| Tzur Bitan *et al.* 2021 | 1**⊕**; 2**⊕**; 3**⊕**; 4a**⊕**; 4b**⊕**; 5**⊗**; 6**⊕**; 7**⊕**; 8**⊗**; 9**⊗**; 10**∅**; 11**⊕**; 12**∅**; 13**⊕**; 14**⊕**: **T = 4** |
| Varela-Rodríguez *et al.* 2021 | 1**⊕**; 2**⊕**; 3**∅**; 4a**∅**; 4b**∅**; 5**⊗**; 6**⊕**; 7**⊕**; 8**⊗**; 9**⊕**; 10**∅**; 11**⊕**; 12**∅**; 13**⊕**; 14**⊕**: **T = 4** |
| Velásquez-García *et al.* 2021 | 1**⊕**; 2**⊗**; 3**∅**; 4a**∅**; 4b**⊗**; 5**⊗**; 6**⊕**; 7**⊕**; 8**⊕**; 9**⊕**; 10**⊗**; 11**⊕**; 12**∅**; 13**⊕**; 14**⊗**: **T = 0** |
| Vrotsou *et al.* 2021 | 1**⊕**; 2**⊕**; 3**⊕**; 4a**⊕**; 4b**⊕**; 5**⊗**; 6**⊕**; 7**⊕**; 8**⊕**; 9**⊕**; 10**∅**; 11**⊕**; 12**∅**; 13**⊕**; 14**⊗**: **T = 7** |
| Wang *et al.* 2021 (1) | 1**⊕**; 2**⊕**; 3**⊕**; 4a**⊕**; 4b**⊕**; 5**⊗**; 6**⊕**; 7**⊕**; 8**⊕**; 9**⊕**; 10**∅**; 11**⊕**; 12**∅**; 13**⊕**; 14**⊕**: **T = 9** |
| Wang *et al.* 2021 (2) | 1**⊕**; 2**⊕**; 3**⊕**; 4a**⊕**; 4b**⊕**; 5**⊗**; 6**⊕**; 7**⊕**; 8**⊕**; 9**⊕**; 10**∅**; 11**⊕**; 12**∅**; 13**⊕**; 14**⊗**: **T = 9** |
| Wang *et al.* 2021 (3) | 1**⊕**; 2**⊗**; 3**∅**; 4a**∅**; 4b**⊗**; 5**⊗**; 6**⊕**; 7**⊕**; 8**⊕**; 9**⊕**; 10**⊗**; 11**⊕**; 12**∅**; 13**⊕**; 14**⊗**: **T = 0** |
| Wang *et al.* 2022 (1) | 1**⊕**; 2**⊗**; 3**∅**; 4a**∅**; 4b**⊗**; 5**⊗**; 6**⊕**; 7**⊕**; 8**⊕**; 9**⊕**; 10**⊗**; 11**⊕**; 12**∅**; 13**⊕**; 14**⊗**: **T = 2** |
| Wang *et al.* 2022 (2) | 1**⊕**; 2**⊗**; 3**∅**; 4a**∅**; 4b**⊗**; 5**⊗**; 6**⊕**; 7**⊕**; 8**⊕**; 9**⊕**; 10**⊗**; 11**⊕**; 12**∅**; 13**⊕**; 14**⊗**: **T = 2** |
| Wang *et al.* 2022 (3) | 1**⊕**; 2**⊗**; 3**∅**; 4a**∅**; 4b**⊗**; 5**⊗**; 6**⊕**; 7**⊕**; 8**⊕**; 9**⊕**; 10**⊗**; 11**⊕**; 12**∅**; 13**⊕**; 14**⊗**: **T = 0** |
| Welch *et al.* 2021 | 1**⊕**; 2**⊕**; 3**∅**; 4a**⊗**; 4b**⊕**; 5**⊗**; 6**⊕**; 7**⊕**; 8**⊕**; 9**⊕**; 10**∅**; 11**⊕**; 12**∅**; 13**⊕**; 14**⊕**: **T = 2** |
| Yang *et al.* 2020 | 1**⊕**; 2**⊕**; 3**∅**; 4a**∅**; 4b**∅**; 5**⊗**; 6**⊕**; 7**⊕**; 8**⊕**; 9**⊕**; 10**∅**; 11**⊕**; 12**∅**; 13**⊕**; 14**⊕**: **T = 6** |
| Yanover *et al.* 2020 | 1**⊕**; 2**⊕**; 3**∅**; 4a**∅**; 4b**∅**; 5**⊗**; 6**⊕**; 7**⊕**; 8**⊕**; 9**⊕**; 10**∅**; 11**⊕**; 12**∅**; 13**⊕**; 14**⊗**: **T = 4** |

**⊕** = yes; **∅** = neutral / don’t know; **⊗** = no

**Table S7.** Results from frequentist and Bayesian meta-analyses on infection risk based on local and nation wide data. See the Supplemental dataset (S1) for the studies included per analysis.

| *SARS-CoV-2 infection risk* | *K* ^a^ | *N* | OR (95% CI) | BF_10_ for OR = 1.00 ^b^ | *I^2^* | Egger’s *t* |
| --- | --- | --- | --- | --- | --- | --- |
| Anxiety disorders [1] | 4 | 3060903 | 1.20 (0.99 to 1.46) | 0.48 + H_0_ | 95.0 *** | 8.54 |
| Neurodevelopmental disorders [1] | 4 | 566411 | 1.29 (0.87 to 1.90) | 0.79 + H_0_ | 85.8 *** | 0.72 |
| Mood disorders [1] | 7 | 9247673 | 0.87 (0.68 to 1.11) | 0.86 + H_0_ | 98.3 *** | 12.5 |
| Mood disorders [2] | 10 | 68492565 | 0.96 (0.64 to 1.44) | 0.31 ++ H_0_ | 99.4 *** | -1.62 |
| Psychosis spectrum disorder [1] | 6 | 61783950 | 1.06 (0.80 to 1.41) | 0.31 ++ H_0_ | 89.1 *** | 4.85** |
| Psychosis spectrum disorder [2] | 8 | 3075141 | 1.33 (0.99 to 1.79) | 1.53 + H_1_ | 91.7 *** | 4.51** |
| Substance use disorder [1] | 9 | 78236869 | 1.09 (0.75 to 1.59) | 0.32 ++ H_0_ | 99.6 *** | 1.06 |
| Substance use disorder [2] | 8 | 78183925 | 1.12 (0.78 to 1.61) | 0.34 + H_0_ | 99.6 *** | 1.53 |
| Mix/other [1] | 11 | 7354951 | 1.09 (0.81 to 1.47) | 0.30 ++ H_0_ | 99.2 *** | -1.62 |
| Mix/other [2] | 9 | 5655910 | 0.95 (0.78 to 1.57) | 0.04 +++ H_0_ | 92.5 *** | -0.88 |

**Table S8.** Results of moderation analysis of infection risk presented as regression weight and SE for data-sets in which *k* ≥ 10 (see the manscript).

| *COVID-19 infection risk* | % female | Average age | Risk of bias | *N* |
| --- | --- | --- | --- | --- |
| Anxiety | *k* < 10 | *k* < 10 | *k* < 10 | *k* < 10 |
| Neurodevelopmental disorders | *k* < 10 | *k* < 10 | *k* < 10 | *k* < 10 |
| Mood disorders | -0.01 (0.02) | 0.03 (0.01) * | 0.01 (0.02) | -0.005 (0.02) |
| Psychosis spectrum | *k* < 10 | *k* < 10 | *k* < 10 | *k* < 10 |
| SUD | *k* < 10 | *k* < 10 | *k* < 10 | *k* < 10 |
| Mix/other | -0.04 (0.01) ** | 0.01 (0.01) | -0.02 (0.06) | -0.02 (0.03) |

**Table S9.** Results from frequentist and Bayesian meta-analyses on local and nation wide data. See the Supplemental dataset (S1) for the studies included per analysis.

| *COVID-19 severity* / course | *k* | *N* | OR (95% CI) | BF_10_ for OR = 1.00 ^a^ | *I^2^* | Egger’s *t* |
| --- | --- | --- | --- | --- | --- | --- |
| Anxiety disorder [1] | 4 | 100072 | 1.23 (1.12 to 1.36) *** | 1.53 + H_1_ | 24.0 | -0.04 |
| Neurodevelopmental disorders [1] | 1 | 1780 | 1.81 (1.29 to 2.25) ** | Not sufficient data | *No data* | *No data* |
| Mood disorders [1] | 7 | 250230 | 1.65 (1.22 to 2.23) ** | 9.37 ++ H_1_ | 88.3 *** | -1.30 |
| Psychosis spectrum disorder [1] | 5 | 244758 | 1.76 (1.06 to 2.92) * | 4.10 ++ H_1_ | 80.5 *** | -0.79 |
| Substance use disorder [1] | 7 | 122592 | 1.51 (1.24 to 1.83) *** | 193.41 ++++ H_1_ | 64.9 ** | 2.42 ** |
| Substance use disorder [2] | 6 | 152880 | 1.23 (1.11 to 1.34) *** | 17.89 +++ H_1_ | 92.6 *** | -0.79 |
| Substance use disorder [3] | 6 | 102922 | 1.73 (1.28 to 2.35) *** | 24.70 +++ H_1_ | 68.8 *8 | 2.48 ** |
| Mix/other [1] | 10 | 258947 | 1.35 (1.11 to 1.64) ** | 7.16 ++ H_1_ | 56.9 * | 0.65 |
| *COVID-19 hospitalization* | *k* | *N* | OR (95% CI) | BF_10_ for OR = 1.00 ^a^ | *I^2^* | Egger’s *t* |
| Anxiety disorder [1] | 4 | 633041 | 1.44 (1.17 to 1.78) ** | 8.30 ++ H_1_ | 76.7 * | -4.63 |
| Anxiety disorder [2] | 3 | 580842 | 1.30 (0.92 to 1.83) | 0.64 + H_0_ | 88.8 * | -6.82 * |
| Neurodevelopmental disorders [1] | 1 | 1780 | 1.93 (1.06 to 3.51) * | *No data* | *No data* | *No data* |
| Mood disorders [1] | 6 | 28983811 | 1.63 (1.34 to 1.97) *** | 16.45 +++ H_1_ | 92.5 *** | 2.21 * |
| Mood disorders [2] | 6 | 3654145 | 1.65 (1.51 to 1.82) *** | 733.12 ++++ H_1_ | 34.7 | 0.92 |
| Mood disorders [3] | 11 | 4330466 | 1.44 (1.16 to 1.79) ** | 12.65 +++ H_1_ | 95.0 *** | 1.73 |
| Psychosis spectrum disorder [1] | 10 | 36522290 | 1.86 (1.32 to 2.62) *** | 160.10 ++++ H_1_ | 98.5 *** | -0.72 |
| Psychosis spectrum disorder [2] | 10 | 1431436 | 1.75 (1.26 to 2.41) *** | 115.25 ++++ H_1_ | 96.6 *** | -0.21 |
| Substance use disorder [1] | 7 | 914933 | 1.54 (1.44 to 1.69) *** | 82.88 ++++ H_1_ | 69.4 ** | -0.69 |
| Substance use disorder [2] | 6 | 950783 | 1.88 (1.29 to 2.20) *** | 10.51 +++ H_1_ | 92.2 *** | -0.07 |
| Substance use disorder [3] | 9 | 1579214 | 1.56 (1.30 to 1.86) *** | 7.92 ++ H_1_ | 93.6 *** | 0.49 |
| Mix/other [1] | 5 | 9319916 | 1.55 (1.16 to 2.07) ** | 2.89 + H_1_ | 98.1 *** | -0.76 |
| Mix/other [2] | 6 | 6699380 | 1.54 (1.17 to 2.03) ** | 4.87 ++ H_1_ | 97.6 *** | -0.57 |
| Mix/other [3] | 6 | 6267140 | 1.33 (0.95 to 1.85) | 0.64 + H_0_ | 97.8 *** | -0.85 |
| *COVID-19 ICU admission* | *k* | *N* | OR (95% CI) | BF_10_ for OR = 1.00 ^a^ | *I^2^* | Egger’s *t* |
| Anxiety disorder [1] | 2 | 100034 | 1.08 (0.76 to 1.54) | *No data* | *No data* | *No data* |
| Neurodevelopmental disorders [1] | 0 |  | | | | |
| Mood disorders [1] | 6 | 6246991 | 1.11 (0.84 to 1.46) | 0.31 ++ H_0_ | 49.9 | -0.6o |
| Psychosis spectrum disorder [1] | 6 | 290253 | 1.45 (0.98 to 2.15) | 1.02 + H_0_ | 93.6 ** | 0.90 |
| Substance use disorder [1] | 4 | 194035 | 1.59 (1.11 to 2.28) * | 8.30 ++ H_1_ | 83.0 *** | 0.02 |
| Mix/other [1] | 9 | 6384393 | 1.34 (1.02 to 1.75) * | 2.03 + H_1_ | 93.7 *** | -0.26 |
| Mix/other [2] | 8 | 6370529 | 1.28 (0.97 to 1.71) | 1.23 + H_1_ | 94.0 *** | -0.51 |
| *COVID-19 mortality* | *k* | *N* | OR (95% CI) | BF_10_ for OR = 1.00 ^a^ | *I^2^* | Egger’s *t* |
| Anxiety disorder [1] | 6 | 3215223 | 1.14 (0.72 to 1.80) | 0.49 + H_0_ | 91.4 *** | 0.07 |
| Anxiety disorder [2] | 9 | 694418 | 1.08 (0.81 to 1.41) | 0.30 ++ H_0_ | 79.8 *** | 0.70 |
| Neurodevelopmental disorders [1] | 2 | 4412 | 1.26 (0.77 to 2.05) | 0.65 + H_0_ | 0.0 | *No data* |
| Mood disorders [1] | 14 | 34395611 | 1.50 (1.31 to 1.71) *** | 1170.29 ++++ H_1_ | 79.2 *** | 1.67 |
| Mood disorders [2] | 14 | 36509695 | 1.49 (1.31 to 1.70) *** | 462.35 ++++ H_1_ | 77.1 *** | 1.80 * |
| Mood disorders [3] | 20 | 8662467 | 1.42 (1.24 to 1.63) *** | 878.38 ++++ H_1_ | 68.4 *** | 2.03 ** |
| Psychosis spectrum disorder [1] | 13 | 57137783 | 2.15 (1.68 to 2.75) *** | 1594.72 ++++ H_1_ | 88.4 *** | -0.08 |
| Psychosis spectrum disorder [2] | 14 | 798320 | 2.57 (1.63 to 4.04) *** | 1483.74 ++++ H_1_ | 90.9 *** | -0.23 |
| Substance use disorder [1] | 11 | 1094627 | 1.45 (1.12 to 1.87) *** | 101.02 ++++ H_1_ | 86.0 *** | 0.47 |
| Substance use disorder [2] | 14 | 785992 | 1.49 (1.21 to 1.84) *** | 97.52 ++++ H_1_ | 69.5 *** | 0.09 |
| Mix/other [1] | 20 | 6787842 | 1.26 (1.08 to 1.47) *** | 9.91 ++ H_1_ | 81.4 *** | -0.83 |
| Mix/other [2] | 21 | 12802505 | 1.32 (1.16 to 1.49) *** | 19.42 +++ H_1_ | 85.7 *** | -1.00 |
| Mix/other [3] | 21 | 7284738 | 1.32 (1.14 to 1.53) *** | 15.26 +++ H_1_ | 85.6 *** | -0.79 |
| Mix/other [4] | 21 | 6855656 | 1.34 (1.13 to 1.59) *** | 1.80 + H_1_ | 90.0 *** | -0.52 |
| Mix/other [5] | 22 | 6858209 | 1.21 (1.05 to 1.39) ** | 2.51 + H_1_ | 84.4 *** | -0.92 |

* *P* < .05, ** *P* < .01, *** *P* < .001

^a^ For more information see **dataset S1**

^b^ Evidence category for the results from Bayesian analyses: + anecdotal evidence for H**_0_** or H**_1_**; ++ moderate evidence for H**_0_** or H**_1_**; +++ strong evidence for H**_0_** or H**_1_** ; ++++ very strong evidence for H**_0_** or H**_1_**

**Table S10.** Results of moderation analysis of morbidity and mortality presented as regression weight and SE for data-sets in which *k* ≥ 10 (see the manuscript).

| *COVID-19 severity / course* | % female | Average age | Risk of bias | *N* |
| --- | --- | --- | --- | --- |
| Anxiety | *k* < 10 | *k* < 10 | *k* < 10 | *k* < 10 |
| Neurodevelopmental disorders | *k* < 10 | *k* < 10 | *k* < 10 | *k* < 10 |
| Mood disorders | *k* < 10 | *k* < 10 | *k* < 10 | *k* < 10 |
| Psychosis spectrum | *k* < 10 | *k* < 10 | *k* < 10 | *k* < 10 |
| SUD | *k* < 10 | *k* < 10 | *k* < 10 | *k* < 10 |
| Mix/other | -0.06 (0.02) ** | 0.01 (0.01) | 0.05 (0.03) | -0.0007 (0.0001) |
| *COVID-19 hospitalization* | % female | Average age | Risk of bias | *N* |
| Anxiety | *k* < 10 | *k* < 10 | *k* < 10 | *k* < 10 |
| Neurodevelopmental disorders | *k* < 10 | *k* < 10 | *k* < 10 | *k* < 10 |
| Mood disorders | 0.04 (0.05) | 0.003 (0.03) | 0.09 (0.16) | 0.00073 (0.0011) |
| Psychosis spectrum | -0.08 (0.14) | -0.05 (0.07) | -0.12 (0.63) | 0.003 (0.002) |
| SUD | 0.58 (0.28) * | 0.008 (0.04) | -0.26 (0.17) | 0.0003 (0.0006) |
| Mix/other | *k* < 10 | *k* < 10 | *k* < 10 | *k* < 10 |
| *COVID-19 ICU admission* | % female | Average age | Risk of bias | *N* |
| Anxiety | *k* < 10 | *k* < 10 | *k* < 10 | *k* < 10 |
| Neurodevelopmental disorders | *k* < 10 | *k* < 10 | *k* < 10 | *k* < 10 |
| Mood disorders | *k* < 10 | *k* < 10 | *k* < 10 | *k* < 10 |
| Psychosis spectrum | *k* < 10 | *k* < 10 | *k* < 10 | *k* < 10 |
| SUD | *k* < 10 | *k* < 10 | *k* < 10 | *k* < 10 |
| Mix/other | *k* < 10 | *k* < 10 | *k* < 10 | *k* < 10 |
| *COVID-19 mortality* | % female | Average age | Risk of bias | *N* |
| Anxiety | *k* < 10 | *k* < 10 | *k* < 10 | *k* < 10 |
| Neurodevelopmental disorders | *k* < 10 | *k* < 10 | *k* < 10 | *k* < 10 |
| Mood disorders | -0.009 (0.02) | -0.0006 (0.006) | 0.05 (0.04) | -0.0002 (0.0002) |
| Psychosis spectrum | 0.02 (0.05) | -0.02 (0.03) | 0.06 (0.20) | 0.0007 (0.0006) |
| SUD | 0.006 (0.03) | -0.01 (0.02) | -0.07 (0.06) | -0.007 (0.009) |
| Mix/other | 0.005 (0.004) | 0.00004 (0.0006) | -0.02 (0.02) | 0.0003 (0.0001) ** |
